# A Bayesian Prevalence‐Incidence Mixture Model for Screening Outcomes With Misclassification

**DOI:** 10.1002/sim.70433

**Published:** 2026-04-07

**Authors:** Thomas Klausch, Birgit I. Lissenberg‐Witte, Veerle M. H. Coupé

**Affiliations:** ^1^ Department of Epidemiology and Data Science Amsterdam University Medical Center, Vrije Universiteit Amsterdam Amsterdam North Holland the Netherlands; ^2^ Julius Center for Health Sciences and Primary Care, Department of Data Science and Biostatistics University Medical Center, Utrecht University Utrecht the Netherlands

**Keywords:** Bayesian, misclassification, mixture model, screening, survival

## Abstract

Screening and surveillance programs for cancer, such as colorectal cancer (CRC), often yield electronic health records (EHR) of screening time, test results, and covariates. We consider EHR from CRC surveillance of individuals who have a high cancer risk due to their family history. These individuals, therefore, receive regular colonoscopies with the goal of finding and removing adenomas, precursor lesions to CRC. Our objective is to estimate time to adenoma incidence and explore associations with covariates. However, in doing so, several challenges of the CRC surveillance EHR have to be addressed. Importantly, the adenoma events are interval‐censored, meaning the exact event times are unknown and only fall within intervals defined by colonoscopy visits. Furthermore, colonoscopies can miss adenomas due to human or technical error, leading to misclassification of individuals with adenomas as adenoma‐free. Finally, the EHR data include individuals with adenomas at baseline, termed prevalent cases. This prevalence status may be unobserved if the baseline colonoscopy is missing or fails to detect existing adenomas. To address these challenges in the CRC EHR, and screening data in general, we develop a new prevalence‐incidence mixture model (PIM) with a Bayesian estimation back‐end through data augmentation and regularization priors. We show how to fit the model, estimate cumulative incidence functions, and evaluate model fit using information criteria as well as a non‐parametric estimator. In extensive simulations, we show good performance of the model when informative priors on the test sensitivity are provided, which is usually possible. An implementation in the R package BayesPIM is provided.

**Abbreviations**
AFT: accelerated failure time model
CIF: cumulative incidence function
CRC: colorectal cancer
DAG: directed acyclical graph
EHR: electronic health records
FACTS: Dutch familial colorectal cancer surveillance
MAR: missing at random
MCMC: Markov chain Monte Carlo
PIM: prevalence‐incidence mixture model
WAIC: widely applicable information criterion

## 1 Introduction

Screening and surveillance programs aim to detect diseases, such as cancer, at an early stage to improve treatment outcomes. Electronic health records (EHR) from these programs provide valuable insights into disease progression and can be used to optimize screening schedules, such as personalizing test frequency and timing. This study focuses on EHR from colorectal cancer (CRC) surveillance among individuals with elevated risk due to family history (Section 2). These individuals undergo regular colonoscopies to detect and remove adenomas, which are precursors to CRC. Since individuals in this high‐risk group may develop adenomas more frequently and rapidly than the general population, estimating adenoma incidence time is critical to optimize surveillance. To this end, we develop a modeling framework with an accompanying R package called BayesPIM. Our model is a type of so‐called prevalence‐incidence mixture model (PIM) with a Bayesian estimation back‐end. The class of PIM was first suggested by Cheung et al. [1] and Hyun et al. [2]. The primary goal of their PIM was to model time to incidence when the disease status is ascertained at irregularly spaced discrete points in time (interval censoring) and a part of the population can have the (pre‐state) disease already at baseline (i.e., the point in time when an individual is first included in the study), which is called prevalence. This observation process was also present in the CRC EHR, because the CRC screening process led to interval censoring and individuals may have had adenomas already at inclusion into study; see Section 2.

Prevalence complicates the estimation of the interval‐censored incidence model, in particular if the prevalence status is not observed (latent) for some or all individuals in the EHR. In the Cheung‐Hyun PIM, the prevalence status is unobserved if no test is administered at baseline for at least a subset of the population and hence it is unknown for these individuals whether they have prevalent disease or not. Ignoring the issue of latent prevalence in standard interval‐censored survival models, such as the Accelerated Failure Time (AFT) model or the Cox model, causes underestimation of the time to incidence, because latent prevalent cases can be discovered after baseline and are then treated as incident cases. The Cheung‐Hyun PIM model solves this issue through putting point probability mass on incidence at baseline (time zero), denoting effectively immediate transition for prevalent cases. This modeling decision is similar to so‐called cure models that allow a proportion of the population to never progress, which is achieved by putting incidence probability mass on infinity (for a review of cure models, see [3]). The Cheung‐Hyun PIM jointly estimates the incidence and the prevalence model by an expectation maximization (EM) algorithm, implemented in the R package PIMixture [4] that is currently suggested as a principal modeling approach for screening data on the US National Cancer Institute (NCI) website [5]. The PIMixture methodology has been applied in numerous epidemiological studies, such as estimating time to CIN2/3 lesions with prevalence at baseline using cervical cancer screening EHR [6, 7]. The model has also been recently extended to take competing events into account [8].

Building on the PIM framework, our model BayesPIM extends the approach to account for imperfect test sensitivity (less than one), where sensitivity is the probability to find the (pre‐state) disease when it is truly present. This enhancement is motivated by the CRC EHR, where colonoscopies can miss adenomas due to technical or human error at baseline or during follow‐up (misclassification). The test sensitivity of a colonoscopy for a small to medium‐sized adenoma varies depending on the study between approximately 0.65 and 0.92 [9]. As PIMixture and standard models for interval‐censored survival data [10, 11] have been developed under the assumption of perfect test sensitivity, their estimates are prone to bias. Importantly, with imperfect tests, latent prevalence due to misclassification at baseline may occur even when all subjects receive a baseline test, while PIMixture assumes latent prevalence can only occur in subjects that do not receive a baseline test. BayesPIM handles latent prevalence regardless of whether its cause is an omitted baseline test or misclassification and can co‐estimate the test sensitivity. As we show, including prior information on the sensitivity is an advantage of the Bayesian approach, as it stabilizes estimation and incorporates prior uncertainty. While the model accommodates imperfect sensitivity, we assume perfect specificity, a reasonable assumption in CRC screening where initial findings are usually confirmed via pathology which corrects falsely positive tests.

BayesPIM allows for parametric survival distributions and uses model selection criteria to identify the best fit. In addition, we propose a joint non‐parametric estimator of the cumulative incidence function (CIF) and prevalence which can be used to validate model fit visually. Our procedure is based on the non‐parametric CIF estimator em_mixed developed by Witte et al. [12], originally intended for interval‐censored screening data with misclassification but without baseline prevalence. Using a recoding step similar to an approach described by Cheung et al. [1] to adapt the Turnbull [13] non‐parametric maximum likelihood (NPMLE) estimator to their PIM setting, we adapt em_mixed to the PIM setting with misclassification and prevalence.

Misclassification and prevalence have also been discussed in the context of multi‐state models. Specifically, continuous‐time hidden Markov models (HMM) allow modeling transition processes between multiple (disease) states, while specifying a probability distribution relating the observed states to underlying true states which may be used to account for imperfect test sensitivity [14, 15]. Bayesian HMM have been suggested that can additionally deal with prevalence [16, 17]. These models rely on the Markov assumption, meaning that the transition probability to a state does not depend on the time spent in the state which is akin to assuming an exponential transition time distribution (constant hazard function). We believe that this is too restrictive for many cancer screening settings, where hazards of (pre‐state) disease events may change across time. Semi‐Markov models have been suggested to account for the time‐dependence of transition rates [18, 19]. AFT models, applied in the present approach, are very similar to semi‐Markov models in this regard; for example, a Weibull AFT specification allows for non‐constant hazards of transition. Recently, Klausch et al. [20] used AFT models for three‐state screening data, demonstrating the robustness of Bayesian estimation when using regularization priors, similar as in BayesPIM (Section 3.4). However, these models do not take prevalence and misclassification into account.

This article is structured as follows. Section 2 introduces the Dutch CRC EHR as the motivating case of our methodology. Subsequently, Sections 3 and 4 describe the data‐generating mechanism and estimation methodology, respectively. Section 5 evaluates BayesPIM through simulations including settings created using resampling of the real‐world Dutch CRC data. Finally, Section 6 presents the application to the Dutch CRC EHR and Section 7 presents the discussion.

## 2 Motivating Case Study: The Dutch CRC EHR

Our motivating case involves CRC surveillance through colonoscopy in individuals with an elevated CRC risk due to family history. Specifically, we analyze a merged dataset (n=810) of EHR from two sources (Table 1). The primary dataset comes from the Dutch Familial Colorectal Cancer Surveillance (FACTS) randomized controlled trial [21], which experimentally compared two surveillance protocols in this high‐risk group (three‐ versus six‐year intervals between colonoscopies). This was supplemented with observational EHR from a similar high‐risk population undergoing CRC surveillance according to current guidelines. These additional data were collected through ongoing research at two Dutch hospitals, Radboud UMC Nijmegen and Rijnstate Arnhem. Eligible participants for this case study were those with at least one successful colonoscopy, with baseline defined as the time of the first colonoscopy. The primary objective was to estimate the time from baseline to the first adenoma. However, given the progressive nature of CRC, a cancer may occasionally be detected instead of an adenoma during surveillance. In this study, the number of cancers was negligible (n=8, 1%). Since we consider the time until the first adenoma, in this study, follow‐up was censored at the first positive test if an adenoma was found.

**TABLE 1:** Baseline characteristics of the Dutch CRC EHR screening data (time is measured in years).

Statistic	Total	Event in follow‐up	
		Adenoma	Right censored
Sample size n	810	330	480
Confirmed prevalent (column %)	20.4	50.0	0
Baseline test unsuccessful (column %)	6.5	6.7	6.5
Censoring time (min/median/max)	1.2/6.0/30.1	1.2/6.3/22.1	1.4/6.0/30.1
No. of screenings (min/median/max)	1/1.5/7	1/1.5/7	1/2/7
Interval length (min/median/max)	1.0/3.1/20.3	1/3.3/13.7	1/3.1/20.3
Age at baseline (min/median/max)	24.3/53.2/86.3	27.6/54.7/86.3	24.26/52.3/73.5
Gender is female (column %)	55.8	53.0	57.8

At baseline, 20.4% (n=165) of individuals were found to have an adenoma. Since any finding during colonoscopy is verified by pathology we assume that the combined test has perfect specificity (cf. Section 1). Hence, 20.4% of individuals have confirmed (i.e., known) prevalence (Table 1). However, in the remaining 79.6% (n=645), the prevalence status was unknown (latent) due to two possible reasons. First, although every individual received a colonoscopy at baseline, in 6.5%, the baseline colonoscopy was not completed successfully, due to, for example, incomplete visualization of the colon. These test outcomes are categorized as missing. In the remaining cases, while the baseline colonoscopy was successfully performed and returned a negative result, it may have missed an adenoma (misclassification). Thus, although the baseline non‐completion rate was relatively low (6.5%), substantial uncertainty regarding baseline prevalence remains due to the potential for missed adenomas.

There were six types of surveillance visit patterns present in the CRC EHR that are processed by BayesPIM (Table 2). Table 2 gives an example data vector for all types using four screening moments (i.e., time points $v_{1}$–$v_{4}$). For convenience, we always set $v_{1}=0$ denoting baseline; whether the result of a colonoscopy is available at baseline is indicated by $r_{i}$, with $r_{i}=1$ if yes and $r_{i}=0$ if not. The result may be missing because no colonoscopy was conducted at baseline (so called “no‐show”) or because the colonoscopy was unsuccessful (e.g., incomplete imaging of the colon). We address all resulting pattern types in turn. First, incident cases in the follow‐up can occur after a successful baseline colonoscopy (Type 1, 17.7%). The data example shows that incidence was found at $v_{3}=6$ years. The adenoma thus surely occurred before 6 years, and, more specifically, it occurred in the (3.0,6.0] interval if it was not missed at the colonoscopy at $v_{2}=3$ years, or it occurred in (0,3.0] if it was not missed at the $v_{1}=0$ baseline colonoscopy. Whether the adenoma was missed at any test occasion is unknown. Second, right censoring (loss to follow‐up) can occur after a successful baseline colonoscopy (Type 2, 46.5%). Here, we adopt the common notation that the last screening time is set to infinity in case of right censoring. The data example then suggests that right censoring occurred after the last colonoscopy had been performed at $v_{3}=6.3$ years. Note that due to misclassification right censoring may have occurred even if an adenoma had been present before $v_{3}=6.3$ including the possibility of a latent prevalent adenoma at baseline. To the contrary, Type 3 denotes prevalence found at baseline, as indicated by $v_{1}=0$ without further screening moments (20.4%; cf. Table 1). It is important to distinguish the prevalent case from Type 4 that denotes a negative baseline test with loss to follow‐up before the first surveillance moment in the regular follow‐up, as indicated by (0,∞) in the data example (8.9%). Again, Type 4 also might denote a prevalent case with falsely negative baseline test. Furthermore, note that case Types 3 and 4 do not provide information on incidence, but they do inform the estimation of the prevalence model. The final two Types 5 (2.7%) and 6 (3.8%), concern cases without baseline test ($r_{i}=0$) and an event in the follow‐up (until $v_{3}=8.3$ years) or loss to follow‐up (at $v_{2}=5.9$ years), respectively.

**TABLE 2:** Typology of screening cases in the Dutch CRC EHR with observed proportion per type (n=810; note: the data example uses up to four visits for illustration, but the real CRC EHR contained up to seven visits, see Table 1).

Type	Description	% Observed	Data example				
			$v_{1}$	$v_{2}$	$v_{3}$	$v_{4}$	$r_{i}$
1	Incident in follow‐up with baseline test	17.7	0	3.0	6.0		1
2	Right censored with baseline test	46.5	0	2.9	6.3	∞	1
3	Observed prevalent at baseline test	20.4	0				1
4	Right censored with only a baseline test	8.9	0	∞			1
5	Incident in follow‐up without a baseline test	2.7	0	6.2	8.3		0
6	Right censored without a baseline test	3.8	0	5.9	∞		0

## 3 BayesPIM: Data Generating Mechanism

We first introduce notation (Section 3.1) and give an overview of the hierarchical model structure of BayesPIM (Section 3.2). Subsequently, we give details on the PIM (Section 3.3) and the prior assumptions (Section 3.4).

### 3.1 Notation

Every individual i=1,…,n has an observed vector of ordered screening times $v_{i}=(v_{i1},v_{i2},\ldots ,v_{ic_{i}})$, with $v_{i1}<v_{i2}<\ldots <v_{ic_{i}}$ of length $c_{i}$, where we set $v_{i1}=0$ to denote baseline and $v_{ic_{i}}=\infty$ if the screening series is right censored, so that $v_{ic_{i}}<\infty$ only if the last screening time corresponds to a positive test (Table 2). The latent time of right censoring (loss to follow‐up) is denoted $s_{i}$. Right censoring occurs when $v_{ij+1}$ would be greater than $s_{i}$; see Section 3.2. The associated test outcomes at $v_{i}$ are $y_{i}=(y_{i1},y_{i2},\ldots ,y_{ic_{i}})$, where $y_{ij}=1$ denotes a positive test and $y_{ij}=0$ a negative test. Since we assume perfect test specificity (cf. Section 1) and are interested in the time until the first event/adenoma (cf. Section 2), a positive test at $v_{ic_{i}}<\infty$ implies that $y_{i}=(0,0,\ldots ,0,1)$ (i.e., a vector of length $c_{i}$ with zeros in all except the last position). For right censoring, we have that $y_{i}=(0,0,\ldots ,0,0)$ (i.e., a vector of length $c_{i}$ with zeros in all positions). Disease testing is administered at each scheduled screening time, including baseline when indicator $r_{i}=1$. If $r_{i}=0$, $y_{i1}$ is unavailable (missing) regardless of cause (e.g., missing test, unsuccessful test). Furthermore, every individual has covariates $x_{i}$ of dimension p×1, where $x_{i}$ is understood to have a unit entry in the first position (intercept). We write X for the n×p matrix of covariates. The observed data for individual i are thus ${𝒟}_{i}=\{r_{i},v_{i},x_{i},y_{i}\}$ wrapped as set $𝒟=\{{𝒟}_{1},\ldots ,{𝒟}_{n}\}$.

The latent transition time $t_{i}$ (incidence) is interval‐censored by $v_{i}$. The time $t_{i}$ is measured from baseline (i.e., time zero). Its units are implied by the unit of measurement of the screening times $v_{i}$ (e.g., days, months, or years). We model the time $t_{i}$ through an AFT model using $x_{i}$ (Section 3.3), parametrized by a coefficient vector $\beta \in {\mathbb{R}}^{p}$ and a scale parameter $\sigma \in {\mathbb{R}}^{+}$ (Section 3.3). We write $t=(t_{1},\ldots ,t_{n})$ for the vector of all transition times. Furthermore, we use latent variable $g_{i}$ to indicate the prevalence status at baseline, with $g_{i}=1$ indicating prevalence and $g_{i}=0$ indicating non‐prevalence. We model the prevalence status using covariates $x_{i}$, through a probit model (Section 3.3). The probit model is parametrized by coefficients $\theta \in {\mathbb{R}}^{p}$ and its linear term is denoted $\mu_{i}=x_{i}^{T}\theta$. We write $g=(g_{1},\ldots ,g_{n})$ for the vector of all prevalence status indicators. We note that while we generically refer to the covariates in the incidence and prevalence model as $x_{i}$, BayesPIM allows including different, possibly overlapping subsets of covariates in the two models. For notational ease, we use the short‐hand notation $x_{i}$ denoting that the same covariates are entered in both models. Furthermore, when $g_{i}=1$ (prevalence) the time $t_{i}$ is defined as a counterfactual. This means that $t_{i}$ is the time when transition to disease would occur if i, counter to fact, were non‐prevalent. This conceptualization enables joint Gibbs sampling of the latent (unobserved) $g_{i}$ and $t_{i}$; see Section 4.

We use latent variable $y_{ij}^{*}$ to indicate disease presence, taking value one if the disease is present at occasion j and zero otherwise, where

(1) $$\begin{array}{l} y_{ij}^{*}(g_{i},t_{i},v_{ij})=\left\{\begin{array}{ll} 1_{\left\{v_{ij}\geq t_{i}\right\}}\;\; & \text{if}\;g_{i}=0\;\text{(non‐prevalent)}\\ 1\;\; & \text{if}\;g_{i}=1\;\text{(prevalent)}. \end{array}\right. \end{array}$$

Using (1), the test sensitivity is defined as $\kappa =\mathrm{Pr}(y_{ij}=1|y_{ij}^{*}=1)$. We note that $g_{i}\equiv y_{i1}^{*}$ but for notational clarity, we keep $g_{i}$ as the prevalence mixture indicator that is modeled. A special case emerges when the baseline test is positive ($y_{i1}=1$). Due to the assumption of perfect test specificity (Section 1), it is then known that $g_{i}=y_{i1}^{*}=1$. However, $g_{i}$ remains latent when $y_{i1}=0$ (possibly falsely negative test) or when the baseline test is missing ($r_{i}=0$).

We denote probability density functions (PDF) and probability mass functions (PMF) with f; for example $f_{t}$ gives the PDF of $t_{i}$ and $f_{g}$ gives the PMF of the Bernoulli variable $g_{i}$. The cumulative distribution function (CDF) of $t_{i}$, also called the cumulative incidence function (CIF, defined as one minus the survival function), is denoted $F_{t}$. The CDF of the normal distribution is denoted Φ. Furthermore, the prior PDFs are denoted π with prior parameters $\tau_{\beta }$ (prior of β), λ (prior of σ), $\tau_{\theta }$ (prior of θ), and α (prior of κ); see Section 3.4.

### 3.2 Hierarchical Model Structure and Assumptions

**FIGURE 1:**
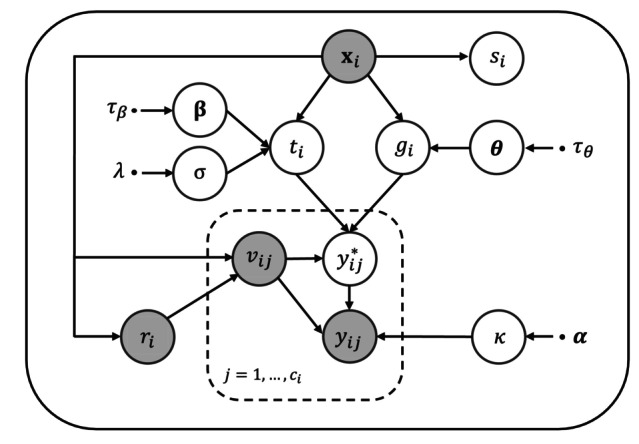
DAG illustrating the hierarchical model structure of BayesPIM using plate notation. White circles denote unobserved variables, grey circles denote observed variables, dots denote fixed parameters, and arrows denote the direction of dependence. For clarity, dependence of $v_{ij}$ on history ${\bar{v}}_{ij}$ is suppressed and $v_{i1}=0$ (baseline time) is implied.

We assume the following hierarchical model structure illustrated by a directed acyclic graph (DAG) in Figure 1. The parameters are generated from the prior (see Section 3.4)

$$\begin{array}{ll} \beta ,\sigma ,\theta ,\kappa |\tau_{\beta },\lambda ,\tau_{\theta },\alpha \; & ∼\;\pi (\beta ,\sigma ,\theta ,\kappa |\tau_{\beta },\lambda ,\tau_{\theta },\alpha ). \end{array}$$

For i=1,…,n and fixed vector $x_{i}$, the incidence time $t_{i}$, prevalence status $g_{i}$, baseline test decision $r_{i}$, and right censoring time $s_{i}$ are then generated as:

$$\begin{array}{ll} t_{i}|x_{i},\beta ,\sigma \; & ∼\;f_{t}(t_{i}|x_{i},\beta ,\sigma )\\ g_{i}|x_{i},\theta \; & ∼\;\text{Bernoulli}(\Phi (\mu_{i}))\\ r_{i}|x_{i}\; & ∼\;f_{r}(r_{i}|x_{i})\\ s_{i}|x_{i}\; & ∼\;f_{s}(s_{i}|x_{i}). \end{array}$$

In addition, we allow for the boundary cases $r_{i}=0$ for all i (nobody gets a baseline test) and $r_{i}=1$ for all i (everybody gets a baseline test), in which case $r_{i}$ is not random. The incidence time $\left(t_{i}\right)$ and prevalence $\left(g_{i}\right)$ models are discussed in more detail in Section 3.3.

Subsequently, for each i, the screening times $v_{i}$ and test outcomes $y_{i}$ are generated recursively (i.e., the observation process). Specifically, at baseline, indicator $r_{i}$ decides if a baseline test is done. For $r_{i}=0$, $y_{i1}$ is missing (latent) and screening continues as described below. Else, the baseline test outcome is drawn (recall $g_{i}=y_{i1}^{*}$ the prevalence status) as

$$\begin{array}{ll} y_{i1}|y_{i1}^{*},\kappa & ∼\text{Bernoulli}(\kappa \;y_{i1}^{*}). \end{array}$$

Hence, the baseline test cannot be positive in case of non‐prevalence ($\mathrm{Pr}(y_{i1}=1|y_{i1}^{*}=0,\kappa )=0$) and else has probability κ of being positive. If the baseline test is positive, screening terminates and we set $v_{i}=(v_{i1})=(0)$ with $y_{i}=(y_{i1})=(1)$ and $c_{i}=1$ (cf. Table 2, Type 3). Else, the following steps are repeated for j=2,3,… until an event (positive test) or right censoring; then we set $c_{i}=j$:

(2) $$\begin{array}{ll} v_{ij}|r_{i},{\bar{v}}_{ij},x_{i} & ∼f_{v}(v_{ij}|r_{i},{\bar{v}}_{ij},x_{i})\; \end{array}$$

(3) $$\begin{array}{ll} y_{ij}|v_{ij},y_{ij}^{*},\kappa & ∼\text{Bernoulli}(\kappa \;y_{ij}^{*}\;1_{\left\{v_{ij}<\infty \right\}}), \end{array}$$

with vector ${\bar{v}}_{ij}=(v_{i1},\overset{\ldots }{,}v_{ij-1})$ for j≥2 the screening times history before $v_{ij}$. Specifically, at any screening moment $j^{\prime }\geq 2$ we draw a candidate screening time $v_{ij^{\prime }}$ according to (2). If the screening time $v_{ij^{\prime }}$ would exceed the time of right censoring $s_{i}$ ($v_{ij^{\prime }}>s_{i}$), we set $c_{i}=j^{\prime }$ and $v_{ic_{i}}=\infty$ to indicate right censoring and we also set $y_{ic_{i}}=0$ with probability one; see (3). Else, we draw (3) and if the test is positive, $y_{ij^{\prime }}=1$, we set $c_{i}=j^{\prime }$, so that $v_{ic_{i}}<\infty$ is the last observed screening time. If $y_{ij^{\prime }}=0$ (negative test), screening continues with drawing a new screening time according to (2).

For model estimation, it is not necessary to parametrize the distributions of $r_{i}$, $s_{i}$ and $v_{ij}$; see Section 4. However, in Simulations 1 and 2 (Section 5) we give examples illustrating how all latent and observed model variables can be generated in simulation studies.

In summary, our model encodes the following key (conditional) independence assumptions (⟂ denoting independence):

- $t_{i}\;\;⟂\;g_{i}|x_{i}$ (Independence of prevalence‐incidence components)
- $r_{i}\;⟂\;(g_{i},t_{i})|x_{i}$ (Missing at random baseline test outcomes)
- $v_{ij}\;\;⟂\;(g_{i},t_{i})|(r_{i},{\bar{v}}_{ij},x_{i})$ (Uninformative scheduling of screening)
- $s_{i}\;⟂\;(g_{i},t_{i})|x_{i}$ (Uninformative censoring)
- $y_{ij}\;\;⟂\;(r_{i},{\bar{v}}_{ij},x_{i},{\bar{y}}_{ij})|(v_{ij},y_{ij}^{*},\kappa )$ (Stable test performance)

where ${\bar{y}}_{ij}=(y_{i1},\ldots ,y_{ij-1})$ is the history of screening outcomes. Assumptions (a) and (b) are similar to the missing at random (MAR) assumption [22] given $x_{i}$ while Assumption (c) denotes uninformative scheduling of screening tests and (d) uninformative right censoring. Assumption (e) implies that the screening test cannot be impacted by the timing or outcomes of past screenings and works equivalently for all individuals regardless of covariates $x_{i}$. We return to these assumptions in the Discussion (Section 7).

### 3.3 Prevalence‐incidence Mixture Model

The latent incidence time variable $t_{i}$ follows an AFT model

(4) $$\begin{array}{l} \log t_{i}=x_{i}^{T}\beta +\sigma \epsilon_{i}. \end{array}$$

The specific distribution chosen for the residuals $\epsilon_{i}$ induces the distribution $f_{t}(t_{i}|x_{i},\beta ,\sigma )$ through the change of variables defined by (4). For example, if $\epsilon_{i}$ is standard extreme value, logistic, or normal distributed, time $t_{i}$ is, respectively, Weibull, log‐logistical or log‐normal distributed (conditionally on $x_{i}$). These models allow time‐dependency of hazards of an event and are also called semi‐Markov models. An exponential (Markov‐type) model with constant hazards over time is obtained by choosing a Weibull distribution and constraining σ=1.

In addition, we model disease prevalence at baseline $\left(g_{i}\right)$ by a probit model where

(5) $$\begin{array}{l} \mathrm{Pr}(g_{i}=1|x_{i},\theta )=\Phi (x_{i}^{T}\theta )=\Phi (\mu_{i}). \end{array}$$

The probit model usually gives similar probability estimates as the equally common logistic model used by Cheung et al. [1] and Hyun et al. [2], where the two approaches differ primarily on the computational end. Specifically, a probit model is chosen because it allows conjugate normal parameter updates during model estimation which is fast (Section 4). For this, BayesPIM internally employs the latent variable formulation of the probit model, where a latent variable $w_{i}=\mu_{i}+\phi_{i}$, with $\phi_{i}\;∼\;N(0,1)$ and $g_{i}=1_{\left\{w_{i}>0\right\}}$, so that $\mathrm{Pr}(g_{i}=1|x_{i},\theta )=\mathrm{Pr}(w_{i}>0|x_{i},\theta )=\Phi (\mu_{i})$. The model residuals $\epsilon_{i}$ and $\phi_{i}$ are independent which induces the assumption of “independence of prevalence‐incidence components” conditional on $x_{i}$; see Section 3.2.

The CIF of time t conditional on non‐prevalence at baseline and a new user‐specified covariate vector $\tilde{x}$ is given by $F_{t}(t|g=0,\tilde{x},\beta ,\sigma )$. This CIF is interpreted as the cumulative risk of disease for healthy individuals at baseline. Furthermore, to describe the CIF of the mixture of prevalent and non‐prevalent cases we need to set the event time of prevalent cases to a value, where zero is the obvious choice, that is,

$$\begin{array}{l} t^{*}:=(1-g)t. \end{array}$$

Subsequently, we estimate the mixture CIF

$$\begin{array}{l} F_{t^{*}}(t|\tilde{x},\beta ,\sigma ,\theta )=\Phi ({\tilde{x}}^{T}\theta )+\left(1-\Phi ({\tilde{x}}^{T}\theta )\right)F_{t}(t|g=0,\tilde{x},\beta ,\sigma ). \end{array}$$

This CIF is similar to the CIF defined by Cheung et al. [1] who also put point probability mass at zero for prevalent cases. Furthermore, when we marginalize $F_{t}$ and $F_{t^{*}}$ over the distribution of the covariates we obtain marginal (population‐averaged) CIFs. Sections 4.3 and A.4 give further details on inference on these CIFs.

### 3.4 Prior Assumptions

The model parameters (β,σ,θ,κ) are random variables with independent prior distributions

$$\begin{array}{ll} \pi (\beta ,\sigma ,\theta ,\kappa |\tau_{\beta },\lambda ,\tau_{\theta },\alpha ) & =\left[\prod_{j=1}^{p}\pi (\beta_{j}|\tau_{\beta })\right]\\ & \;\times \left[\prod_{k=1}^{p}\pi (\theta_{k}|\tau_{\theta })\right]\pi (\sigma |\lambda )\pi (\kappa |\alpha ). \end{array}$$

Specifically, we choose zero‐centered standard normal priors for the regression parameters, that is, $\beta_{j}∼N(0,\tau_{\beta })$ and $\theta_{j}∼N(0,\tau_{\theta })$ with $\tau_{\beta }=\tau_{\theta }=1$. Furthermore, we specify a half‐normal prior with variance one for σ, that is, $\sigma ∼N^{+}(0,\lambda )$ with λ=1. These prior choices are called weakly informative, as they do not provide strong information on the location of the parameters, but do regularize parameter estimation. Regularization facilitates parameter estimation in sparse data settings that result from the interval and right censored observation process, the fact that in cancer screening there are typically few events, and latent prevalence [20]. Finally, for the sensitivity parameter κ we use a $\text{Beta}(\alpha_{1},\alpha_{2})$ prior, which can be chosen informatively. Alternatively, κ can be fixed at a known value (a point prior). We evaluate the performance of uninformative and informative prior choices for κ in simulations (Section 5). For an example, see the application (Section 6).

## 4 Model Estimation

In this section, we explain the estimation back‐end of BayesPIM. We begin by deriving the observed data likelihood, followed by details on the Gibbs sampling estimation procedure, posterior distribution of CIFs, and model fit evaluation.

### 4.1 Observed‐Data Likelihood

As we derive in detail in Section A.1, the observed‐data likelihood ℒ(β,σ,θ,κ|𝒟) is proportional to

(6) $$\begin{array}{ll} & \prod_{i\in {ℐ}_{0}}[(1-\Phi (\mu_{i}))\;\kappa^{y_{ic_{i}}}\sum_{j=1}^{c_{i}-1}{\left(1-\kappa \right)}^{\left(c_{i}-j-1\right)}\;\\ & \;\;\times [F_{t}(v_{ij+1}|x_{i},\beta ,\sigma )-F_{t}(v_{ij}|x_{i},\beta ,\sigma )]\\ & \;\;+\Phi (\mu_{i})\;\kappa^{y_{ic_{i}}}{\left(1-\kappa \right)}^{\left(c_{i}+r_{i}-2\right)}]\times \prod_{i\in {ℐ}_{1}}\Phi (\mu_{i})\;\kappa \end{array}$$

where ${ℐ}_{0}$ denotes the set of all individuals i with a negative baseline test ($r_{i}=1$ and $y_{i1}=0$) or a missing baseline test ($r_{i}=0$) and ${ℐ}_{1}$ denotes the set of all individuals i with a positive baseline test ($r_{i}=1$ and $y_{i1}=1$). As described in Section 3.1, a positive baseline test implies that prevalence is known with certainty ($g_{i}=1$). Therefore, known prevalent cases (${ℐ}_{1}$) inform the prevalence model through likelihood contribution $\Phi (\mu_{i})\kappa$ only and are not part of the mixture structure that applies to negative or missing baseline tests (${ℐ}_{0}$). This mixture form emerges, because the latent variables $g_{i}$ and $t_{i}$ are integrated out of the complete‐data likelihood.

Here, we give a brief summary of the results from Section A.1. In particular, the observed‐data likelihood is given by the complete‐data likelihood marginalized over latent $g_{i}$ and $t_{i}$ which, due to the factorization implied by the hierarchical model described in Section 3.2 and the DAG (Figure 1), is proportional to

(7) $$\begin{array}{ll} & \;\prod_{i=1}^{n}\sum_{l=0}^{1}\Phi {\left(\mu_{i}\right)}^{l}(1-\Phi {\left(\mu_{i})\right)}^{\left(1-l\right)}\\ & \;\times \int_{0}^{\infty }\mathrm{Pr}(y_{i}|g_{i}=l,r_{i},t_{i},v_{i},\kappa )\;f_{t}(t_{i}|x_{i},\beta ,\sigma )dt_{i}. \end{array}$$

Our goal is to obtain a closed‐form solution of the integral in (7). We first note that the probability term Pr(·) denotes the likelihood contribution of the test outcomes of individual i conditional on the latent $g_{i}$ and $t_{i}$, given by

(8) $$\begin{array}{ll} \mathrm{Pr}(y_{i}|g_{i},r_{i},t_{i},v_{i},\kappa ) & =\left\{\begin{array}{ll} 0\;\; & \text{if}\;g_{i}=0\;\text{and}\;t_{i}>v_{ic_{i}}\\ \kappa^{y_{ic_{i}}}{\left(1-\kappa \right)}^{m_{i}}\;\; & \text{if}\;g_{i}=0\;\text{and}\;t_{i}\leq v_{ic_{i}}\\ \kappa^{y_{ic_{i}}}{\left(1-\kappa \right)}^{\left(c_{i}+r_{i}-2\right)}\;\; & \text{if}\;g_{i}=1, \end{array}\right. \end{array}$$

with $m_{i}=\sum_{j=1}^{c_{i}-1}y_{ij}^{*}(g_{i},t_{i},v_{ij})$ the number of falsely negative tests. We note that, if $g_{i}=0$, likelihood (8) does not depend on the presence of a baseline test ($r_{i}$), because the disease is not present at baseline and we assume the test specificity to be perfect (Section 1). Also due to perfect specificity, the case $t_{i}>v_{ic_{i}}$ (if $g_{i}=0$) has a likelihood of zero whenever the last observed test is positive (i.e., $v_{ic_{i}}<\infty$) because then incidence must satisfy $t_{i}\leq v_{ic_{i}}$. Under right censoring we have $v_{ic_{i}}=\infty$, so no such upper bound applies. Technically, this zero‐probability acts as a constraint on the latent $t_{i}$ in the integral in (7). In particular, when $v_{ic_{i}}<\infty$ the l=0 term is effectively integrated over $\left(0,v_{ic_{i}}\right)$ rather than (0,∞).

To obtain a closed‐form solution for (7), we first note that (8) can also be written as a sum if $g_{i}=0$, that is,

(9) $$\begin{array}{l} \mathrm{Pr}(y_{i}|g_{i}=0,r_{i},t_{i},v_{i},\kappa )=\kappa^{y_{ic_{i}}}\sum_{j=1}^{c_{i}-1}{\left(1-\kappa \right)}^{\left(c_{i}-j-1\right)}1_{\left\{v_{ij}<t_{i}\leq v_{ij+1}\right\}}. \end{array}$$

Here, $\left(c_{i}-j-1\right)$ is the number of falsely negative tests applying if $t_{i}$ lies in the interval $\left(v_{ij},v_{ij+1}\right]$. Hence, (9) shows a computationally effective way of determining $m_{i}$ in (8). Furthermore, substituting (9) into (7) allows solving the integral and yields the final likelihood expression (6); for details see the Section A.1. The likelihood of the test outcomes (8) and its sum representation (9) also play a central role in the derivation of the full conditional distributions used by the Gibbs sampler, discussed next.

### 4.2 Gibbs Sampler

We are now interested in posterior inference on (β,σ,θ,κ) based on the posterior

$$\begin{array}{l} f_{\beta ,\sigma ,\theta ,\kappa }(\beta ,\sigma ,\theta ,\kappa |𝒟)\propto ℒ(\beta ,\sigma ,\theta ,\kappa |𝒟)\;\pi (\beta ,\sigma ,\theta ,\kappa |\tau_{\beta },\tau_{\theta },\lambda ,\alpha ). \end{array}$$

However, due to the mixture structure of the likelihood (6), direct sampling from the posterior is not feasible in closed form. Therefore, BayesPIM employs a Metropolis‐within‐Gibbs sampler with data augmentation. Equations ((10), (11), (12), (13), (14)) give the full conditional sampling steps for the random model variables and the parameters that are run repeatedly in sequence for k=1,…,K updates until convergence (see Sections 5 and 6 for details on the convergence criteria we applied). The conditional independences implied by the hierarchical model (Section 3.2) are already taken into account, and detailed derivations are given in Section A.2. Our sampler is implemented in the R package BayesPIM which is available from the Supporting Information, CRAN, and GitHub (https://github.com/thomasklausch2/BayesPIM).

After suitable initialization of the parameters, $g_{i}$, and $t_{i}$ (for i=1,…,n), the Gibbs sampler first performs data augmentation [23] of the latent variables:

(10) $$\begin{array}{ll} g_{i}^{\left(k+1\right)} & ∼f_{g}\left(g_{i}|{𝒟}_{i},\;\beta^{\left(k\right)},\;\sigma^{\left(k\right)},\;\theta^{\left(k\right)},\;\kappa^{\left(k\right)}\right),\;\\ & \;\text{if}\;r_{i}=0\;\text{or}\;(r_{i}=1\;\text{and}\;y_{i1}=0) \end{array}$$

(11) $$\begin{array}{l} t_{i}^{\left(k+1\right)}∼f_{t}\left(t_{i}|{𝒟}_{i},\;g_{i}^{\left(k+1\right)},\;\beta^{\left(k\right)},\;\sigma^{\left(k\right)},\kappa^{\left(k\right)}\right),\;\text{for all}\;i. \end{array}$$

These data augmentation steps are discussed in detail in Sections 4.2.1 and 4.2.2. Latent prevalence indicator $g_{i}$ is only augmented when it is missing, that is, without baseline test ($r_{i}=0$) or a negative baseline test ($r_{i}=1$ and $y_{i1}=0$). In case of a positive baseline test ($r_{i}=1$ and $y_{i1}=1$), $g_{i}=1$ is known due to the assumption of perfect specificity (see Sections 1 and 3.1). Here, we note already that step (10) marginalizes over $t_{i}$, which is also known as collapsing [24]; see Section 4.2.2 for a motivation.

The subsequent draws of the model parameters in the Gibbs sampler are performed conditional on the augmented (completed) data,

(12) $$\begin{array}{ll} {\left(\beta ,\sigma \right)}^{\left(k+1\right)} & ∼f_{\beta ,\sigma }\left(\beta ,\;\sigma |t^{\left(k+1\right)},X\right) \end{array}$$

(13) $$\begin{array}{ll} \theta^{\left(k+1\right)} & ∼f_{\theta }\left(\theta |g^{\left(k+1\right)},X\right) \end{array}$$

(14) $$\begin{array}{ll} \;\kappa^{\left(k+1\right)} & ∼f_{\kappa }\left(\kappa |𝒟,\;g^{\left(k+1\right)},\;t^{\left(k+1\right)}\right). \end{array}$$

Specifically, conditional on the augmented transition times $t^{\left(k+1\right)}$, the parameters β,σ in (12) are sampled from the complete‐data posterior distribution, which is proportional to $ℒ(\beta ,\sigma |t^{\left(k+1\right)},X)\;\pi (\beta ,\sigma |\tau_{\beta },\lambda )$, where $ℒ(\beta ,\sigma |t^{\left(k+1\right)},X)$ is the complete‐data likelihood. For drawing samples we employ a Metropolis step with normal proposal (jumping) distribution centered at the previous parameter draw $\left(\beta^{\left(k\right)},\sigma^{\left(k\right)}\right)$ and user‐specified proposal variance. An example for Weibull distributed $t_{i}$ is given in the Section A.2.4.

Furthermore, conditional on the augmented prevalence status $g^{\left(k+1\right)}$, the parameters θ in (13) are also drawn from a complete‐data posterior. For this step, BayesPIM internally exploits the latent variable formulation of the probit model (see Section 3.3). Specifically, after an additional data augmentation step of the latent probit variable $w_{i}$ for all i, parameters θ can be sampled from a conjugate normal distribution [23]; details are given in Section A.2.5. Updating the sensitivity parameter κ by (14) is optional; see Section 4.2.3.

#### 4.2.1 Augmenting the Transition Time

We first consider data augmentation of $t_{i}$ in step (11). The full conditional distribution of $t_{i}$ in the non‐prevalent case ($g_{i}=0$) is a finite mixture of non‐overlapping truncated distributions of $t_{i}$,

(15) $$\begin{array}{l} f_{t}(t_{i}|{𝒟}_{i},g_{i}=0,\beta ,\sigma ,\kappa )=\sum_{j=1}^{c_{i}-1}\omega_{ij}\;f_{t}(t_{i}|v_{ij}<t_{i}\leq v_{ij+1},x_{i},\beta ,\sigma ), \end{array}$$

where

$$\begin{array}{l} \omega_{ij}=\frac{{\tilde{\omega }}_{ij}}{\sum_{l=1}^{c_{i}-1}{\tilde{\omega }}_{il}}. \end{array}$$

and

(16) $$\begin{array}{l} {\tilde{\omega }}_{ij}=\kappa^{y_{ic_{i}}}{\left(1-\kappa \right)}^{\left(c_{i}-j-1\right)}\left[F_{t}(v_{ij+1}|x_{i},\beta ,\sigma )-F_{t}(v_{ij}|x_{i},\beta ,\sigma )\right], \end{array}$$

see Section A.2.2 for a proof. Specifically, factor ${\tilde{\omega }}_{ij}$ can be recognized as the weights of an unnormalized mixture distribution of $t_{i}$, while $\omega_{ij}$ are the normalized mixture weights yielding a normalized mixture distribution for $t_{i}$. It is interesting to observe that the mixture components of (15) are non‐overlapping, because the truncation bounds are equal to the screening times. With perfect test sensitivity, κ=1, the augmentation simplifies to sampling from the truncated distribution $f_{t}(t_{i}|v_{ic_{i}-1}<t_{i}\leq v_{ic_{i}},x_{i},\beta ,\sigma )$. This means that under perfect sensitivity, $t_{i}$ is known to lie in the most recent interval, $\left(v_{ic_{i}-1},v_{ic_{i}}\right]$ (in case of an event) or $\left(v_{ic_{i}-1},\infty \right)$ (in case of right censoring). For κ<1, the relative importance of a mixture component is determined by two main factors of (16). First, it decreases geometrically in the number of tests conducted since the most recent time $v_{ic_{i}}$, and, second, it increases with the probability mass of $t_{i}$ in the truncation interval. In general, more recent intervals are thus given higher weight, which is plausible.

Sampling from (15) is straightforward when viewed as a two‐stage sampling procedure for mixture distributions. First, sample a mixture component J from $J∼\text{categorical}(\omega_{i1},\ldots ,\omega_{i,c_{i}-1})$, where categorical denotes a draw of a class label J from the set $\left\{1,\ldots ,c_{i}-1\right\}$ with probabilities $\omega_{i1},\ldots ,\omega_{i,c_{i}-1}$. Second, use the associated interval bounds of the draw J as truncation bounds for $t_{i}$, that is, sample $t_{i}∼f_{t}(t_{i}|v_{iJ}<t_{i}\leq v_{iJ+1},x_{i},\beta ,\sigma )$.

In the prevalent case ($g_{i}=1$), the observed screening series contains no information on $t_{i}$, and hence

(17) $$\begin{array}{l} f_{t}(t_{i}|{𝒟}_{i},g_{i}=1,\beta ,\sigma )=f_{t}(t_{i}|x_{i},\beta ,\sigma ), \end{array}$$

so that $t_{i}$ is updated uninformatively.

#### 4.2.2 Augmenting the Prevalence Status

As we show in Section A.2.3, augmenting the unknown prevalence status $g_{i}$ from the full conditional distribution requires Bernoulli sampling with probability

(18) $$\begin{array}{ll} & \;f_{g}(g_{i}=1|{𝒟}_{i},t_{i},\theta ,\kappa )\;\\ & \;=\left\{\begin{array}{ll} \frac{\Phi (\mu_{i}){\left(1-\kappa \right)}^{\left(c_{i}+r_{i}-2\right)}}{\Phi (\mu_{i}){\left(1-\kappa \right)}^{\left(c_{i}+r_{i}-2\right)}+(1-\Phi (\mu_{i}))\kappa^{y_{ic_{i}}}{\left(1-\kappa \right)}^{m_{i}}}\;\; & \text{if}\;t_{i}\leq v_{ic_{i}}\\ 1\;\; & \text{if}\;t_{i}>v_{ic_{i}}. \end{array}\right. \end{array}$$

However, deterministic updating of $g_{i}$ with probability one if $t_{i}>v_{ic_{i}}$ in (18) can cause a dependency across the Gibbs sampling steps. Suppose we have augmentation $g_{i}^{\left(k\right)}=1$ at any point in the Gibbs sampler. Then $t_{i}$ is updated uninformatively in (0,∞) so that $t_{i}^{\left(k+1\right)}>v_{ic_{i}}$ can occur; see (17). In that case, $g_{i}^{\left(k+1\right)}=1$ with probability one again. This dependency continues unless in a future draw $t_{i}^{\left(k^{\prime }\right)}\leq v_{ic_{i}}$. To avoid this dependency we marginalize (18) over $t_{i}$, which is also known as collapsing [24]. A collapsed Gibbs sampler preserves the convergence properties of a full‐conditional Gibbs sampler but often reduces Markov chain Monte Carlo (MCMC) autocorrelation when data augmentation is used. As we show in Section A.2.3, collapsing $t_{i}$ yields fully stochastic Bernoulli updates with probability

(19) $$\begin{array}{l} f_{g}(g_{i}=1|{𝒟}_{i},\beta ,\sigma ,\theta ,\kappa )=\frac{\Phi (\mu_{i})\kappa^{y_{ic_{i}}}{\left(1-\kappa \right)}^{\left(c_{i}+r_{i}-2\right)}}{\begin{array}{l} \Phi (\mu_{i})\kappa^{y_{ic_{i}}}{\left(1-\kappa \right)}^{\left(c_{i}+r_{i}-2\right)}+(1-\Phi (\mu_{i}))\sum_{l=1}^{c_{i}-1}{\tilde{\omega }}_{il} \end{array}} \end{array}$$

where ${\tilde{\omega }}_{ij}$ is defined in (16). When testing the Gibbs sampler during development, we found that using (18) instead of (19) indeed caused apparent bias. All results in Sections 5 and 6 are, therefore, based on (19).

#### 4.2.3 Updating the Test Sensitivity Parameter

Updating the test sensitivity parameter κ is an optional feature of BayesPIM. A common alternative is fixing κ at a known value which can be viewed as a point prior at that value. In the simulations (Section 5), we evaluate estimation performance for different prior choices and data settings. For updating, κ can be viewed as the parameter of the test outcome likelihood (8) and, with a Beta prior on κ (see Section 3.4), the full conditional distribution is conjugate Beta,

$$\begin{array}{ll} f_{\kappa }\left(\kappa |𝒟,g^{\left(k+1\right)},t^{\left(k+1\right)}\right) & \propto \left[\prod_{i=1}^{n}\mathrm{Pr}(y_{i}|g_{i},r_{i},t_{i},v_{i},\kappa )\right]\pi (\kappa |\alpha )\\ & \propto \left[\prod_{i:\;g_{i}=0}\kappa^{y_{ic_{i}}}{\left(1-\kappa \right)}^{m_{i}}\right]\\ & \;\times \left[\prod_{i:\;g_{i}=1}\kappa^{y_{ic_{i}}}{\left(1-\kappa \right)}^{\left(c_{i}+r_{i}-2\right)}\right]\\ & \;\times \text{Beta}(\kappa |\alpha )\\ & \propto \text{Beta}\left(\kappa |Y+\alpha_{1},M+C-2G+\alpha_{2}\right), \end{array}$$

where $Y=\sum_{i=1}^{n}y_{ic_{i}}$, $M=\sum_{i:g_{i}=0}m_{i}$, $C=\sum_{i:g_{i}=1}(c_{i}+r_{i})$, $G=\sum_{i=1}^{n}g_{i}$. We note that due to the updating order in the Gibbs sampler where $g_{i}$ is updated first via (10) and $t_{i}$ is subsequently drawn from its full conditional, the test outcome likelihood (8) is strictly positive at all sampled states. Hence, the full conditional distribution for κ is always well‐defined.

### 4.3 Posterior Predictive Cumulative Incidence Functions

As described in Section 3.3, we draw inference on the conditional CIF $F_{t}(t|g=0,\tilde{x},\beta ,\sigma )$ and the mixture CIF $F_{t^{*}}(t|\tilde{x},\beta ,\sigma ,\theta )$ for fixed user‐specified covariate values $\tilde{x}$. In addition, we marginalize $F_{t}$ and $F_{t^{*}}$ over the empirical covariate distribution to obtain the marginal (i.e., population‐averaged) CIF $F_{t}(t|g=0,\beta ,\sigma )$ and the marginal mixture CIF $F_{t^{*}}(t|\beta ,\sigma ,\theta )$. For inference, we consider the CIF as functionals of the posterior distribution of the parameters. The posterior (mean) predictive conditional and marginal CIFs are then given by the posterior expectation of $F_{t}$ and $F_{t^{*}}$. For estimation and inference, we make use of the push‐forward transform of samples $\theta^{\left(k\right)},\beta^{\left(k\right)},\sigma^{\left(k\right)}$ via the Gibbs sampler into $F_{t}$ (or $F_{t^{*}}$), which yields samples from the posterior predictive CIF for any t. The expectation is obtained by the empirical average of the transformed samples. Furthermore, pointwise 95% credible (posterior) intervals are obtained by the 2.5% and 97.5% quantiles of the samples. For further details, see Section A.3.

### 4.4 Model Fit and Non‐Parametric Estimator of the Marginal CIF

To choose the best model specification in terms of the distribution of $t_{i}$ we use the widely applicable information criterion (WAIC) given by $-2[2{𝔼}_{\beta ,\sigma ,\theta ,\kappa |𝒟}\{\log ℒ(\beta ,\sigma ,\theta ,\kappa |𝒟)\}-\log{𝔼}_{\beta ,\sigma ,\theta ,\kappa |𝒟}\{ℒ(\beta ,\sigma ,\theta ,\kappa |𝒟)\}]$, where ℒ(β,σ,θ,κ|𝒟) is given by (6) and the expectations are estimated by Monte Carlo integration over the posterior samples of (β,σ,θ,κ) that are generated by the Gibbs sampler [25].

While the WAIC offers a relative measure of fit, we additionally suggest a non‐parametric estimator of the marginal mixture CIF $F_{t^{*}}(t|\beta ,\sigma ,\theta )$. This estimate can then be compared against the parametric BayesPIM estimate as a visual inspection of absolute goodness of fit. Specifically, Witte et al. [12] introduced a non‐parametric estimator for $F_{t}(t|g_{i}=0,\beta ,\sigma )$ for settings without prevalence but interval censoring and misclassification from imperfect screening tests, implemented in function em_mixed. To include baseline prevalence in em_mixed, our strategy is to apply a recoding step, similar to a preprocessing step suggested by Cheung et al. [1] to enable use of the Turnbull estimator [13] for estimating $F_{t^{*}}$ from interval‐censored data when the screening test sensitivity is one.

Concretely, we recode $v_{i}$ as follows. If $r_{i}=1$, then $v_{i1}=0$ is replaced by $\min\{v_{12},\ldots ,v_{n2}\}\times 0.01$ for all i. If $r_{i}=0$, we omit $v_{i1}=0$, so that $v_{i2}$ becomes the first screening time. Intuitively, this recoding treats the baseline test as if it occurred immediately after the actual baseline point. Consequently, if $r_{i}=0$ for a large proportion of i, the estimator may perform poorly because the anchoring to $\min[v_{12},\ldots ,v_{n2}]\times 0.01$ is not possible. We further investigate the performance of this estimator in the simulation section. We provide the adapted em_mixed estimator in R package EMmixed available in the Supporting Information and on GitHub (https://github.com/thomasklausch2/EMmixed).

## 5 Simulation Studies

We conducted two Monte Carlo simulations for evaluating the performance of BayesPIM compared to PIMixture and the non‐parametric em_mixed estimator. In Simulation 1, we generated synthetic data sets under different conditions repeatedly and evaluated the performance of the estimators. The primary purpose of these experiments was to evaluate the implementation of BayesPIM in a controlled setting with sufficient follow‐up moments to produce accurate parameter estimates. Simulation 2, to the contrary, was designed to generate data sets that closely resembled the CRC EHR (Section 2) to evaluate BayesPIM in a realistic screening setting.

### 5.1 Set‐Up of Simulation 1

We generated two covariates that were distributed $x_{1i}∼N(0,1)$ and $x_{2i}∼\text{Bernoulli}(0.5)$. The incidence model (4) was parametrized as $\beta ={\left(5,0.2,0.2\right)}^{T}$ and σ=0.2, so that

$$\begin{array}{l} \log t_{i}=5+0.2x_{1i}+0.2x_{2i}+0.2\epsilon_{i}, \end{array}$$

where the residual $\epsilon_{i}$ was extreme value distributed, so that $t_{i}|x_{i}$ was Weibull distributed. Together with σ=0.2 this yielded a semi‐Markov process (progression hazards increasing over time). Furthermore, the prevalence model (5) was parametrized as $\theta ={\left(\theta_{0},0.2,0.2\right)}^{T}$, with $\theta_{0}$ the intercept parameter that is varied across simulation conditions (see below), so that

$$\begin{array}{l} \mathrm{Pr}(g_{i}=1|x_{i},\theta )=\Phi (\theta_{0}+0.2x_{1i}+0.2x_{2i}). \end{array}$$

To generate screening times as in (2), we set $v_{i1}=0$ and then iteratively drew $v_{ij}$ for j≥2

$$\begin{array}{l} v_{ij}∼\text{uniform}(v_{ij-1}+20,v_{ij-1}+30), \end{array}$$

so that at least 20 and at maximum 30 time units elapsed between screening moments. Screening stopped if $y_{ij}=1$ generated by (3) with an event (positive test) or due to right censoring, as described in Section 3.2. The time of right censoring $s_{i}$ was distributed as

$$\begin{array}{l} s_{i}=v_{i2}+{\tilde{s}}_{i},\;{\tilde{s}}_{i}∼\text{exponential}(80^{-1}) \end{array}$$

so that the additional follow‐up time after the second screening had a mean of 80 time units. These choices allowed us to study BayesPIM with substantial follow‐up and facilitated that BayesPIM converged in acceptable time. In Simulation 2, Section 5.3, we examine a real‐world setting with less frequent screening and stronger right censoring adopted from the CRC EHR application.

Our simulation set‐up examined estimator performance in a wide range of scenarios concerning sample size, test sensitivity, baseline prevalence, baseline test presence, and prior distribution on the test sensitivity. Specifically, we compared two different sample sizes ($n_{sim}=1000$ versus $n_{sim}=2000$) and the true value of the test sensitivity parameter κ was set to a high level (κ=0.8), similar to the sensitivity of a colonoscopy for detection of an adenoma, or to a lower level (κ=0.4). Furthermore, the intercept parameter $\theta_{0}$ was employed to control the prevalence rates, where $\theta_{0}=0.11$ and $\theta_{0}=0.22$ led to the marginal probabilities of prevalence $\mathrm{Pr}(g_{i}=1)\approx 0.13$ and $\mathrm{Pr}(g_{i}=1)\approx 0.26$, with the higher prevalence resembling that estimated for the CRC EHR application (Section 6). Moreover, we compared the conditions when each individual receives a test at baseline ($\mathrm{Pr}(r_{i}=1)=1$), again similar to the CRC EHR application (Table 1), to the other extreme when nobody receives a baseline test ($\mathrm{Pr}(r_{i}=1)=0$). The latter setting was expected to be more challenging in estimation as it led to fully latent prevalence status (i.e., $g_{i}$ missing for all i). Finally, we also expected the estimator performance to depend on the degree of information that is available on the true test sensitivity. In particular, we were interested whether BayesPIM is able to estimate the test sensitivity κ when there is no prior information available on the test sensitivity. The latter is an extreme scenario, since, in cancer screening, there is usually some information available on test sensitivity. Hence, we compared a fully uninformative prior π(κ|α)=Beta(1,1) which is equivalent to a uniform (0,1) prior, an informative prior π(κ|α)=Beta(κ|α) where α was chosen such that the prior distribution was centered at the true sensitivity with a standard deviation of 0.05, and a point‐prior fixed at the true sensitivity (i.e., κ is treated as known). By a full factorial design we thus obtained 2×2×2×2×3=48 simulation conditions.

We generated 200 Monte Carlo data sets for each of the 48 conditions and ran, on each data set, the BayesPIM Gibbs sampler ((10), (11), (12), (13), (14)) until convergence using four randomly initialized MCMC chains. Convergence was assumed to be given when the Gelman‐Rubin convergence statistic $\hat{R}$ was below 1.1 for all model parameters and the effective sample size was at least 40 for each parameter (advice by Gelman et al. [26, p. 287]), while discarding half of all posterior draws as burn‐in (warm‐up). We evaluated convergence every 20,000 draws, combining draws across chains. The MCMC sampler was interrupted if convergence was not achieved after $5\times 10^{5}$ iterations per chain and non‐converged runs were replaced by additional converged runs. An analysis of the non‐converged runs is presented in the results section.

### 5.2 Results From Simulation 1

Simulation 1 was run on a 2024 state‐of‐the‐art Windows‐based computing cloud using 64 CPUs in parallel. Convergence of the Gibbs sampler was achieved, on average over all conditions, in 31.7 m (minimum = 5.9 m, maximum = 362.8 m), requiring in most cases fewer than $2\times 10^{5}$ iterations (see Figure B1). In total, the simulation workload consumed approximately 211 CPU‐days of aggregate compute time, executed in roughly four days of wall‐clock time by the parallel worker pool. The time until convergence (computing time) varied substantially across conditions (boxplots of all computing times are given in Figure B2). Fastest convergence, with 9.4 m on average (minimum = 8.5 m, maximum = 12.9 m), was achieved in the condition with smaller sample size ($n_{sim}=1000$), higher sensitivity (κ=0.8), lower prevalence ($\mathrm{Pr}(g_{i}=1)=0.13$), baseline tests, and a point prior on the true test sensitivity. Slowest convergence, with 100.1 m on average (minimum = 28.7, maximum = 362.8 m), was achieved in the condition with larger sample size ($n_{sim}=2000$), lower sensitivity (κ=0.4), higher prevalence ($\mathrm{Pr}(g_{i}=1)=0.26$), no baseline tests, and an uninformative prior on κ. Using a linear regression model with all experimental factors entered as main effects, we examined how these factors influenced average computing time (Table B1). The strongest effect was found for sensitivity: high sensitivity (κ=0.8) versus low sensitivity (κ=0.4) reduced computing time on average by 26.9 m. Computing time also increased with sample size, as expected, with $n_{sim}=2000$ requiring on average 17.0 m longer to converge than $n_{sim}=1000$. Point prior information on κ reduced computing time on average by 19.4 m compared with an uninformative prior. Higher true prevalence (e.g., $\mathrm{Pr}(g_{i}=1)=0.26$) and the absence of baseline tests ($\mathrm{Pr}(r_{i}=1)=0$) each increased computing time by about 9 m on average. We generally note that the absolute computation times reported depend on the specific hardware used and may deviate on other systems.

All results are based on converged runs only where non‐converged runs were replaced by additional converged runs. Non‐convergence until $5\times 10^{5}$ Gibbs iterations was rare but occurred in the specific condition with an uninformative prior on κ, high prevalence ($\mathrm{Pr}(g_{i}=1)=0.26$), and no baseline testing ($\mathrm{Pr}(r_{i}=1)=0$), which is the setting when model identifiability is weakest (see Figure B.3). Visual inspection of MCMC chains for exemplary datasets revealed that multi‐modality of the posterior distribution was the primary cause of non‐convergence; for an example see Figure B.4. Crucially, non‐convergence did not occur when an informative or point prior on κ (known κ) was used instead of an uninformative prior implying that information on κ regularizes an otherwise problematic likelihood surface.

**FIGURE 2:**
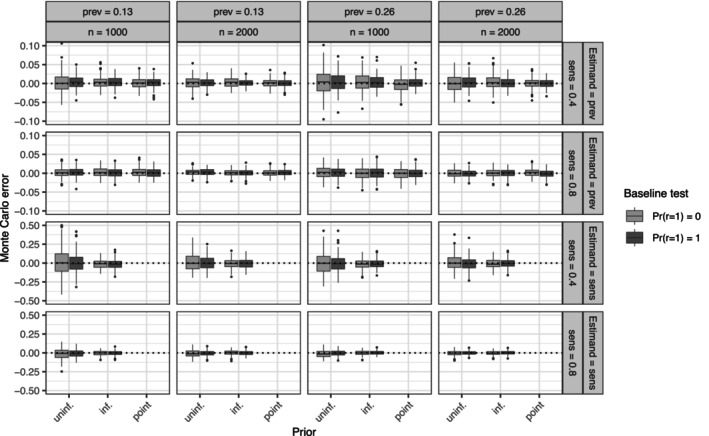
Monte Carlo (estimation) errors of the estimands: marginal prevalence probability ($\mathrm{Pr}(g_{i}=1)$, denoted “prev”) and the test sensitivity (κ, denoted “sens”). The first two rows give errors for the prevalence and the second two rows for the sensitivity. The priors on the test sensitivity κ are either uninformative (uninf.), informative (inf.) or fixed at the true value (point).

We evaluated the estimation error (Monte Carlo error) of all parameter estimates, defined as $\hat{\psi }-\psi$, where $\hat{\psi }$ is the posterior median estimate and ψ is the true value (Figures B.5–B.8). All parameters of the incidence and prevalence models (4) and (5) were approximately unbiased under all conditions. Parameters in the incidence model were estimated with greater precision than those in the prevalence model. Lower true test sensitivity (0.4 vs. 0.8), absence of a baseline test ($\mathrm{Pr}(r_{i}=1)=0$ vs. $\mathrm{Pr}(r_{i}=1)=1$), and higher baseline prevalence probability (0.26 vs. 0.13) increased estimator variance for all parameters. Variance decreased with larger sample sizes ($n_{sim}=2000$ vs. $n_{sim}=1000$) and with the use of informative or point priors on κ. These findings are illustrated by the Monte Carlo error of the marginal baseline prevalence probability $\mathrm{Pr}(g_{i}=1)$ (Figure 2). Furthermore, posterior median estimates of the test sensitivity κ are also shown and were approximately unbiased. Estimation was reliable for κ=0.8, even with uninformative priors, but variance was high for κ=0.4 when uninformative priors were used. In this case, informative priors decreased variance. Figure B.12 shows the approximate frequentist coverage probabilities of the 95% posterior credible intervals for all parameters. Coverage probabilities closely matched the nominal level across all conditions for all parameters, including test sensitivity.

**FIGURE 3:**
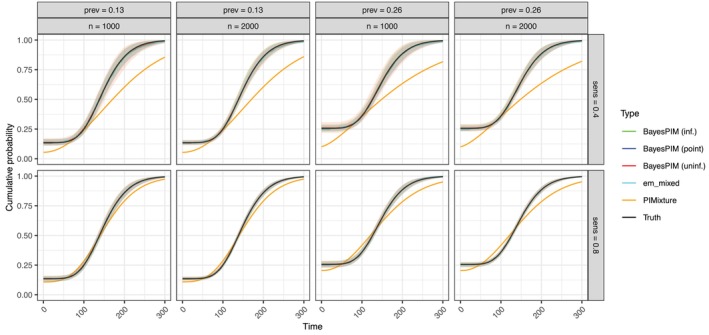
Posterior median estimates of the marginal mixture CIF $F_{t^{*}}(t|\beta ,\sigma ,\theta )$, point‐wise averaged over 200 Monte Carlo simulation runs with 95% quantiles shown as shaded regions. The condition $\mathrm{Pr}(r_{i}=1)=1$ is shown (for $\mathrm{Pr}(r_{i}=1)=0$ see Figure B.9). For em_mixed, κ was set to its true value. The lines of all models except PIMixture are overlapping.

To further compare model performance across conditions, we utilized posterior predictive mixture CIFs (Section 4.3), which depend jointly on all model parameters and thus serve as summary statistics (Figure 3 illustrates results for $\mathrm{Pr}(r_{i}=1)=1$; for $\mathrm{Pr}(r_{i}=1)=0$, see Figure B.9). These CIFs visualize the prevalence probability ($\mathrm{Pr}(g_{i}=1)$) as a point probability at time zero (i.e., a jump of size $\mathrm{Pr}(g_{i}=1)$ at t=0). They enable comparisons between BayesPIM, PIMixture, and the adapted non‐parametric CIF estimator em_mixed (Section 4.4). For PIMixture, we (correctly) specified a Weibull incidence model, included all covariates as predictors of $t_{i}$ and $g_{i}$, with logistic regression for the prevalence model. The CIF estimates from BayesPIM were approximately unbiased, consistent with the results for the individual model parameters discussed earlier. Estimator variance was slightly higher when an uninformative prior was used and true κ=0.4, compared to κ=0.8. Notably, the considerable uncertainty in κ estimation for κ=0.4 (Figure 2) did not result in significantly elevated uncertainty in CIF estimation, suggesting that posterior median CIF estimates are robust to posterior uncertainty in test sensitivity. In contrast, PIMixture, which assumes κ=1, produced biased CIF estimates, with greater bias observed when true κ=0.4 due to the larger deviation from the assumption of perfect sensitivity. The non‐parametric estimator em_mixed yielded approximately unbiased estimates, provided that sensitivity κ was correctly specified. However, em_mixed performed poorly, as expected (cf. Section 4.4), when $\mathrm{Pr}(r_{i}=1)=0$ (Figure B.9). In particular, baseline prevalence estimates were then biased. Additional simulations showed that this bias decreased as more baseline tests became available and was negligible for a moderate proportion of tests done (e.g., $\mathrm{Pr}(r_{i}=1)=0.5$; Figure B.13).

### 5.3 Set‐Up of Simulation 2

To evaluate BayesPIM in a realistic setting we conducted a Monte Carlo simulation in which we generated data that strongly resembled the CRC EHR (Simulation 2). The data are described in Section 2 and the results of the analyzes with BayesPIM are presented in Section 6. To obtain covariate and screening times distributed similarly to those observed in the real‐world CRC EHR, we devised a resampling procedure, described in detail in Section C.

For each simulated data set, we generated $n_{sim}$ new individuals with ($g_{k},r_{k},t_{k},v_{k},x_{k}$), $k=1,\ldots ,n_{sim}$. For each k, we randomly selected one individual $i^{\prime }$ with replacement from the CRC EHR and used his/her gender and age ($x_{i^{\prime }}$) for the k‐th newly generated individual (i.e., $x_{k}:=x_{i^{\prime }}$). Subsequently, we generated $t_{k}$ and $g_{k}$ according to models (4) and (5), entering both covariates in both models. The true model parameters θ, β and σ were fixed at the posterior median estimates from the application study presented in Section 6 (Table 4). The transition time distribution was chosen as Weibull, which had best fit in the application study. The probability of a baseline test was set to its estimate of $\mathrm{Pr}(r_{i}=1)=0.93$ in the CRC EHR (Table 1).

To obtain new screening times and run screening tests according to the hierarchical model (Section 3.2), Equations (2) and (3), we used the observed screening times $v_{i^{\prime }}=(v_{i^{\prime }1},v_{i^{\prime }2},\ldots ,v_{i^{\prime }c_{i^{\prime }}})$ of the sampled unit $i^{\prime }$ as donor for new screening times. Specifically, we set $v_{k1}=0$ and stopped screening at baseline with probability κ, that is, $y_{k1}=1$ (positive baseline test), only if $g_{k}=1$ and $r_{k}=1$. If $r_{k}=0$, $y_{k1}$ was missing, as described in Section 3.2. Else we set $y_{k1}=0$ and, subsequently, for each $j=2,\ldots c_{i^{\prime }}$ we followed the following steps:

1. Set $v_{kj}:=v_{i^{\prime }j}$.
2. Generate $y_{kj}$ as defined by (3).

The procedure was stopped at an occasion $j^{\prime }$ if $y_{kj^{\prime }}=1$ such that $v_{k}=(v_{k1},\ldots ,v_{kc_{k}})$, with $c_{k}=j^{\prime }$ the event occasion. Additional steps were taken to simulate a right censoring process that approximated that of the CRC EHR; for details, see Section C. As a check, Figure C.2 compares the sampling distributions of various statistics calculated on repeated samples of the screening times $\left(v_{1},\ldots ,v_{n_{sim}}\right)$ to the observed statistics in the CRC EHR (e.g., mean and standard deviation of the event/censoring time; mean number of test occasions). Results suggested that the generated and observed screening times were similar, which indicated that we were successful in generating screening data similar to the real‐world EHR.

We simulated 200 data sets per condition and applied BayesPIM, PIMixture and em_mixed, as in Simulation 1. Specifically, we considered 3×2×2=12 simulation conditions comparing different sample sizes ($n_{sim}=$ 810, 1620 or 3240), low and high test sensitivity (κ= 0.4 or 0.8), and two different right censoring processes (observed as in the CRC EHR or extended by 10 years). The condition with $n_{sim}=810$, κ=0.8 was similar to the real CRC EHR and compared to conditions with larger sample sizes ($n_{sim}=1620$ and $n_{sim}=3240$) and lower test sensitivity (κ=0.4), respectively. In addition, we added a condition with an extended right censoring process where the time of right censoring was simulated to occur ten years later than in the CRC EHR; see Section C.2. This setting was added to assess model performance if follow‐up had been longer than that observed in the real CRC EHR.

#### 5.3.1 Results from Simulation 2

With informative or point (fixed) priors on the test sensitivity, the Gibbs sampler reliably converged within $5\times 10^{5}$ draws (see Figures D.1 and D.2). However, with uninformative priors, non‐convergence was observed in the condition $n_{sim}=810$, κ=0.4, and observed right censoring. The impact of uninformative priors was also particularly evident in explaining variations in Monte Carlo (estimation) errors across conditions. Pronounced bias was observed in the intercepts $\beta_{0}$ and $\theta_{0}$ of the incidence (4) and prevalence models (5) under the κ=0.4 condition (Figures D.3 and D.4). This bias diminished with larger sample sizes, higher test sensitivity (κ=0.8), or extended follow‐up. With an informative prior (centered at the true κ) this bias was strongly decreased and bias vanished when a point prior (fixed at the true κ) was employed. In these settings, estimation was also more efficient compared to the uninformative prior case. In the κ=0.8 condition, bias was approximately zero when informative and point priors were put on the test sensitivity, and it was small when the uninformative prior was used.

We also studied the frequentist coverage probability of the 95% posterior intervals for all parameters (Figure D.7), demonstrating that coverage was approximately nominal if the intercept and κ parameters did not suffer substantial bias which was the case when informative or point priors were used.

**FIGURE 4:**
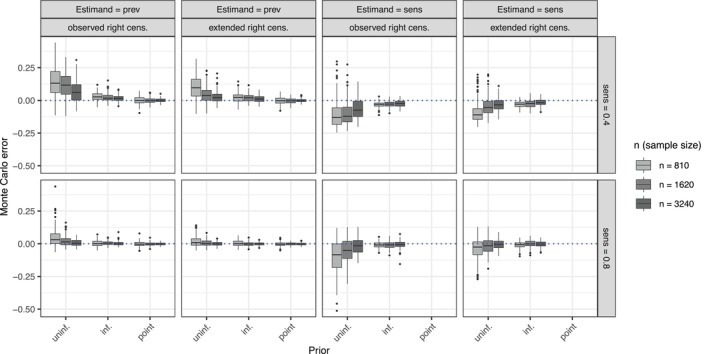
Monte Carlo (estimation) error of the marginal prevalence probability ($\mathrm{Pr}(g_{i}=1)$, denoted “prev”) and the test sensitivity (κ, denoted “sens”) estimands. The first two columns give errors for the prevalence and the second two columns for the sensitivity. The priors on the test sensitivity κ are either uninformative (uninf.), informative (inf.) or fixed at the true value (point).

These results are summarized in Figure 4, which shows the Monte Carlo (estimation) errors, as defined in Simulation 1, for the marginal prevalence probability $\mathrm{Pr}(g_{i}=1)$ which depends on the prevalence model parameters θ, and the test sensitivity κ. In the uninformative prior setting for κ, the estimation of κ was inaccurate in both the κ=0.4 and κ=0.8 conditions, reflecting results on the model parameter estimates described above. Although estimation appeared consistent, meaning that bias decreased with larger sample sizes or extended follow‐up, substantially larger samples (beyond $n_{sim}=3240$) or longer follow‐up would have been required to eliminate the bias in the κ estimates. With informative prior, bias on the κ estimates was small although still visible in the κ=0.4 condition and absent in the κ=0.8 condition, translating to smaller bias on the parameter estimates and prevalence estimates. This residual bias was fully eliminated with correctly specified point priors (fixed) κ. In summary, these results suggest that estimating test sensitivity was challenging given the CRC EHR screening time process, especially without prior information and in the κ=0.4 setting.

**FIGURE 5:**
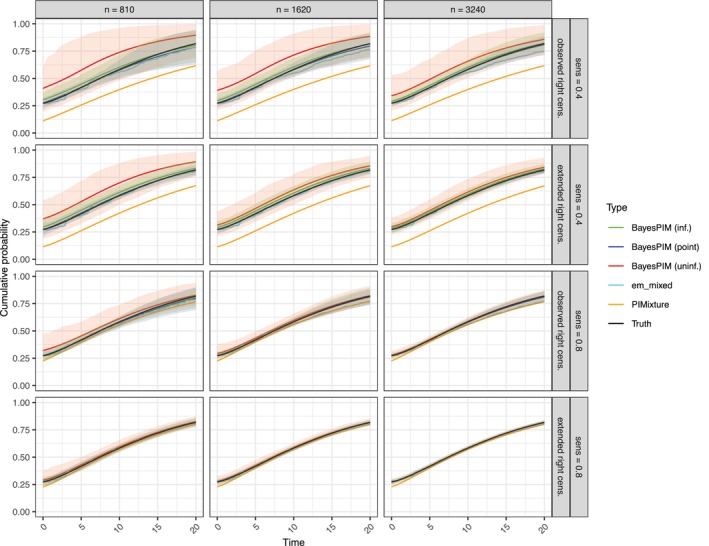
Marginal mixture CIFs, $F_{t^{*}}(t|\beta ,\sigma ,\theta )$, point‐wise averaged over 200 Monte Carlo simulation runs with 95% quantiles shown as shaded regions (for clarity these bounds have been omitted for PIMixture and em_mixed). For BayesPIM the posterior median of the posterior predictive marginal mixture CIF is shown. For PIMixture and em_mixed the corresponding maximum likelihood estimates are shown. Lines of BayesPIM (point) and “Truth” are overlapping in most graphs.

A key question was how the Monte Carlo errors affected the estimates of the marginal mixture CIFs, $F_{t^{*}}(t|\beta ,\sigma ,\theta )$ (Figure 5; for marginal CIFs of the non‐prevalent population, $F_{t}(t|g=0,\beta ,\sigma )$, see Figure D.5). As in Simulation 1, these CIFs facilitated comparisons with PIMixture and em_mixed. When BayesPIM used an uninformative κ prior, the resulting bias was evident in the CIFs, though the bias was smaller in the κ=0.8 conditions. In contrast, BayesPIM with κ fixed at its true value (point prior) provided the most precise estimates, even in the most challenging condition ($n_{sim}=810$, κ=0.4). The non‐parametric em_mixed estimator was generally accurate but exhibited some bias beyond ten years when κ=0.4. This bias disappeared with longer follow‐up (extended right censoring). Given that most cases in the observed right‐censoring process were censored after approximately ten years (Figure C.1), it is evident that the non‐parametric estimator lacked sufficient data to produce accurate incidence estimates beyond this time. This limitation also resulted in larger variance, which is not shown in Figure 5 but can be seen in Figure D.6. In contrast, BayesPIM leveraged model‐based extrapolation, providing better estimates in these sparse‐data settings. For the comparison model PIMixture, the results were consistent with Simulation 1. Specifically, under κ=0.4, PIMixture exhibited greater bias compared to κ=0.8, due to its stronger deviation from the assumption of perfect sensitivity.

## 6 Application to the Dutch CRC EHR

We applied BayesPIM, PIMixture, and em_mixed to the CRC EHR introduced in Section 2. In doing so, we specified BayesPIM models with Weibull, log‐logistical, log‐normal, and exponential distributed transition times $t_{i}$ (model (4)). To obtain the exponential model, we used a Weibull specification and constrained σ=1. This model is equivalent to a Markov model due to its constant hazard property and it is primarily included for comparison with the less restrictive models. We included subjects' gender and age as covariates in both the incidence and the prevalence models (models (4) and (5)). We used priors as specified in Section 3.4. For the sensitivity κ, we compared an uninformative prior that sets α=(1,1) to an informative prior that we based on a range of test sensitivities published in the literature (Section 1). Specifically, test sensitivities for colonoscopy detection of adenomas range between 0.65 and 0.92 [9]. We therefore centered the Beta prior for κ at 0.8 with standard error 0.05; using numerical root‐finding we determined α=(50.4,12.6), such that Pr(κ∈(0.696,0.890))≈0.95. In addition, we added the setting of perfect test sensitivity by constraining κ=1.

We ran Gibbs sampler steps ((10), (11), (12), (13), (14)) until convergence, where step (14) is omitted in the setting that constrains κ to one. Convergence was assumed when the upper confidence bound of the Gelman‐Rubin convergence diagnostic $\hat{R}$ calculated over the most recent half of all iterations first came under the value of 1.1 for all model parameters and the effective sample size of all MCMC samples was at least $10^{3}$ for each parameter. We ran four randomly initialized MCMC chains in parallel and assessed convergence every $5\times 10^{4}$ draws.

WAIC slightly favored a Weibull model over the alternative distributions (Table 3). Furthermore, an informative prior gave slightly better WAIC than an uninformative prior. The models with perfect sensitivity (κ=1) had worse fit than the models allowing κ<1, indicating that relaxing the assumption of perfect sensitivity made by PIMixture improved model fit.

**TABLE 3:** Model WAIC and number of draws per chain ($\times 10^{5}$) after convergence. The prior for the test sensitivity κ is either chosen informative (Inf.), uninformative (Uninf.) or κ is constrained to one (κ=1).

Model	WAIC			Draws per chain ($\times 10^{5}$)		
	Inf.	Uninf.	κ=1	Inf.	Uninf.	κ=1
Exponential	1598.0	1600.4	1599.5	1.5	2.0	1.0
Weibull	1595.4	1598.4	1597.0	4.0	6.0	2.5
Log‐logistic	1596.1	1599.7	1599.1	2.0	4.0	1.0
Log‐normal	1596.8	1600.2	1601.0	2.5	5.5	1.5

Estimates for the test sensitivity κ differed (0.79 versus 0.70) between informative and uninformative priors but the uninformative prior model had a wide credible interval ([0.44, 0.94]), suggesting that κ was only weakly identified in this data set. This conclusion is supported by the fact that the credible interval of κ in the model with informative κ prior had similar length ([0.69, 0.88]) as the 2.5 to 97.5 quantile range of the Beta prior specified for κ; see above. However, the added certainty on κ encoded in the informative Beta prior decreased the credible interval lengths of all other model parameters as compared to the model without informative κ prior. Having said this, the regression coefficient estimates were similar across models, including the exponential model, suggesting robustness of inference on risk factors to the model assumptions on κ and the distribution of $t_{i}$.

The sign and size of the coefficient estimates (Table 4) suggested that older age increased incidence probability of an adenoma, while gender did not have a clear effect on incidence. Specifically, individuals who had an age of one standard deviation (7.71 years) above the sample mean of 52.9 years at baseline were expected to have transition time to an adenoma reduced by the factor exp(−0.175)=0.839 (16.1% faster transition). Both gender and age were predictors of prevalence status at baseline, with female gender decreasing and older age increasing the probability of prevalence. These estimates are in line with epidemiological expectations suggesting that older subjects and men are at a higher risk of developing adenomas and CRC.

**TABLE 4:** Posterior median estimates with 95% credible interval for the coefficients from the Weibull and exponential BayesPIM models for different prior settings on the test sensitivity κ.

	Weibull (Inf.)	Weibull (Uninf.)	Exponential (Inf.)
*Model for* $t_{i}$:			
$\beta_{0}$	2.79 [2.50, 3.21]	2.78 [2.43, 3.31]	3.07 [2.77, 3.42]
Female	−0.11 [−0.44, 0.20]	−0.13 [−0.61, 0.22]	−0.11 [−0.52, 0.27]
Age^a^	−0.17 [−0.32, −0.03]	−0.16 [−0.33, 0.04]	−0.17 [−0.36, 0.02]
σ	0.74 [0.55, 1.02]	0.72 [0.48, 1.04]	1.00 [1.00, 1.00]
*Model for* $g_{i}$:			
$\theta_{0}$	−0.51 [−0.69, −0.31]	−0.41 [−0.70, 0.09]	−0.52 [−0.70, −0.32]
Female	−0.24 [−0.46, −0.02]	−0.25 [−0.49, −0.01]	−0.24 [−0.46, −0.02]
Age^a^	0.33 [0.21, 0.45]	0.34 [0.21, 0.49]	0.34 [0.22, 0.46]
*Sensitivity*:			
κ	0.79 [0.69, 0.88]	0.70 [0.44, 0.94]	0.79 [0.69, 0.88]

^a^ Age was z‐standardized (before standardization: mean = 52.9 years with SD = 7.71 years).

**FIGURE 6:**
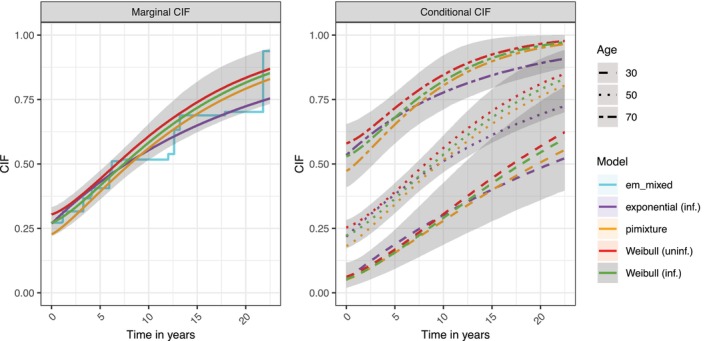
Marginal and conditional mixture cumulative incidence functions (CIF), $F_{t^{*}}(t|\beta ,\sigma ,\theta )$ and the conditional CIF, $F_{t^{*}}(t|\tilde{x},\beta ,\sigma ,\theta )$, for Weibull BayesPIM with uninformative (uninf.) prior and informative (inf.) prior. For comparison, corresponding CIFs from the Weibull PIMixture model, the exponential BayesPIM model (inf.), and em_mixed are given. The lines represent posterior median estimates and the shaded regions indicate the 95% credible intervals of the Weibull (inf.) models (for overlayed intervals from the Weibull (uninf.) model see Figure E.1).

Figure 6 gives the marginal mixture CIF, $F_{t^{*}}(t|\beta ,\sigma ,\theta )$, and the conditional CIF, $F_{t^{*}}(t|\tilde{x},\beta ,\sigma ,\theta )$, for different age groups (i.e., $\tilde{x}$ was age set to 30, 50 and 70). The Weibull and the exponential models are shown, respectively. The corresponding CIFs obtained from a fit by PIMixture are added. For this, we used a PIMixture model that assumes the same Weibull incidence model (4) as BayesPIM. Furthermore, we added the non‐parametric estimator em_mixed of $F_{t^{*}}(t|\beta ,\sigma ,\theta )$ (see Section 4.4) which allows a visual inspection of goodness of fit of the marginal CIF. We set the sensitivity κ to 0.80 in em_mixed. See Figure E.2 for an equivalent plot for the non‐prevalent population.

The mixture CIFs depict the sum of prevalence and follow‐up incidence probability until a given point in time. The prevalence probability can be read at the intercept and is interpreted as the proportion of the population that has an adenoma at the point of inclusion in the surveillance program ($\mathrm{Pr}(g_{i}=1)$). Specifically, the models with informative and uninformative prior estimated this proportion at 0.274 [0.222, 0.333] and 0.307 [0.217, 0.475], respectively; the exponential (inf.) model at 0.269 [0.219, 0.330]. Again we note the wider credible interval of the Weibull (uninf.) model as a consequence of the uninformative κ prior. In addition, the prevalence estimate was higher, relating to the fact the Weibull (uninf.) model estimated κ lower (Table 4). The observed prevalence was 0.204 (Table 1), suggesting that an estimated 34% (0.07 points) of prevalence was unobserved (latent). The non‐parametric estimator em_mixed estimated the prevalence probability at 0.271, which is very close to the Weibull (inf.) and exponential model estimates and reassured us about model fit of the best model according to WAIC. To the contrary, PIMixture estimated the prevalence lower at 0.228, owing to the assumption of κ=1. The PIMixture prevalence estimate was similar to that of the BayesPIM model that assumed κ=1 (0.217; not shown in Figure 6).

While there were pronounced differences in prevalence estimates between models, the incidence model estimates were similar (Table 4). The marginal CIF of Weibull (inf.), Weibull (uninf.), and PIMixture had an almost parallel trajectory (Figure 6, left side). The marginal CIF of the exponential model had different curvature, however, owing to its constraint of σ=1, which led in particular in the time after 10 years to different predicted probabilities as compared to the Weibull models. It is important to note that, in this data set, most censoring occurs before the time of 10 years, and the last adenoma event occurred at 22.5 years (Table 1). Hence, the uncertainty of the estimates increases after 10 years, as indicated by wider credible interval bounds shown for the Weibull (inf.) model. The non‐parametric fit of em_mixed can be used as a visual inspection of incidence model fit. In this case, the Weibull (inf.) which had lowest WAIC indeed had similar fit to em_mixed, but the exponential model fit similarly well in this visual inspection. Both models fit notably better to em_mixed than the PIMixture model.

The right panel of Figure 6 shows the conditional mixture CIF, $F_{t^{*}}(t|\tilde{x},\beta ,\sigma ,\theta )$, for selected age groups. The higher risk of prevalence for older individuals was reflected by the increasing ordinate intercepts across groups. Furthermore, in the 30 and 70 years groups which were further from the sample mean of 52.9 years than the 50 years group, some pronounced differences between models were found. This suggests that model selection, in this case, was more important for the estimation of conditional risk than for estimation of marginal risk.

## 7 Discussion

We introduced BayesPIM, a modeling framework for estimating time‐ and covariate‐dependent incidence probabilities from screening and surveillance data. The model is used when the disease may be prevalent at baseline, tests at baseline are fully or partially missing, and the test for disease has imperfect sensitivity. In this regard, we extended the available PIM PIMixture, which assumes perfect sensitivity [1, 2]. We applied the model to data from high familial risk CRC EHR surveillance by colonoscopy, demonstrating that conditioning incidence and prevalence estimates on covariates explains substantial heterogeneity in adenoma risk (cf. Figure 6). These estimates can serve as valuable input for targeted screening strategies, such as higher intensity for older individuals with familial CRC risk and recognizing that older individuals have a high probability of having adenomas at baseline. More generally, BayesPIM is readily applicable to other screening data that often have a similar structure (Section 2). In our study, we assumed perfect specificity, which was a plausible assumption for the CRC EHR because colonoscopy findings are validated by pathology; similar confirmatory tests are common in other screening programs.

In estimation, we leveraged a Bayesian Metropolis‐within‐Gibbs sampler with data augmentation for the latent transition times and the latent prevalence status. Our Bayesian approach facilitates the inclusion of prior information on the model parameters. We have found that weakly informative priors on the incidence and prevalence model parameters (cf. Section 3.4) provide effective regularization without biasing the parameter estimates. Arguably, the prior information on β, model (4), and θ, model (5), is scarce in practice, making a weakly informative prior choice an attractive default. Furthermore, we studied the role of the prior on the test sensitivity κ in substantial detail. When a Beta prior is used, updating κ is conjugate (see (14)) and hence fast. However, through simulations we demonstrated that a fully uninformative prior, chosen uniform (0,1), can lead to estimation problems, such as non‐convergence, multi‐modality of the posterior, and bias in κ and other model parameters. Adding some information on κ through a centered prior on the most likely value yielded reliably converging Markov chains and approximately zero bias in the BayesPIM parameter estimates. This included more challenging settings than the one actually observed in the CRC EHR, such as low sensitivity κ=0.4 or the absence of a baseline test ($\mathrm{Pr}(r_{i}=1)=0$). We, therefore, advise the use of informative priors in practice, which is facilitated by the fact that test sensitivity is usually roughly known in cancer screening, as demonstrated for the CRC EHR (Section 6). Alternatively, several analyses may be run with a range of fixed κ values, and the model with the highest WAIC given preference. Clearly, if κ is known with high certainty, fixing κ at this value is the best option. These measures are particularly important in small samples or when there is only short follow‐up, as in the CRC EHR (Table 1, Figure C.1).

A natural question is how sensitive our modeling framework is to misspecification of the prior for the test sensitivity κ. In general, the impact on parameter estimates and CIFs will depend both on the discrepancy between the prior assumptions and the true value of κ and on how informative the data are about κ. When the data are highly informative, the likelihood will pull the posterior away from a misspecified prior, whereas when information on κ is weak, inferences will inherit more of the prior assumptions. An extreme instance of misspecification occurs for PIMixture, which corresponds to fixing κ=1 and thus assuming perfect sensitivity a priori. In Simulations 1 and 2 we observed that, when the true sensitivity was high (κ=0.8), the resulting bias in CIF estimates was moderate, whereas for lower sensitivity (κ=0.4) the bias became substantial. In practice, we therefore recommend routinely performing sensitivity analyses over a plausible range of fixed κ values (or alternative priors on κ) to assess the impact of potential misspecification of test sensitivity on the substantive conclusions.

Model fit evaluation is an important aspect of modeling with BayesPIM, and we proposed the WAIC as a means to select the best transition times ($t_{i}$) distribution. In the application, WAIC only slightly favored a Weibull model over the alternative models (log‐normal, log‐logistic, and exponential). In addition, we adapted the non‐parametric estimator em_mixed, initially proposed by Witte et al. [12] for screening data without baseline prevalence but imperfect test sensitivity, to our setting with prevalence. In simulations, em_mixed had excellent performance, provided baseline tests were available for a moderate to high proportion of the sample. Hence, the estimator serves as an additional fit assessment in these settings. In the application, the visual comparison of the em_mixed CIF to that of BayesPIM was indeed reassuring on model fit. In future work, em_mixed might be extended to work well in settings with only a few or no baseline tests. However, while useful for marginal assessment of fit, em_mixed cannot replace BayesPIM which allows relating incidence and prevalence to covariates to identify risk factors, make personalized predictions, and estimate conditional CIFs (see Figure 6). Furthermore, as a parametric model BayesPIM can be used to extrapolate CIFs beyond the observed screening times distribution (e.g., maximum censoring time), which is not feasible with non‐parametric estimators.

A strong aspect of our study of the CRC EHR is that we assessed the performance of BayesPIM under the observed screening times and right censoring process through simulations (Section 5.3). Our results indicated that BayesPIM can reliably estimate the model parameters under that condition ($n_{sim}=810$, κ=0.8, observed right censoring), and also provided good performance in the more challenging setting with κ=0.4, provided that an informative κ prior was used. Here, we found a small bias in the κ and prevalence estimates, unless κ was set to its true value through a point prior. This finding illustrates the importance of evaluating estimation performance in real‐world settings as a supplemental analysis to the data analysis. To this end, Sections C.1 and C.2 explain how to generate screening times from observed EHR screening data. This approach should be adopted in future work.

Based on our results, we recommend using BayesPIM in settings with imperfect test sensitivity, whereas in settings where perfect sensitivity can be assumed, PIMixture remains an appropriate choice. Although BayesPIM is computationally more demanding than PIMixture, Simulation 1 (Section 5) showed that convergence was typically achieved within minutes to a few hours, depending on the experimental condition. With contemporary hardware, such runtimes are compatible with routine use, as model fitting is typically performed offline and on single data sets.

Limitations of BayesPIM currently include the reliance on Assumptions (a)–(e), Section 3.2, as well as our focus on two‐parameter survival distributions. The conditional independence of prevalence‐incidence components (a) and missing at random baseline test outcomes (b), appear necessary, albeit realistic identifying assumptions. Although also the assumption of uninformative scheduling of screening (c) was plausible for the CRC EHR, because most adenomas are not symptomatic, the assumption may need to be relaxed in future research for settings where symptomatic pre‐state diseases or diseases let patients initiate a screening test. A good starting point seems to be a parametric modeling assumption for the association between the scheduling times $v_{ij}$ and $\left(g_{i},t_{i}\right)$. Furthermore, the assumption of uninformative censoring (d) is a well‐known standard assumption in survival modeling which may be violated, for example, in settings with right censoring due to competing events such as death caused by the disease. In this regard, BayesPIM could be extended to competing event modeling similar to the Hyun et al. [8] extension for the PIMixture model. Assumption (e) concerns the stability of test sensitivity across time. This assumption may be violated if adenomas become easier to detect over time (e.g., due to bleeding or increase in volume) which may affect the probability of discovery in screening. Letting κ change over time seems to be a viable path for further research. Extending available multi‐state semi‐Markov models for progressive diseases [20] for prevalence and imperfect sensitivity is also a viable option. In addition, extending BayesPIM to account for recurring adenomas after the first adenoma will be needed for modeling adenoma occurrence in some high‐risk populations (e.g., patients with the so‐called Lynch syndrome develop adenomas frequently). Given BayesPIM's focus on two‐parameter distributions, estimates may be biased if transition times strongly deviate from the assumed distribution. Semi‐parametric extensions (e.g., Cox‐type transitions) may be considered in the future to make BayesPIM more flexible, but they may require large samples.

In conclusion, BayesPIM brings a flexible, Bayesian approach to handling latent prevalence and imperfect test sensitivity in screening and surveillance data. By incorporating informative priors, assessing model fit through both parametric and non‐parametric approaches, and demonstrating robust performance under realistic conditions, BayesPIM enables more accurate, data‐driven, and ultimately patient‐centered screening strategies.

## Funding

The authors have nothing to report.

## Conflicts of Interest

The authors declare no conflicts of interest.

## Supporting information




**Data S1:** A pdf with following sections: Section A: Proofs and technical details. Section B: Additional results from Simulation 1. Section C: Additional details on the set‐up of Simulation 2. Section D: Additional results from Simulation 2. Section E: Additional results from the CRC application.

**Rcodes:** A zip‐archive with R code, in particular: A tar.gz file with the BayesPIM package (see readme∖_packages). A tar.gz file with the EMmixed package (see readme∖_packages). A zip‐archive with R code and additional documentation (see readme∖_simulations) for running and analyzing the simulation studies.

## Data Availability

The data that support the findings from the application to the high‐risk CRC screening data are available on request from the FCRC study authors and participating medical centers (see Acknowledgments). The data are not publicly available due to privacy or ethical restrictions. The data that support the findings from the simulation study (simulation output files) are provided for download (link provided in the Supporting Information).
