# Supplementary material for: A Bayesian Prevalence‐Incidence Mixture Model for Screening Outcomes With Misclassification
Source: Stat Med. 2026 Apr 7;45(8-9):e70433. doi: 10.1002/sim.70433 (PMC13054642; doi:10.1002/sim.70433)
Supplement: Supplementary file 1 — Data S1: A pdf with following sections: Section A: Proofs and technical details. Section B: Additional results from Simulation 1. Section C: Additional details on the set‐up of Simulation 2. Section D: Additional results from Simulation 2. Section E: Additional results from the CRC application. [file SIM-45-0-s001.pdf]

”A Bayesian prevalence-incidence mixture model for screening outcomes with misclassification”

Supplemental Material

by Thomas Klausch, Birgit Lissenberg-Witte & Veerle Coupé

Amsterdam University Medical Center, Department of Epidemiology and Data Science,
Amsterdam, The Netherlands

A Proofs

A.1 Observed-data likelihood

We derive the observed-data likelihood $\mathcal{L}(\boldsymbol{\beta}, \sigma, \boldsymbol{\theta}, \kappa \mid \boldsymbol{\mathcal{D}})$. For the following, recall the notation from Section 3.1 of the main paper. In particular, screening stops at the first positive test (event) or at right censoring. In the case of an event and baseline test ($r_i = 1$) we have $\mathbf{y}_i = (y_{i1}, y_{i2}, \ldots, y_{ic_i-1}, y_{ic_i}) = (0, 0, \ldots, 0, 1)^T$ with screening times $\mathbf{v}_i = (v_{i1}, v_{i2}, \ldots, v_{ic_i})^T$ and $v_{ic_i} < \infty$ (finite event time). In the case of right censoring we have $\mathbf{y}_i = (0, 0, \ldots, 0)^T$ with times $\mathbf{v}_i = (v_{i1}, v_{i2}, \ldots, v_{ic_i-1}, \infty)^T$ so that $v_{ic_i} = \infty$ and $y_{ic_i} = 0$ indicates right censoring. Hence, the observed vector of test outcomes is complete. Let $\mathbf{y}_{i,\text{com}}$ denote the complete-data vector of test outcomes, then $\mathbf{y}_{i,\text{com}} = \mathbf{y}_i$ if $r_i = 1$.

Without a baseline test ($r_i = 0$), we have y_{i1} is missing, so that the observed test outcomes are $\mathbf{y} = (y_{i2}, \ldots, y_{ic_i})$. Hence, the complete-data vector of outcomes is $\mathbf{y}_{i,\text{com}} = (y_{i1}, \mathbf{y})$ with y_{i1} unobserved (missing). We will discuss below how this missing test is handled under the model assumptions laid out in Section 3.2.

A.1.1 Likelihood factorization

The observed-data likelihood consists of two factors, one for the individuals with baseline test ($r_i = 1$) and one for the individuals without ($r_i = 0$). When there is no baseline test, the missing outcomes are integrated out of the likelihood as follows

$$\begin{aligned}\mathcal{L}(\boldsymbol{\beta}, \sigma, \boldsymbol{\theta}, \kappa \mid \boldsymbol{\mathcal{D}}) = &\left(\prod_{i:r_i=1} f_{r,\mathbf{v},\mathbf{y}_{\text{com}}}(r_i = 1, \mathbf{v}_i, \mathbf{y}_i \mid \mathbf{x}_i, \boldsymbol{\beta}, \sigma, \boldsymbol{\theta}, \kappa) \right) \\ &\times \left(\prod_{i:r_i=0} \sum_{k=0}^{1} f_{r,\mathbf{v},\mathbf{y}_{\text{com}}}(r_i = 0, \mathbf{v}_i, (y_{i1} = k, \mathbf{y}_i) \mid \mathbf{x}_i, \boldsymbol{\beta}, \sigma, \boldsymbol{\theta}, \kappa) \right)\end{aligned} \tag{S-1}$$

By the DGM defined in the hierarchical model in Section 3.2 and the DAG in Figure 1 we can derive:

$$\begin{aligned}f_{r,\mathbf{v},\mathbf{y}_{\text{com}}}(r_i, \mathbf{v}_i, \mathbf{y}_i \mid \mathbf{x}_i, \boldsymbol{\beta}, \sigma, \boldsymbol{\theta}, \kappa) &= \sum_{l=0}^{1} \int_0^\infty f_{g,t,r,\mathbf{v},\mathbf{y}_{\text{com}}}(g_i = l, t_i, r_i, \mathbf{v}_i, \mathbf{y}_i \mid \mathbf{x}_i, \boldsymbol{\beta}, \sigma, \boldsymbol{\theta}, \kappa) dt_i \\ &= \sum_{l=0}^{1} \int_0^\infty f_g(g_i = l \mid \mathbf{x}_i, \boldsymbol{\theta}) f_t(t_i \mid \mathbf{x}_i, \boldsymbol{\beta}, \sigma) f_r(r_i \mid \mathbf{x}_i) \\ &\times f_{\mathbf{v},\mathbf{y}_{\text{com}}}(\mathbf{v}_i, \mathbf{y}_i \mid g_i = l, r_i, t_i, \mathbf{x}_i, \kappa) dt_i \\ &\propto \sum_{l=0}^{1} f_g(g_i = l \mid \mathbf{x}_i, \boldsymbol{\theta}) \int_0^\infty f_t(t_i \mid \mathbf{x}_i, \boldsymbol{\beta}, \sigma) \\ &\times f_{\mathbf{v},\mathbf{y}_{\text{com}}}(\mathbf{v}_i, \mathbf{y}_i \mid g_i = l, r_i, t_i, \mathbf{x}_i, \kappa) dt_i \\ &= (1 - \Phi(\mu_i)) \int_0^\infty f_t(t_i \mid \mathbf{x}_i, \boldsymbol{\beta}, \sigma) f_{\mathbf{v},\mathbf{y}_{\text{com}}}(\mathbf{v}_i, \mathbf{y}_i \mid g_i = 0, r_i, t_i, \mathbf{x}_i, \kappa) dt_i \\ &+ \Phi(\mu_i) \int_0^\infty f_t(t_i \mid \mathbf{x}_i, \boldsymbol{\beta}, \sigma) f_{\mathbf{v},\mathbf{y}_{\text{com}}}(\mathbf{v}_i, \mathbf{y}_i \mid g_i = 1, r_i, t_i, \mathbf{x}_i, \kappa) dt_i\end{aligned} \tag{S-2}$$

We note that the term $f_r(r_i \mid \mathbf{x}_i)$ is dropped in the third line of (S-2), because it is does not involve parameters $(\boldsymbol{\beta}, \sigma, \boldsymbol{\theta}, \kappa)$. This is a consequence of the missing at random baseline test outcomes assumption (Little and Rubin, 2002) which will allow ignore missing baseline test

outcomes by integrating them out, as discussed below.

The density $f_{\mathbf{v},\mathbf{y}_{\text{com}}}(\mathbf{v}_i, \mathbf{y}_i \mid g_i, r_i, t_i, \mathbf{x}_i, \kappa)$ can be viewed as the likelihood contribution (given κ) of the test outcomes and screening schedule times conditional on known values for the prevalence status g_i and transition time t_i. These values are, however, unknown and, therefore, integrated out in (S-2). In the following, we derive likelihood $f_{\mathbf{v},\mathbf{y}_{\text{com}}}$ while distinguishing between individuals with ($r_i = 1$) and without ($r_i = 0$) baseline test and non-prevalent ($g_i = 0$) and prevalent individuals ($g_i = 1$).

A.1.2 Likelihood contribution of the test outcomes with baseline test ($r_i = 1$)

Regardless of prevalence status, we have for individuals with baseline test complete test outcomes ($\mathbf{y}_{i,\text{com}} = \mathbf{y}_i$):

$$
\begin{aligned}
f_{\mathbf{v},\mathbf{y}_{\text{com}}}(\mathbf{v}_i, \mathbf{y}_i \mid g_i, r_i = 1, t_i, \mathbf{x}_i, \kappa) &= \prod_{j=1}^{c_i} f_{v,y}(v_{ij}, y_{ij} \mid g_i, r_i = 1, t_i, \bar{\boldsymbol{v}}_{ij}, \mathbf{x}_i, \kappa) \\
&= \prod_{j=1}^{c_i} \sum_{m=0}^{1} f_{v,y,y^*}(v_{ij}, y_{ij}, y^*_{ij} = m \mid g_i, r_i = 1, t_i, \bar{\boldsymbol{v}}_{ij}, \mathbf{x}_i, \kappa) \\
&= \prod_{j=1}^{c_i} \sum_{m=0}^{1} \Pr(y_{ij} \mid v_{ij}, y^*_{ij} = m, \kappa) \Pr(y^*_{ij} = m \mid g_i, t_i, v_{ij}) \\
&\times f_v(v_{ij} \mid r_i = 1, \bar{\boldsymbol{v}}_{ij}, \mathbf{x}_i)
\end{aligned} \tag{S-3}
$$

where from (1) it follows that $\Pr(y^*_{ij} = 1 \mid g_i = 1, t_i, v_{ij}) = 1$ and $\Pr(y^*_{ij} = 1 \mid g_i = 0, t_i, v_{ij}) = \mathbb{1}_{\{v_{ij} \geq t_i\}}$, and from (3) we have

$$
\Pr(y_{ij} = y \mid v_{ij}, y^*_{ij}, \kappa) = \big(\kappa y^*_{ij} \mathbb{1}_{\{v_{ij} < \infty\}}\big)^y \big(1 - \kappa y^*_{ij} \mathbb{1}_{\{v_{ij} < \infty\}}\big)^{1-y}, \quad y \in \{0, 1\}. \tag{S-4}
$$

Since the last term in (S-3) does not depend on $(\boldsymbol{\beta}, \sigma, \boldsymbol{\theta}, \kappa)$, t_i or g_i it can be ignored in the likelihood. The remaining term is the likelihood of the observed test outcomes and will be denoted

$$
\Pr(\mathbf{y}_i \mid g_i, r_i = 1, t_i, \mathbf{v}_i, \kappa) = \prod_{j=1}^{c_i} \sum_{m=0}^{1} \Pr(y_{ij} \mid v_{ij}, y^*_{ij} = m, \kappa) \Pr(y^*_{ij} = m \mid g_i, t_i, v_{ij}), \tag{S-5}
$$

so that (S-2) simplifies as follows

$$
\begin{aligned}
f_{r,\mathbf{v},\mathbf{y}_{\text{com}}}(r_i = 1, \mathbf{v}_i, \mathbf{y}_i \mid \mathbf{x}_i, \boldsymbol{\beta}, \sigma, \boldsymbol{\theta}, \kappa) &\propto (1 - \Phi(\mu_i)) \int_0^\infty f_t(t_i \mid \mathbf{x}_i, \boldsymbol{\beta}, \sigma) \Pr(\mathbf{y}_i \mid g_i = 0, r_i = 1, t_i, \mathbf{v}_i, \kappa) dt_i \\
&+ \Phi(\mu_i) \int_0^\infty f_t(t_i \mid \mathbf{x}_i, \boldsymbol{\beta}, \sigma) \Pr(\mathbf{y}_i \mid g_i = 1, r_i = 1, t_i, \mathbf{v}_i, \kappa) dt_i.
\end{aligned} \tag{S-6}
$$

We derive $\Pr(\mathbf{y}_i \mid g_i, r_i = 1, t_i, \mathbf{v}_i, \kappa)$ below for prevalent and non-prevalent conditioning.

A.1.3 Special case: Likelihood contribution from positive baseline tests

A special case now arises when $r_i = 1$ and $y_{i1} = 1$ (positive baseline test). This implies that, in (S-5), $\Pr(y_{i1} = 1 \mid v_{i1}, y^*_{i1} = 0, \kappa) = 0$ where $y^*_{i1} = 0$ with probability one in case of non-prevalence ($g_i = 0$). Furthermore, $y^*_{i1} = 1$ with probability one in case of prevalence ($g_i = 1$), with $\Pr(y_{i1} = 1 \mid v_{i1}, y^*_{i1} = 1, \kappa) = \kappa$. Hence,

$$\Pr(y_{i1}=1 \mid g_i=1, r_i=1, t_i, v_{i1}, \kappa) = \kappa \tag{S-7}$$

and

$$\Pr(y_{i1}=1 \mid g_i=0, r_i=1, t_i, v_{i1}, \kappa) = 0 \tag{S-8}$$

Therefore, if $y_{i1}=1$, (S-6) simplifies as

$$\begin{aligned} f_{r,\mathbf{v},\mathbf{y}_{\text{com}}}(r_i=1, \mathbf{v}_i=(v_{i1}), \mathbf{y}_i=(1) \mid \mathbf{x}_i, \boldsymbol{\beta}, \sigma, \boldsymbol{\theta}, \kappa) \propto (1-\Phi(\mu_i)) &\int_0^\infty f_t(t_i \mid \mathbf{x}_i, \boldsymbol{\beta}, \sigma) \times 0 \; dt_i \\ &+ \Phi(\mu_i) \int_0^\infty f_t(t_i \mid \mathbf{x}_i, \boldsymbol{\beta}, \sigma) \times \kappa \; dt_i \\ &= \Phi(\mu_i)\; \kappa. \end{aligned} \tag{S-9}$$

This effectively collapses the mixture structure of the likelihood over latent g_i: the non-prevalent component has zero likelihood, so $g_i = 1$ is known whenever the baseline test is positive (assumption of perfect test specificity), while the baseline test still contributes information through the factor κ.

A.1.4 Likelihood contribution of the test outcomes when the baseline test is missing ($r_i = 0$)

Without a baseline test ($r_i = 0$), we have that y_{i1} in $\mathbf{y}_{i,\text{com}} = (y_{i1}, \mathbf{y}_i)$ is missing and integrated out of the likelihood. From (S-1)–(S-2)we have

$$\begin{aligned} &\sum_{k=0}^{1} f_{r,\mathbf{v},\mathbf{y}_{\text{com}}}(r_i=0, \mathbf{v}_i, (y_{i1}=k, \mathbf{y}_i) \mid \mathbf{x}_i, \boldsymbol{\beta}, \sigma, \boldsymbol{\theta}, \kappa) \\ &\propto \sum_{l=0}^{1} \Phi(\mu_i)^l (1-\Phi(\mu_i))^{(1-l)} \int_0^\infty f_t(t_i \mid \mathbf{x}_i, \boldsymbol{\beta}, \sigma) \sum_{k=0}^{1} f_{\mathbf{v},\mathbf{y}_{\text{com}}}(\mathbf{v}_i, (y_{i1}=k, \mathbf{y}_i) \mid g_i=l, r_i=0, t_i, \mathbf{x}_i, \kappa) dt_i \end{aligned} \tag{S-10}$$

where

$$\begin{aligned} \sum_{k=0}^{1} f_{\mathbf{v},\mathbf{y}_{\text{com}}}(\mathbf{v}_i, (y_{i1}=k, \mathbf{y}_i) \mid g_i=l, r_i=0, t_i, \mathbf{x}_i, \kappa) &= \left[\sum_{k=0}^{1} f_{v,y}(v_{i1}, y_{i1}=k \mid g_i=l, r_i=0, t_i, \mathbf{x}_i, \kappa) \right] \\ &\times \prod_{j=2}^{c_i} f_{v,y}(v_{ij}, y_{ij} \mid g_i=l, r_i=0, t_i, \bar{\boldsymbol{v}}_{ij}, \mathbf{x}_i, \kappa) \\ &= f_v(v_{i1} \mid r_i=0, \mathbf{x}_i) \\ &\times \prod_{j=2}^{c_i} f_{v,y}(v_{ij}, y_{ij} \mid g_i=l, r_i=0, t_i, \bar{\boldsymbol{v}}_{ij}, \mathbf{x}_i, \kappa) \\ &\propto \prod_{j=2}^{c_i} f_{v,y}(v_{ij}, y_{ij} \mid g_i=l, r_i=0, t_i, \bar{\boldsymbol{v}}_{ij}, \mathbf{x}_i, \kappa), \end{aligned} \tag{S-11}$$

where the first equality and the proportionality hold because

$$\sum_{k=0}^{1} f_{v,y}(v_{i1}, y_{i1}=k \mid g_i, r_i=0, t_i, \mathbf{x}_i, \kappa) = \sum_{k=0}^{1}\sum_{m=0}^{1} \Pr(y_{i1}=k \mid v_{i1}, y^*_{i1}=m, \kappa) \times \Pr(y^*_{i1}=m \mid g_i, t_i, v_{i1}) f_v(v_{i1} \mid r_i=0, \mathbf{x}_i) = f_v(v_{i1} \mid r_i=0, \mathbf{x}_i) \propto 1. \tag{S-12}$$

Therefore, using the same results as in (S-3) and (S-5) we have that regardless of prevalence status the likelihood of the observed test outcomes (i.e. all test outcomes except the baseline test) is

$$\Pr(\mathbf{y}_i \mid g_i, r_i=0, t_i, \mathbf{v}_i, \kappa) = \prod_{j=2}^{c_i}\sum_{m=0}^{1} \Pr(y_{ij} \mid v_{ij}, y^*_{ij}=m, \kappa)\Pr(y^*_{ij}=m \mid g_i, t_i, v_{ij}). \tag{S-13}$$

Hence, when $r_i = 0$, the observed test outcomes correspond to occasions indexed $j = 2, \ldots, c_i$, and the likelihood factorizes as a product over these observed occasions. Result (S-13) is equivalent to result (S-5) except that the factorization starts at the first moment after baseline ($j = 2$) instead of at baseline which "ignores" baseline outcomes in individuals for whom the outcome is missing (Little and Rubin, 2002).

As a result we have that (S-2) simplifies as follows

$$f_{r,\mathbf{v},\mathbf{y}_{\text{com}}}(r_i=0, \mathbf{v}_i, \mathbf{y}_i \mid \mathbf{x}_i, \boldsymbol{\beta}, \sigma, \boldsymbol{\theta}, \kappa) \propto (1-\Phi(\mu_i))\int_0^\infty f_t(t_i \mid \mathbf{x}_i, \boldsymbol{\beta}, \sigma)\Pr(\mathbf{y}_i \mid g_i=0, r_i=0, t_i, \mathbf{v}_i, \kappa)dt_i + \Phi(\mu_i)\int_0^\infty f_t(t_i \mid \mathbf{x}_i, \boldsymbol{\beta}, \sigma)\Pr(\mathbf{y}_i \mid g_i=1, r_i=0, t_i, \mathbf{v}_i, \kappa)dt_i. \tag{S-14}$$

We derive $\Pr(\mathbf{y}_i \mid g_i, r_i=0, t_i, \mathbf{v}_i, \kappa)$ below for prevalent and non-prevalent conditioning.

A.1.5 Likelihood contribution of the test outcomes from non-prevalent individuals

We now derive $\Pr(\mathbf{y}_i \mid g_i=0, r_i=1, t_i, \mathbf{v}_i, \kappa)$ and $\Pr(\mathbf{y}_i \mid g_i=0, r_i=0, t_i, \mathbf{v}_i, \kappa)$ in (S-6) and (S-14) by plugging probability (S-4) together with $\Pr(y^*_{ij}=1 \mid g_i=0, t_i, v_{ij}) = \mathbb{1}_{\{v_{ij}\geq t_i\}}$ into (S-5) and (S-13).

We first note that with $g_i = 0$ (non-prevalence) all baseline tests that are done ($r_i = 1$) are necessarily negative (no false positives). Formally, $\Pr(y_{i1}=0 \mid v_{i1}=0, y^*_{i1}=0, \kappa) = 1$ with $\Pr(y^*_{i1}=0 \mid g_i=0, t_i, v_{i1}=0) = 1$. Therefore:

$$\begin{aligned}\Pr(\mathbf{y}_i \mid g_i=0, r_i=1, t_i, \mathbf{v}_i, \kappa) &= \Pr(\mathbf{y}_i \mid g_i=0, r_i=0, t_i, \mathbf{v}_i, \kappa)\\ &= \prod_{j=2}^{c_i}\left[\mathbb{1}_{\{v_{ij}\geq t_i\}}(1-\kappa)^{(1-y_{ij})\mathbb{1}_{\{v_{ij}<\infty\}}}\kappa^{y_{ij}} + \mathbb{1}_{\{v_{ij}<t_i\}}(1-y_{ij})\right]\\ &= \left[\prod_{j:v_{ij}\geq t_i}(1-\kappa)^{(1-y_{ij})\mathbb{1}_{\{v_{ij}<\infty\}}}\kappa^{y_{ij}}\right]\times\left[\prod_{j:v_{ij}<t_i}(1-y_{ij})\right]\\ &= \begin{cases}\prod_{j:v_{ij}\geq t_i}(1-\kappa)^{(1-y_{ij})\mathbb{1}_{\{v_{ij}<\infty\}}}\kappa^{y_{ij}} & \text{if } v_{ic_i}\geq t_i\\ 0 & \text{if } v_{ic_i}<t_i.\end{cases}\end{aligned} \tag{S-15}$$

The restriction $\Pr(\mathbf{y}_i \mid g_i = 0, t_i, \mathbf{v}_i, \kappa) = 0$ if $v_{ic_i} < t_i$ emerges, because

$$\prod_{j:v_{ij}<t_i} (1 - y_{ij}) = (1 - 0) \times (1 - 0) \times \cdots \times (1 - y_{ic_i}) = 0 \quad \text{if } v_{ic_i} < t_i,$$

since whenever $v_{ic_i} < t_i$ we have that $y_{ic_i} = 1$ (note that $v_{ic_i} < t_i$ cannot occur in case of right censoring because then $v_{ic_i} = \infty$). In other words, the situation that $v_{ic_i} < t_i$ while a test was positive (event) has zero likelihood and hence cannot occur in the data (no false positive tests allowed). Technically, this restriction is relevant, because (S-2) integrates t_i in $\Pr(\mathbf{y}_i \mid g_i = 0, t_i, \mathbf{v}_i, \kappa)$ unrestricted over its full support $(0, \infty)$.

For $v_{ic_i} \geq t_i$, we have

$$\begin{aligned}
\Pr(\mathbf{y}_i \mid g_i = 0, t_i, \mathbf{v}_i, \kappa) &= (1-\kappa)^{\sum_{j:v_{ij}\geq t_i}(1-y_{ij})\mathbb{1}_{\{v_{ij}<\infty\}}} \kappa^{\sum_{j:v_{ij}\geq t_i} y_{ij}} \\
&= (1-\kappa)^{[\sum_{j:v_{ij}\geq t_i} 1]-1} \kappa^{\sum_{j:v_{ij}\geq t_i} y_{ij}} \\
&= (1-\kappa)^{\sum_{j=1}^{c_i}[\mathbb{1}_{\{v_{ij}\geq t_i\}}]-1} \kappa^{y_{ic_i}} \\
&= (1-\kappa)^{m_i} \kappa^{y_{ic_i}}, \qquad \text{(S-16)}
\end{aligned}$$

with $m_i = \sum_{j=1}^{c_i}[\mathbb{1}_{\{v_{ij}\geq t_i\}}] - 1 = \sum_{j=1}^{c_i-1} y_{ij}^*$ the number of falsely negative screening tests until v_{ic_i}. For the second equation, we have used from (S-4) the fact that $y_{ij} = 0$ whenever $y_{ij}^* = 0$ (i.e., $v_{ij} < t_i$). For the third equation, we have used the fact that $y_{ij} = 0$ for all j in $\mathbf{y}_i$ except for the last element y_{ic_i}. If $y_{ic_i} = 1$ (event at $v_{ic_i} < \infty$), $1 - y_{ic_i} = 0$. If $y_{ic_i} = 0$ we have right censoring with $v_{ic_i} = \infty$, so that $(1 - y_{ic_i})\mathbb{1}_{\{v_{ic_i}<\infty\}} = 0$. Hence

$$\Pr(\mathbf{y}_i \mid g_i = 0, t_i, \mathbf{v}_i, \kappa) = \begin{cases} (1-\kappa)^{m_i}\kappa^{y_{ic_i}} & \text{if } v_{ic_i} \geq t_i \\ 0 & \text{if } v_{ic_i} < t_i. \end{cases} \qquad \text{(S-17)}$$

A useful result is now that (S-17) can be written as a sum

$$\Pr(\mathbf{y}_i \mid g_i = 0, t_i, \mathbf{v}_i, \kappa) = \kappa^{y_{ic_i}} \sum_{j=1}^{c_i-1} (1-\kappa)^{(c_i-j-1)} \mathbb{1}_{\{v_{ij}<t_i\leq v_{ij+1}\}}, \qquad \text{(S-18)}$$

where we emphasize again that we consider either individuals without baseline test ($r_i = 0$) or negative baseline test $y_{i1} = 0$, so that $c_i > 1$. For the special case when $y_{i1} = 1$, see (S-9).

After plugging back into (S-6) we obtain closed form integrals over t_i

$$\begin{aligned}
&\int_0^\infty f_t(t_i \mid \mathbf{x}_i, \boldsymbol{\beta}, \sigma) \Pr(\mathbf{y}_i \mid g_i = 0, r_i = 1, t_i, \mathbf{v}_i, \kappa) dt_i \\
&= \int_0^\infty f_t(t_i \mid \mathbf{x}_i, \boldsymbol{\beta}, \sigma) \kappa^{y_{ic_i}} \sum_{j=1}^{c_i-1} (1-\kappa)^{(c_i-j-1)} \mathbb{1}_{\{v_{ij}<t_i\leq v_{ij+1}\}} dt_i \\
&= \kappa^{y_{ic_i}} \sum_{j=1}^{c_i-1} (1-\kappa)^{(c_i-j-1)} \big[F_t(v_{ij+1} \mid \mathbf{x}_i, \boldsymbol{\beta}, \sigma) - F_t(v_{ij} \mid \mathbf{x}_i, \boldsymbol{\beta}, \sigma)\big]. \qquad \text{(S-19)}
\end{aligned}$$

The same result follows for $r_i = 0$ in (S-14).

A.1.6 Likelihood contribution of the test outcomes from prevalent individuals

We now derive $\Pr(\mathbf{y}_i \mid g_i = 1, r_i = 1, t_i, \mathbf{v}_i, \kappa)$ and $\Pr(\mathbf{y}_i \mid g_i = 1, r_i = 0, t_i, \mathbf{v}_i, \kappa)$ in (S-6) and (S-14) for prevalent individuals by plugging in probability (S-4) together with $\Pr(y_{ij}^* = 1 \mid g_i =$

$1, t_i, v_{ij}) = 1$ in (S-5) and (S-13).

In case of prevalence ($g_i = 1$) and baseline tests ($r_i = 1$) we have

$$\Pr(\mathbf{y}_i \mid g_i = 1, r_i = 1, t_i, \mathbf{v}_i, \kappa) = \prod_{j=1}^{c_i} \kappa^{y_{ij}} (1-\kappa)^{(1-y_{ij})\mathbb{1}_{\{v_{ij}<\infty\}}} = \kappa^{y_{ic_i}} (1-\kappa)^{(c_i-1)}. \tag{S-20}$$

In case of prevalence ($g_i = 1$) and no baseline tests ($r_i = 0$), we have

$$\Pr(\mathbf{y}_i \mid g_i = 1, r_i = 0, t_i, \mathbf{v}_i, \kappa) = \prod_{j=2}^{c_i} \kappa^{y_{ij}} (1-\kappa)^{(1-y_{ij})\mathbb{1}_{\{v_{ij}<\infty\}}} = \kappa^{y_{ic_i}} (1-\kappa)^{(c_i-2)}. \tag{S-21}$$

Combining these results we obtain the general result

$$\Pr(\mathbf{y}_i \mid g_i = 1, r_i, t_i, \mathbf{v}_i, \kappa) = \kappa^{y_{ic_i}} (1-\kappa)^{(c_i+r_i-2)}. \tag{S-22}$$

After plugging back into (S-6) and (S-14), we may integrate t_i out, obtaining

$$\begin{aligned} &\int_0^\infty f_t(t_i \mid \mathbf{x}_i, \boldsymbol{\beta}, \sigma) \Pr(\mathbf{y}_i \mid g_i = 1, r_i, t_i, \mathbf{v}_i, \kappa) dt_i \\ &= \kappa^{y_{ic_i}} (1-\kappa)^{(c_i+r_i-2)}, \end{aligned} \tag{S-23}$$

where we give a general expression for both $r_i = 1$ and $r_i = 0$.

Note that in the special case of a positive baseline test (i.e. $r_i = 1$, $c_i = 1$, $y_{i1} = 1$ so $g_i = 1$), equation (S-22) reduces to $\Pr(\mathbf{y}_i \mid g_i = 1, r_i = 1, t_i, \mathbf{v}_i, \kappa) = \kappa$, which is consistent with result (S-9). In this situation the non-prevalent component of (S-6) has zero likelihood, so the mixture over g_i collapses.

A.1.7 Full expression for the observed-data likelihood

We are now ready to write down a complete expression for the observed-data likelihood (S-1)–(S-2). Substituting (S-6) and (S-14) into (S-2) and factoring in result (S-9), we obtain

$$\begin{aligned}\mathcal{L}(\boldsymbol{\beta}, \sigma, \boldsymbol{\theta}, \kappa \mid \boldsymbol{\mathcal{D}}) \propto \prod_{i: r_i=1, y_{i1}=0} & \Big[(1-\Phi(\mu_i)) \int_0^\infty f_t(t_i \mid \mathbf{x}_i, \boldsymbol{\beta}, \sigma) \Pr(\mathbf{y}_i \mid g_i = 0, r_i = 1, t_i, \mathbf{v}_i, \kappa) dt_i \\ & + \Phi(\mu_i) \int_0^\infty f_t(t_i \mid \mathbf{x}_i, \boldsymbol{\beta}, \sigma) \Pr(\mathbf{y}_i \mid g_i = 1, r_i = 1, t_i, \mathbf{v}_i, \kappa) dt_i\Big] \\ & \times \prod_{i: r_i=0} \Big[(1-\Phi(\mu_i)) \int_0^\infty f_t(t_i \mid \mathbf{x}_i, \boldsymbol{\beta}, \sigma) \Pr(\mathbf{y}_i \mid g_i = 0, r_i = 0, t_i, \mathbf{v}_i, \kappa) dt_i \\ & + \Phi(\mu_i) \int_0^\infty f_t(t_i \mid \mathbf{x}_i, \boldsymbol{\beta}, \sigma) \Pr(\mathbf{y}_i \mid g_i = 1, r_i = 0, t_i, \mathbf{v}_i, \kappa) dt_i\Big] \\ & \times \prod_{i: r_i=1, y_{i1}=1} \Phi(\mu_i)\ \kappa \end{aligned} \tag{S-24}$$

Now substituting results (S-19) and (S-23) we obtain that $\mathcal{L}(\boldsymbol{\beta}, \sigma, \boldsymbol{\theta}, \kappa \mid \boldsymbol{\mathcal{D}})$ is proportional to

$$\begin{aligned}\prod_{i: r_i=1, y_{i1}=0} & \Big[(1-\Phi(\mu_i))\kappa^{y_{ic_i}} \sum_{j=1}^{c_i-1} (1-\kappa)^{(c_i-j-1)} \big[F_t(v_{ij+1} \mid \mathbf{x}_i, \boldsymbol{\beta}, \sigma) - F_t(v_{ij} \mid \mathbf{x}_i, \boldsymbol{\beta}, \sigma)\big] \\ & + \Phi(\mu_i)\kappa^{y_{ic_i}}(1-\kappa)^{(c_i-1)}\Big] \\ & \times \prod_{i: r_i=0} \Big[(1-\Phi(\mu_i))\kappa^{y_{ic_i}} \sum_{j=1}^{c_i-1} (1-\kappa)^{(c_i-j-1)} \big[F_t(v_{ij+1} \mid \mathbf{x}_i, \boldsymbol{\beta}, \sigma) - F_t(v_{ij} \mid \mathbf{x}_i, \boldsymbol{\beta}, \sigma)\big] \\ & + \Phi(\mu_i)\kappa^{y_{ic_i}}(1-\kappa)^{(c_i-2)}\Big] \\ & \times \prod_{i: r_i=1, y_{i1}=1} \Phi(\mu_i)\ \kappa \end{aligned} \tag{S-25}$$

which is more compactly written as

$$\begin{aligned}\prod_{i \in \mathcal{I}_0} & \Big[(1-\Phi(\mu_i))\kappa^{y_{ic_i}} \sum_{j=1}^{c_i-1} (1-\kappa)^{(c_i-j-1)} \big[F_t(v_{ij+1} \mid \mathbf{x}_i, \boldsymbol{\beta}, \sigma) - F_t(v_{ij} \mid \mathbf{x}_i, \boldsymbol{\beta}, \sigma)\big] \\ & + \Phi(\mu_i)\kappa^{y_{ic_i}}(1-\kappa)^{(c_i+r_i-2)}\Big] \times \prod_{i \in \mathcal{I}_1} \Phi(\mu_i)\ \kappa. \end{aligned} \tag{S-26}$$

where $\mathcal{I}_0$ denotes the set of all individuals i with a negative baseline test ($r_i = 1$ and $y_{i1} = 0$) or a missing baseline test ($r_i = 0$) and $\mathcal{I}_1$ denotes the set of all individuals i with a positive baseline test ($r_i = 1$ and $y_{i1} = 1$).

A.2 Gibbs sampler

A.2.1 Notation

To derive the full conditional distributions used in the Gibbs sampler, we will apply a full factorization of joint density of the random variables and the data. In the factorization, the joint likelihood of the test outcomes and screening times (S-2) will appear, but depending on whether a baseline test outcome is observed, the likelihood is either integrated over missing outcomes or not integrated (i.e., all outcomes observed); see Supplemental Material Section A.1. To facilitate the subsequent derivations, we introduce a unifying notation for the joint likelihood of screening times and the observed test outcomes as follows:

$$\tilde{f}_{\mathbf{v},\mathbf{y}}(\mathbf{v}_i, \mathbf{y}_i \mid g_i, r_i, t_i, \mathbf{x}_i, \kappa) = \begin{cases} f_{\mathbf{v},\mathbf{y}_{\text{com}}}(\mathbf{v}_i, \mathbf{y}_i \mid g_i, r_i = 1, t_i, \mathbf{x}_i, \kappa), & \text{if } r_i = 1 \\ \sum_{k=0}^{1} f_{\mathbf{v},\mathbf{y}_{\text{com}}}(\mathbf{v}_i, (y_{i1}, \mathbf{y}_i) \mid g_i, r_i = 0, t_i, \mathbf{x}_i, \kappa), & \text{if } r_i = 0 \end{cases} \tag{S-27}$$

The two likelihoods for $r_i = 1$ and $r_i = 0$ are derived in equation (S-3) and (S-13), respectively. The special case of a positive baseline test is given in (S-9). Specifically,

$$\tilde{f}_{\mathbf{v},\mathbf{y}}(\mathbf{v}_i, \mathbf{y}_i \mid g_i, r_i, t_i, \mathbf{x}_i, \kappa) \propto \begin{cases} \Pr(\mathbf{y}_i \mid g_i, r_i = 1, t_i, \mathbf{v}_i, \kappa), & \text{if } r_i = 1 \\ \Pr(\mathbf{y}_i \mid g_i, r_i = 0, t_i, \mathbf{v}_i, \kappa), & \text{if } r_i = 0 \end{cases} \tag{S-28}$$

where $\Pr(\mathbf{y}_i \mid g_i = 0, r_i = 1, t_i, \mathbf{v}_i, \kappa) = \Pr(\mathbf{y}_i \mid g_i = 0, r_i = 0, t_i, \mathbf{v}_i, \kappa)$ is given in equation (S-17) with its rewritten sum representation in (S-18) and $\Pr(\mathbf{y}_i \mid g_i = 1, r_i, t_i, \mathbf{v}_i, \kappa)$ is given in (S-22).

A.2.2 Full conditional distribution of t_i

We derive the full conditional distribution of t_i. We first consider the non-prevalent case ($g_i = 0$), i.e.

$$
\begin{aligned}
f_t(t_i \mid \mathcal{D}_i, g_i = 0, \boldsymbol{\beta}, \sigma, \boldsymbol{\theta}, \kappa) &\propto f_{g,r,t,\mathbf{v},\mathbf{y}}(g_i = 0, r_i, t_i, \mathbf{v}_i, \mathbf{y}_i \mid \mathbf{x}_i, \boldsymbol{\beta}, \sigma, \boldsymbol{\theta}, \kappa) \\
&= f_g(g_i = 0 \mid \mathbf{x}_i, \boldsymbol{\theta}) f_r(r_i \mid \mathbf{x}_i) f_t(t_i \mid \mathbf{x}_i, \boldsymbol{\beta}, \sigma) \tilde{f}_{\mathbf{v},\mathbf{y}}(\mathbf{v}_i, \mathbf{y}_i \mid g_i = 0, r_i, t_i, \mathbf{x}_i, \kappa) \\
&\propto \ f_t(t_i \mid \mathbf{x}_i, \boldsymbol{\beta}, \sigma) \Pr(\mathbf{y}_i \mid g_i = 0, r_i, t_i, \mathbf{v}_i, \kappa) \\
&= \sum_{j=1}^{c_i - 1} \ f_t(t_i \mid \mathbf{x}_i, \boldsymbol{\beta}, \sigma) \ \left(\kappa^{y_{ic_i}} (1-\kappa)^{(c_i - j - 1)} \mathbb{1}_{\{v_{ij} < t_i \leq v_{ij+1}\}}\right) \\
&= \sum_{j=1}^{c_i - 1} f_t(t_i \mid v_{ij} < t_i \leq v_{ij+1}, \mathbf{x}_i, \boldsymbol{\beta}, \sigma) \\
&\times \left[F_t(v_{ij+1} \mid \mathbf{x}_i, \boldsymbol{\beta}, \sigma) - F_t(v_{ij} \mid \mathbf{x}_i, \boldsymbol{\beta}, \sigma)\right] \ \left(\kappa^{y_{ic_i}} (1-\kappa)^{(c_i - j - 1)}\right) \\
&= \sum_{j=1}^{c_i - 1} \tilde{\omega}_{ij} f_t(t_i \mid v_{ij} < t_i \leq v_{ij+1}, \mathbf{x}_i, \boldsymbol{\beta}, \sigma),
\end{aligned} \tag{S-29}
$$

where

$$
\tilde{\omega}_{ij} = \kappa^{y_{ic_i}} (1-\kappa)^{(c_i - j - 1)} \big[F_t(v_{ij+1} \mid \mathbf{x}_i, \boldsymbol{\beta}, \sigma) - F_t(v_{ij} \mid \mathbf{x}_i, \boldsymbol{\beta}, \sigma)\big]. \tag{S-30}
$$

The second equation follows from the factorization into the joint data generating densities through the hierarchical model described in Section 3.2 and the DAG (Figure 1) together with notation (S-27). The third equation (proportionality) follows due to independence of various terms from t_i and after applying the result (S-28). The fourth equation substitutes result (S-18), the sum representation of the likelihood of the test outcomes under $g_i = 0$. The fifth equation uses the fact that $\mathbb{1}_{\{v_{ij} < t_i \leq v_{ij+1}\}} f_t(t_i \mid \mathbf{x}_i, \boldsymbol{\beta}, \sigma)$ is an unnormalized truncated distribution. The last two factors in the fifth equation can then be recognized as the weights $\tilde{\omega}_{ij}$ of an unnormalized mixture of truncated distributions of t_i; see the sixth equation. After normalizing the weights through

$$
\omega_{ij} = \frac{\tilde{\omega}_{ij}}{\sum_{l=1}^{c_i - 1} \tilde{\omega}_{il}} \tag{S-31}
$$

the result in (15) is obtained, i.e.

$$
f_t(t_i \mid \mathcal{D}_i, g_i = 0, \boldsymbol{\beta}, \sigma, \kappa) = \sum_{j=1}^{c_i - 1} \omega_{ij} f_t(t_i \mid v_{ij} < t_i \leq v_{ij+1}, \mathbf{x}_i, \boldsymbol{\beta}, \sigma) \tag{S-32}
$$

Finally, in the prevalent case, when $g_i = 1$, we have after substituting (S-22)

$$
f_t(t_i \mid \mathcal{D}_i, g_i = 1, \boldsymbol{\beta}, \sigma, \boldsymbol{\theta}, \kappa) \propto f_t(t_i \mid \mathbf{x}_i, \boldsymbol{\beta}, \sigma)\left(\kappa^{y_{ic_i}} (1-\kappa)^{(c_i + r_i - 2)}\right) \propto f_t(t_i \mid \mathbf{x}_i, \boldsymbol{\beta}, \sigma), \tag{S-33}
$$

irrespective of $r_i = 0$ or $r_i = 1$. Hence, t_i is updated uninformatively when $g_i = 1$.

A.2.3 Full conditional distribution and collapsed distribution of g_i

We begin by noting again, as in Section 3.1, that $g_i = 1$ is known if $r_i = 1$ and $y_{i1} = 1$ (positive baseline test). In all other cases g_i is missing (latent) and augmented as part of the Gibbs sampler. We now derive the full conditional distribution of (missing) g_i. We have

$$
\begin{aligned}
f_g(g_i \mid \mathcal{D}_i, t_i, \boldsymbol{\beta}, \sigma, \boldsymbol{\theta}, \kappa) &\propto f_{g,r,t,\mathbf{v},\mathbf{y}}(g_i, r_i, t_i, \mathbf{v}_i, \mathbf{y}_i \mid \mathbf{x}_i, \boldsymbol{\beta}, \sigma, \boldsymbol{\theta}, \kappa) \\
&= f_g(g_i \mid \mathbf{x}_i, \boldsymbol{\theta}) f_r(r_i \mid \mathbf{x}_i) f_t(t_i \mid \mathbf{x}_i, \boldsymbol{\beta}, \sigma) \tilde{f}_{\mathbf{v},\mathbf{y}}(\mathbf{v}_i, \mathbf{y}_i \mid g_i, r_i, t_i, \mathbf{x}_i, \kappa) \\
&\propto f_g(g_i \mid \mathbf{x}_i, \boldsymbol{\theta}) \Pr(\mathbf{y}_i \mid g_i, r_i, t_i, \mathbf{v}_i, \kappa) \\
&= \begin{cases} 0 & \text{if } g_i = 0 \text{ and } t_i > v_{ic_i} \\ \big(1 - \Phi(\mu_i)\big) \kappa^{y_{ic_i}} (1-\kappa)^{m_i} & \text{if } g_i = 0 \text{ and } t_i \le v_{ic_i} \\ \Phi(\mu_i) \kappa^{y_{ic_i}} (1-\kappa)^{(c_i + r_i - 2)} & \text{if } g_i = 1. \end{cases}
\end{aligned} \tag{S-34}
$$

The second equation follows from the factorization into the joint data generating densities through the hierarchical model described in Section 3.2 and the DAG (Figure 1) together with notation (S-27). The third equation (proportionality) follows due to independence of various terms from g_i and after applying the result (S-27). The fourth equation substitutes results (S-17) and (S-22). Hence, after normalizing,

$$
f_g(g_i = 1 \mid \mathcal{D}_i, t_i, \boldsymbol{\theta}, \kappa) = \begin{cases} \dfrac{\Phi(\mu_i)\kappa^{y_{ic_i}}(1-\kappa)^{(c_i+r_i-2)}}{\Phi(\mu_i)\kappa^{y_{ic_i}}(1-\kappa)^{(c_i+r_i-2)} + \big(1-\Phi(\mu_i)\big)\kappa^{y_{ic_i}}(1-\kappa)^{m_i}} & \text{if } t_i \le v_{ic_i} \\ 1 & \text{if } t_i > v_{ic_i}. \end{cases} \tag{S-35}
$$

As can be seen from (S-35), g_i is updated to one with probability one (deterministic update), except when $t_i \le v_{ic_i}$ (stochastic update). The determinacy can cause inefficiencies in the Gibbs sampler due to the following feedback. Suppose $g_i^{(k)} = 1$ at any point in the Gibbs sampler, then t_i is updated uninformatively in $(0, \infty)$ so that $t_i^{(k+1)} > v_{ic_i}$ has positive probability. In that case, $g_i^{(k+1)} = 1$ with probability one in the next step. This dependency continues unless in a future draw $t_i^{(k')} \le v_{ic_i}$, in which case $g_i^{(k')} = 0$ has positive probability again.

To avoid this problem, we integrate additionally over the latent times t_i and obtain

$$
\begin{aligned}
f_g(g_i \mid \mathcal{D}_i, \boldsymbol{\beta}, \sigma, \boldsymbol{\theta}, \kappa) &\propto \int_0^\infty f_{g,r,t,\mathbf{v},\mathbf{y}}(g_i, r_i, t_i, \mathbf{v}_i, \mathbf{y}_i \mid \mathbf{x}_i, \boldsymbol{\beta}, \sigma, \boldsymbol{\theta}, \kappa) dt_i \\
&= \int_0^\infty f_g(g_i \mid \mathbf{x}_i, \boldsymbol{\theta}) f_r(r_i \mid \mathbf{x}_i) f_t(t_i \mid \mathbf{x}_i, \boldsymbol{\beta}, \sigma) \tilde{f}_{\mathbf{v},\mathbf{y}}(\mathbf{v}_i, \mathbf{y}_i \mid g_i, r_i, t_i, \mathbf{x}_i, \kappa) dt_i \\
&\propto \int_0^\infty f_g(g_i \mid \mathbf{x}_i, \boldsymbol{\theta}) f_t(t_i \mid \mathbf{x}_i, \boldsymbol{\beta}, \sigma) \Pr(\mathbf{y}_i \mid g_i, r_i, t_i, \mathbf{v}_i, \kappa) dt_i
\end{aligned} \tag{S-36}
$$

Hence, $f_g(g_i = 0 \mid \mathcal{D}_i, \boldsymbol{\beta}, \sigma, \boldsymbol{\theta}, \kappa)$ is proportional to

$$
\begin{aligned}
&\big(1 - \Phi(\mu_i)\big) \int_0^\infty \sum_{j=1}^{c_i - 1} \kappa^{y_{ic_i}} (1-\kappa)^{(c_i - j - 1)} \mathbb{1}_{\{v_{ij} < t_i \le v_{ij+1}\}} f_t(t_i \mid \mathbf{x}_i, \boldsymbol{\beta}, \sigma) dt_i \\
&= \big(1 - \Phi(\mu_i)\big) \sum_{j=1}^{c_i - 1} \kappa^{y_{ic_i}} (1-\kappa)^{(c_i - j - 1)} \big[F_t(v_{ij+1} \mid \mathbf{x}_i, \boldsymbol{\beta}, \sigma) - F_t(v_{ij} \mid \mathbf{x}_i, \boldsymbol{\beta}, \sigma)\big] \\
&= \big(1 - \Phi(\mu_i)\big) \sum_{j=1}^{c_i - 1} \tilde{\omega}_{ij}
\end{aligned} \tag{S-37}
$$

where in the first equation (proportionality) we used result (S-18) and in the last equation definition (S-30). Furthermore, $f_g(g_i = 1 \mid \mathcal{D}_i, \boldsymbol{\beta}, \sigma, \boldsymbol{\theta}, \kappa)$ is proportional to

$$\Phi(\mu_i) \int_0^{\infty} \kappa^{y_{ic_i}} (1-\kappa)^{(c_i+r_i-2)} f_t(t_i \mid \mathbf{x}_i, \boldsymbol{\beta}, \sigma) dt_i = \Phi(\mu_i) \kappa^{y_{ic_i}} (1-\kappa)^{(c_i+r_i-2)} \tag{S-38}$$

so that after normalization

$$f_g(g_i = 1 \mid \mathcal{D}_i, \boldsymbol{\beta}, \sigma, \boldsymbol{\theta}, \kappa) = \frac{\Phi(\mu_i) \kappa^{y_{ic_i}} (1-\kappa)^{(c_i+r_i-2)}}{\Phi(\mu_i) \kappa^{y_{ic_i}} (1-\kappa)^{(c_i+r_i-2)} + (1-\Phi(\mu_i)) \sum_{l=1}^{c_i-1} \tilde{\omega}_{il}}. \tag{S-39}$$

A.2.4 Updating the parameters of t_i

We begin by noticing that the full conditional distribution of $(\boldsymbol{\beta}, \sigma)$ is proportional to a distribution of $(\boldsymbol{\beta}, \sigma)$ that, conditional on $(\mathbf{t}, \mathbf{X})$, is independent of the other model variables, specifically:

$$
\begin{aligned}
f_{\boldsymbol{\beta},\sigma}\big(\boldsymbol{\beta}, \sigma \mid \boldsymbol{\mathcal{D}}, \mathbf{g}, \mathbf{t}, \boldsymbol{\theta}, \kappa\big) &\propto \prod_{i=1}^{n} f_{g,r,t,\mathbf{v},\mathbf{y},\boldsymbol{\beta},\sigma}(g_i, r_i, t_i, \mathbf{v}_i, \mathbf{y}_i, \boldsymbol{\beta}, \sigma \mid \mathbf{x}_i, \boldsymbol{\theta}, \kappa) \\
&= \prod_{i=1}^{n} \Big[f_g(g_i \mid \mathbf{x}_i, \boldsymbol{\theta}) f_r(r_i \mid \mathbf{x}_i) f_t(t_i \mid \mathbf{x}_i, \boldsymbol{\beta}, \sigma) \tilde{f}_{\mathbf{v},\mathbf{y}}(\mathbf{v}_i, \mathbf{y}_i \mid g_i, r_i, t_i, \mathbf{x}_i, \kappa) \Big] \\
&\quad \times \pi(\boldsymbol{\beta}, \sigma \mid \tau_\beta, \lambda) \\
&\propto \prod_{i=1}^{n} \Big[f_t(t_i \mid \mathbf{x}_i, \boldsymbol{\beta}, \sigma) \Big] \pi(\boldsymbol{\beta} \mid \tau_\beta)\ \pi_\sigma(\sigma \mid \lambda) \\
&= \mathcal{L}(\boldsymbol{\beta}, \sigma \mid \mathbf{t}, \mathbf{X})\ \pi(\boldsymbol{\beta} \mid \tau_\beta)\ \pi(\sigma \mid \lambda) \\
&\propto f_{\boldsymbol{\beta},\sigma}(\boldsymbol{\beta}, \sigma \mid \mathbf{t}, \mathbf{X})
\end{aligned}
\tag{S-40}
$$

where $\mathcal{L}(\boldsymbol{\beta}, \sigma \mid \mathbf{t}, \mathbf{X})$ is the complete-data likelihood, so that $f_{\boldsymbol{\beta},\sigma}(\boldsymbol{\beta}, \sigma \mid \mathbf{t}, \mathbf{X})$ is the complete-data posterior of $(\boldsymbol{\beta}, \sigma)$. The specific form of this posterior follows from the distributional assumption on the transition times t_i, which is implied by the distribution of the AFT residuals ϵ_i in model (4). As an example, we consider a Weibull model. Then the residuals are extreme value distributed with $f_\epsilon(\epsilon_i) = \exp(\epsilon_i - \exp(\epsilon_i))$ so that, by a change of variable, it follows that t_i has a Weibull density,

$$
f_t(t_i \mid \boldsymbol{\beta}, \sigma, \mathbf{x}_i) = \frac{\eta}{\gamma_i} \left(\frac{t_i}{\gamma_i} \right)^{(\eta-1)} \exp\left(- \left(\frac{t_i}{\gamma_i} \right)^{\eta} \right), \tag{S-41}
$$

where $\eta = \sigma^{-1} > 0$ and $\gamma_i = \exp(\mathbf{x}_i^T \boldsymbol{\beta})$. The log of the posterior (S-40) with priors as defined in Section 3.4 is then proportional to

$$
n \log(\eta) + \sum_{i=1}^{n} \left[(\eta - 1) \log(t_i) - \eta \log(\gamma_i) - \left(\frac{t_i}{\gamma_i} \right)^{\eta} \right] - \frac{1}{2} \left[\sum_{j=1}^{p} \left(\frac{\beta_j^2}{\tau_\beta} \right) + \frac{\eta^{-2}}{\lambda} \right]. \tag{S-42}
$$

In general, (S-40) does not follow a known distribution and, therefore, we use a Metropolis sampler to generate Markov Chain Monte Carlo (MCMC) samples. The Metropolis sampler applies a multivariate normal proposal (jumping) distribution that is centred at the previous draw $(\boldsymbol{\beta}^{(k)}, \sigma^{(k)})$ and has a diagonal variance-covariance matrix Σ chosen by the user. The proposal variance is a tuning parameter that needs to be calibrated such that the proposed jumps have an acceptance probability of approximately 23% (Gelman et al., 2013).

A.2.5 Updating the parameters of g_i

Gibbs sampler step (13) asks to obtain repeated draws from the full conditional distribution of $\boldsymbol{\theta}$ as follows:

$$\begin{aligned}
f_{\boldsymbol{\theta}}(\boldsymbol{\theta} \mid \boldsymbol{\mathcal{D}}, \mathbf{g}, \mathbf{t}, \boldsymbol{\beta}, \sigma, \kappa) &\propto \prod_{i=1}^{n} f_{g,r,t,\mathbf{v},\mathbf{y},\boldsymbol{\theta}}(g_i, r_i, t_i, \mathbf{v}_i, \mathbf{y}_i, \boldsymbol{\theta} \mid \mathbf{x}_i, \boldsymbol{\beta}, \sigma, \kappa) \\
&= \prod_{i=1}^{n} \left[f_g(g_i \mid \mathbf{x}_i, \boldsymbol{\theta}) f_r(r_i \mid \mathbf{x}_i) f_t(t_i \mid \mathbf{x}_i, \boldsymbol{\beta}, \sigma) \tilde{f}_{\mathbf{v},\mathbf{y}}(\mathbf{v}_i, \mathbf{y}_i \mid g_i, r_i, t_i, \mathbf{x}_i, \kappa) \right] \\
&\quad \times \pi(\boldsymbol{\theta} \mid \tau_\theta) \\
&= \prod_{i=1}^{n} \left[f_g(g_i \mid \mathbf{x}_i, \boldsymbol{\theta}) \right] \pi(\boldsymbol{\theta} \mid \tau_\theta) \\
&= \prod_{i=1}^{n} \left[\Phi(\mu_i)^{g_i} (1 - \Phi(\mu_i))^{(1-g_i)} \right] \pi(\boldsymbol{\theta} \mid \tau_\theta) \\
&= f_{\boldsymbol{\theta}}(\boldsymbol{\theta} \mid \mathbf{g}, \mathbf{X}),
\end{aligned} \tag{S-43}$$

which is the complete-data posterior of $\boldsymbol{\theta}$ given augmented $\mathbf{g}$. Sampling from this posterior could be achieved by a Metropolis step in similar manner as described for the parameters $(\boldsymbol{\beta}, \sigma)$ in Section A.2.4 of the Supplemental Material. However, `BayesPIM` achieves more efficient, conjugate normal updating through exploiting the latent variable formulation of the probit model. As described in Section 3.3, we define a new latent variable

$$w_i = \mu_i + \phi_i \quad , \phi_i \ \sim \ N(0,1) \tag{S-44}$$

and

$$g_i = \mathbb{1}_{\{w_i > 0\}}, \tag{S-45}$$

so that $\Pr(g_i = 1 \mid \mathbf{x}_i, \boldsymbol{\theta}) = \Pr(w_i > 0 \mid \mathbf{x}_i, \boldsymbol{\theta}) = \Phi(\mu_i)$. Under this model, equation (S-43) can be viewed as integral over latent w_i, i.e.

$$\begin{aligned}
f_{\boldsymbol{\theta}}(\boldsymbol{\theta} \mid \boldsymbol{\mathcal{D}}, \mathbf{g}, \mathbf{t}, \boldsymbol{\beta}, \sigma, \kappa) &\propto \prod_{i=1}^{n} \int_{-\infty}^{\infty} f_{g,r,t,\mathbf{v},w,\mathbf{y},\boldsymbol{\theta}}(g_i, r_i, t_i, \mathbf{v}_i, w_i, \mathbf{y}_i, \boldsymbol{\theta} \mid \mathbf{x}_i, \boldsymbol{\beta}, \sigma, \kappa) dw_i \\
&\propto \prod_{i=1}^{n} \left[\int_{-\infty}^{\infty} f_w(w_i \mid \mathbf{x}_i, \boldsymbol{\theta}) (\mathbb{1}_{\{w_i > 0\}})^{g_i} (\mathbb{1}_{\{w_i \le 0\}})^{(1-g_i)} dw_i \right] \pi(\boldsymbol{\theta} \mid \tau_\theta),
\end{aligned} \tag{S-46}$$

which directly equates to (S-43) because $f_w(w_i \mid \mathbf{x}_i, \boldsymbol{\theta})$ is a normal density with mean μ_i and variance 1. Now, instead of integrating over w_i analytically, an alternative is to use data augmentation of w_i in the Gibbs sampler, and subsequently sample $\boldsymbol{\theta}$ fully conditional (including w_i). This approach mirrors that of data augmentation for the Bayesian probit model (Albert and Chib, 1993), with the difference that in `BayesPIM` also g_i is partly latent and hence augmented through (10), while in the standard probit model the outcome is usually fully observed. Specifically, after updating g_i in Gibbs sampling step (10), we update the latent propensity w_i by drawing from truncated normal distributions

$$w_i \mid g_i, \mathbf{x}_i, \boldsymbol{\theta} \sim \begin{cases} N^+(w_i \mid \mu_i, 1) & \text{if } g_i = 1 \\ N^-(w_i \mid \mu_i, 1) & \text{if } g_i = 0 \end{cases} \tag{S-47}$$

where $N^+(w_i \mid \mu_i, 1)$ denotes the normal density with mean μ_i and variance 1 truncated $(0, \infty)$ and N^- denotes the same density truncated $(-\infty, 0)$. This result follows after observing that

g_i acts as constraint on w_i due to (S-45).

Subsequently, we use the fact that the parameters $\boldsymbol{\theta}$ only depend on the complete-data through $\mathbf{w} = (w_1, \ldots, w_n)^T$ and $\mathbf{X}$, i.e.

$$\begin{aligned} f_{\boldsymbol{\theta}}(\boldsymbol{\theta} \mid \boldsymbol{\mathcal{D}}, \mathbf{g}, \mathbf{t}, \mathbf{w}, \boldsymbol{\beta}, \sigma) &\propto f_{\boldsymbol{\theta}}(\boldsymbol{\theta} \mid \mathbf{w}, \mathbf{X}) \\ &= N\big(\boldsymbol{\theta} \mid \hat{\boldsymbol{\beta}}_g(\tau_\theta), (\mathbf{X}^T\mathbf{X} + \tau_\theta^{-1} I_p)^{-1}\big) \end{aligned} \tag{S-48}$$

where $\hat{\boldsymbol{\beta}}_g(\tau_\theta) = (\mathbf{X}^T\mathbf{X} + \tau_\theta^{-1} I_p)^{-1}\mathbf{X}^T\mathbf{w}$. Here, $f_{\boldsymbol{\theta}}(\boldsymbol{\theta} \mid \mathbf{w}, \mathbf{X})$ is the complete-data posterior of $\boldsymbol{\theta}$ conditional on augmented $\mathbf{w}$. Internally, `BayesPIM` augments w_i using (S-47) and then, instead of executing step (13), updates $\boldsymbol{\theta}$ from

$$\boldsymbol{\theta} \mid \mathbf{w}, \mathbf{X} \sim N\big(\hat{\boldsymbol{\beta}}_g(\tau_\theta), (\mathbf{X}^T\mathbf{X} + \tau_\theta^{-1} I_p)^{-1}\big). \tag{S-49}$$

A.3 Inference on the posterior predictive CIFs

A.3.1 Conditional posterior predictive CIF

We draw inference on the conditional CIF of the event time t for a non-prevalent individual with a fixed covariate value $\mathbf{x}_i = \tilde{\mathbf{x}}$

$$F_t(t \mid g = 0, \tilde{\mathbf{x}}, \boldsymbol{\beta}, \sigma). \tag{S-50}$$

To do so, we view $F_t(t \mid g = 0, \tilde{\mathbf{x}}, \boldsymbol{\beta}, \sigma)$ as a functional of the parameters $(\boldsymbol{\beta}, \sigma)$ with posterior distribution $f_{\boldsymbol{\beta},\sigma}(\boldsymbol{\beta}, \sigma \mid \boldsymbol{\mathcal{D}})$. The corresponding posterior (mean) predictive CIF at time t is defined as the posterior expectation

$$\begin{aligned} F_t(t \mid g = 0, \tilde{\mathbf{x}}, \boldsymbol{\mathcal{D}}) &= \mathbb{E}_{\boldsymbol{\beta},\sigma|\boldsymbol{\mathcal{D}}} \left[F_t(t \mid g = 0, \tilde{\mathbf{x}}, \boldsymbol{\beta}, \sigma) \right] & \text{(S-51)} \\ &= \int_0^\infty \int_{\Theta_t} F_t(t \mid g = 0, \tilde{\mathbf{x}}, \boldsymbol{\beta}, \sigma)\, f(\boldsymbol{\beta}, \sigma \mid \boldsymbol{\mathcal{D}})\, d\boldsymbol{\beta}\, d\sigma, & \text{(S-52)} \end{aligned}$$

where $\Theta_t = \{\boldsymbol{\beta} \in \mathbb{R}^p\}$. To approximate this expectation and perform inference, we use the retained post-burnin MCMC samples $(\boldsymbol{\beta}^{(k)}, \sigma^{(k)})$, $k = 1, \ldots, K$, from the marginal posterior $f_{\boldsymbol{\beta},\sigma}(\boldsymbol{\beta}, \sigma \mid \boldsymbol{\mathcal{D}})$ generated by the Gibbs sampler (10)–(14). We apply the push-forward transform

$$\nu_k(t, \tilde{\mathbf{x}}) = F_t(t \mid g = 0, \tilde{\mathbf{x}}, \boldsymbol{\beta}^{(k)}, \sigma^{(k)}), \tag{S-53}$$

which yields Monte Carlo samples from the posterior distribution of the CIF at time t for a non-prevalent individual with covariates $\tilde{\mathbf{x}}$. The posterior mean (Monte Carlo estimate of the posterior predictive CIF) is then

$$\frac{1}{K} \sum_{k=1}^{K} \nu_k(t, \tilde{\mathbf{x}}). \tag{S-54}$$

Furthermore, pointwise 95% credible intervals are obtained as the empirical 2.5% and 97.5% quantiles of the distribution $\{\nu_k(t, \tilde{\mathbf{x}})\}_{k=1}^K$.

A.3.2 Marginal posterior predictive CIF

Instead of conditioning on a specific combination of covariate values, we can also marginalize over the distribution of the covariates. This yields the marginal (or population-averaged) CIF at time t, defined by

$$F_t(t \mid g = 0, \boldsymbol{\beta}, \sigma) = \int_{\Theta_{\mathbf{x}}} F_t(t \mid g = 0, \mathbf{x}_t, \boldsymbol{\beta}, \sigma)\, f_{\mathbf{x}}(\mathbf{x}_t)\, d\mathbf{x}_t, \tag{S-55}$$

where $f_{\mathbf{x}}$ denotes the distribution of the covariates and $\Theta_{\mathbf{x}}$ its support. Taking $f_{\mathbf{x}}$ to be the empirical distribution of the observed covariates, this reduces to

$$F_t(t \mid g = 0, \boldsymbol{\beta}, \sigma) = \frac{1}{\sum_{i=1}^n (1 - g_i)} \sum_{i:g_i=0} F_t(t \mid g_i = 0, \mathbf{x}_i, \boldsymbol{\beta}, \sigma). \tag{S-56}$$

As in Section A.3.1, we treat $F_t(t \mid g = 0, \boldsymbol{\beta}, \sigma)$ as a functional of the parameters $(\boldsymbol{\beta}, \sigma)$ with marginal posterior distribution $f_{\boldsymbol{\beta},\sigma}(\boldsymbol{\beta}, \sigma \mid \boldsymbol{\mathcal{D}})$. The posterior (mean) predictive marginal CIF is then

$$F_t(t \mid g = 0, \boldsymbol{\mathcal{D}}) = \mathbb{E}_{\boldsymbol{\beta},\sigma|\boldsymbol{\mathcal{D}}} \left[F_t(t \mid g = 0, \boldsymbol{\beta}, \sigma) \right]. \tag{S-57}$$

In practice, with MCMC samples $(\boldsymbol{\beta}^{(k)}, \sigma^{(k)})$, $k = 1, \ldots, K$, we compute

$$\hat{\nu}_k(t) = \frac{1}{\sum_{i=1}^{n}(1-g_i)} \sum_{i:g_i=0} F_t\big(t \mid g_i = 0, \mathbf{x}_i, \boldsymbol{\beta}^{(k)}, \sigma^{(k)}\big). \tag{S-58}$$

The posterior (mean) predictive marginal CIF at time t is then estimated by

$$\widehat{F}_t(t \mid g = 0, \boldsymbol{\mathcal{D}}) = \frac{1}{K} \sum_{k=1}^{K} \hat{\nu}_k(t), \tag{S-59}$$

and pointwise 95% credible intervals are given by the empirical 2.5% and 97.5% quantiles of $\{\hat{\nu}_k(t)\}_{k=1}^{K}$.

A.3.3 Posterior predictive mixture CIF

Besides the CIF of t in the non-prevalent population, we draw inference on the mixture CIF. This is the CIF of t where we include prevalence $g = 1$ through a point probability mass at zero. This step requires defining a new variable (cf. Section 3.3)

$$t^* = (1 - g)t \tag{S-60}$$

so that $t^* = t$ in the non-prevalent case, and $t^* = 0$ in the prevalence case. The resulting conditional mixture CIF is

$$F_{t^*}(t \mid \tilde{\mathbf{x}}, \boldsymbol{\beta}, \sigma, \boldsymbol{\theta}) = \Phi(\tilde{\mathbf{x}}^T \boldsymbol{\theta}) + \big(1 - \Phi(\tilde{\mathbf{x}}^T \boldsymbol{\theta})\big) F_t(t \mid g = 0, \tilde{\mathbf{x}}, \boldsymbol{\beta}, \sigma). \tag{S-61}$$

The marginal mixture CIF over the covariate distribution $f_{\mathbf{x}}$ is

$$F_{t^*}(t \mid \boldsymbol{\beta}, \sigma, \boldsymbol{\theta}) = \int_{\Theta_{\mathbf{x}}} F_{t^*}(t \mid \mathbf{x}, \boldsymbol{\beta}, \sigma, \boldsymbol{\theta}) f_{\mathbf{x}}(\mathbf{x})\, d\mathbf{x}\,. \tag{S-62}$$

As in the previous sections, we consider $F_{t^*}(t \mid \mathbf{x}_i, \boldsymbol{\beta}, \sigma, \boldsymbol{\theta},)$ and $F_{t^*}(t \mid \boldsymbol{\beta}, \sigma, \boldsymbol{\theta},)$ as functionals of the posterior distribution $f_{\boldsymbol{\beta},\sigma,\boldsymbol{\theta}}(\boldsymbol{\beta}, \sigma, \boldsymbol{\theta} \mid \boldsymbol{\mathcal{D}})$. To estimate the posterior predictive conditional mixture CIF, we use the push-forward transform for the posterior samples $\boldsymbol{\beta}^{(k)}, \boldsymbol{\theta}^{(k)}, \sigma^{(k)}$, $k = 1, \ldots, K$, to obtain

$$\nu_k(t, \tilde{\mathbf{x}}) = F_{t^*}(t \mid \tilde{\mathbf{x}}, \boldsymbol{\beta}^{(k)}, \sigma^{(k)}, \boldsymbol{\theta}^{(k)}) \tag{S-63}$$

for a fixed $\tilde{\mathbf{x}}$. Subsequently, the posterior predictive conditional mixture CIF is obtained by the empirical mean of $\{\nu_k(t, \tilde{\mathbf{x}})\}_{k=1}^{K}$ and pointwise 95% credible intervals are obtained by the 2.5% and 97.5% quantiles of the empirical distribution $\{\nu_k(t, \tilde{\mathbf{x}})\}_{k=1}^{K}$. For the marginal mixture CIF, we first perform marginalization, as in Section A.3.2

$$\hat{\nu}_k(t) = \frac{1}{n} \sum_{i=1}^{n} F_{t^*}(t \mid \mathbf{x}_i, \boldsymbol{\beta}^{(k)}, \sigma^{(k)}, \boldsymbol{\theta}^{(k)}) \tag{S-64}$$

and do the inference as described above.

B Additional results from Simulation 1

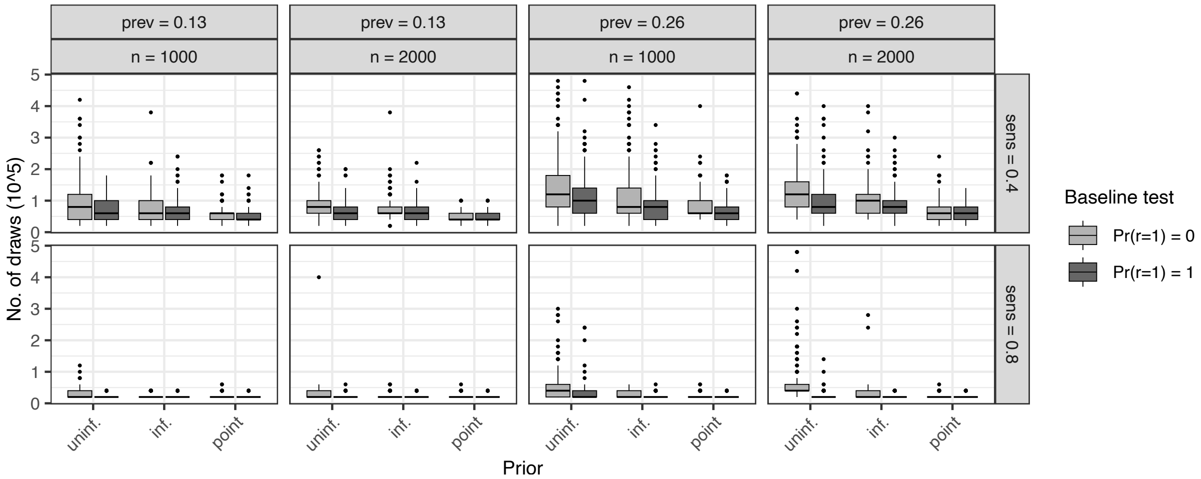

Figure B.1: Number of posterior draws until convergence including burn-in (scaled by 10^5) by simulation conditions. Convergence was evaluated every 2×10^4 draws. Abbreviations prev and sens denote, respectively, the prevalence probability $\Pr(g_i = 1)$ and the test sensitivity κ. The priors on the test sensitivity κ are either uninformative (uninf.), informative (inf.) or fixed at the true value (point).

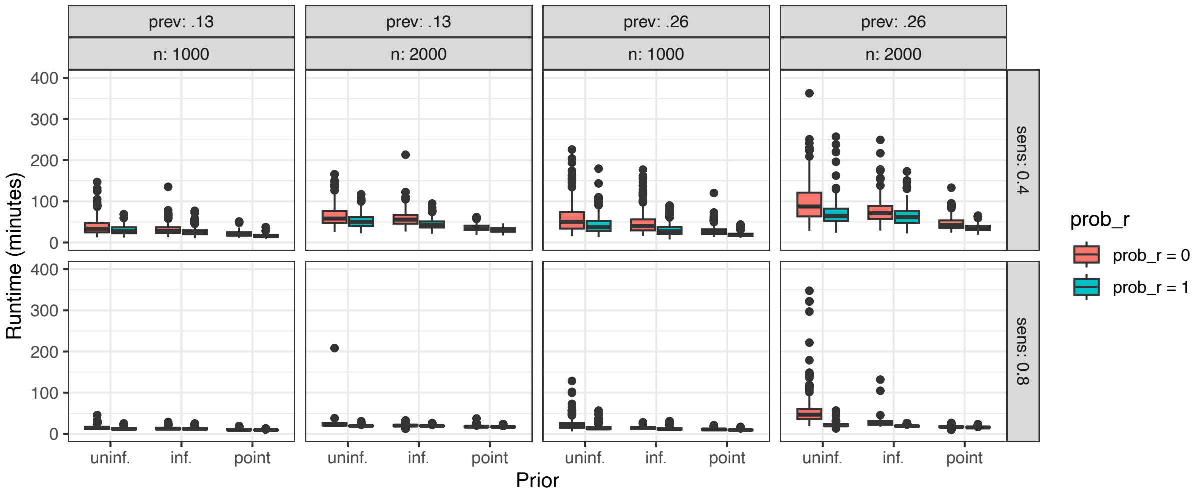

Figure B.2: Runtime of `BayesPIM` until convergence across simulation conditions (in minutes). Abbreviations prev and sens denote, respectively, the prevalence probability $\Pr(g_i = 1)$ and the test sensitivity κ. The priors on the test sensitivity κ are either uninformative (uninf.), informative (inf.) or fixed at the true value (point).

Table B.1: Linear regression of convergence time on design factors. Reference categories are given in the table footer.

Predictor	Coefficient	SE	t	p
Intercept	45.53	0.52	86.84	$< .001$
$n = 2000$	17.01	0.40	42.93	$< .001$
$\kappa = 0.8$ (sens)	-26.85	0.40	-67.77	$< .001$
Informative prior	-8.13	0.49	-16.75	$< .001$
Point prior on true value	-19.36	0.49	-39.88	$< .001$
$\Pr(r_i = 1) = 1$	-8.91	0.40	-22.47	$< .001$
$\Pr(g_i = 1) = 0.26$ (prev)	9.53	0.40	24.06	$< .001$

Notes: Unstandardized coefficients (B) from a linear regression predicting convergence time in minutes. Reference categories are $n = 1000$, test sensitivity (sens) $\kappa = 0.4$, uninformative prior, baseline test probability $\Pr(r_i = 1) = 0$, and prevalence probability (prev) $\Pr(g_i = 1) = 0.13$. Model fit: $R^2 = .49$, adjusted $R^2 = .49$, $F(6, 9587) = 1521$, $p < .001$, residual $SD = 19.41$.

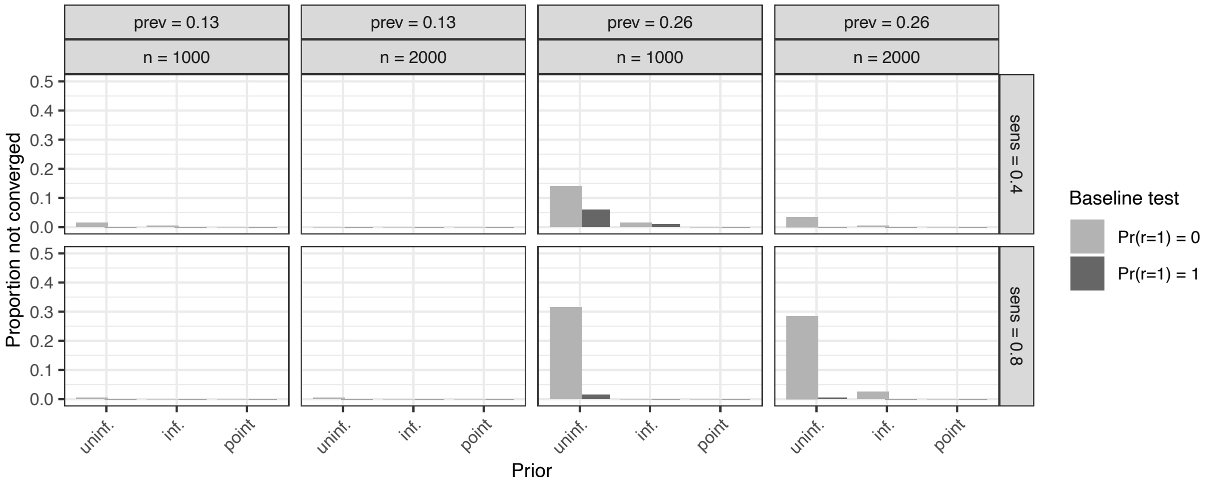

Figure B.3: Proportion of runs that did not converge until 5×10^5 Gibbs iterations, arranged by simulation conditions (200 runs per condition). Convergence was evaluated every 2×10^4 draws. Abbreviations prev and sens denote, respectively, the prevalence probability $\Pr(g_i = 1)$ and the test sensitivity κ. The priors on the test sensitivity κ are either uninformative (uninf.), informative (inf.) or fixed at the true value (point).

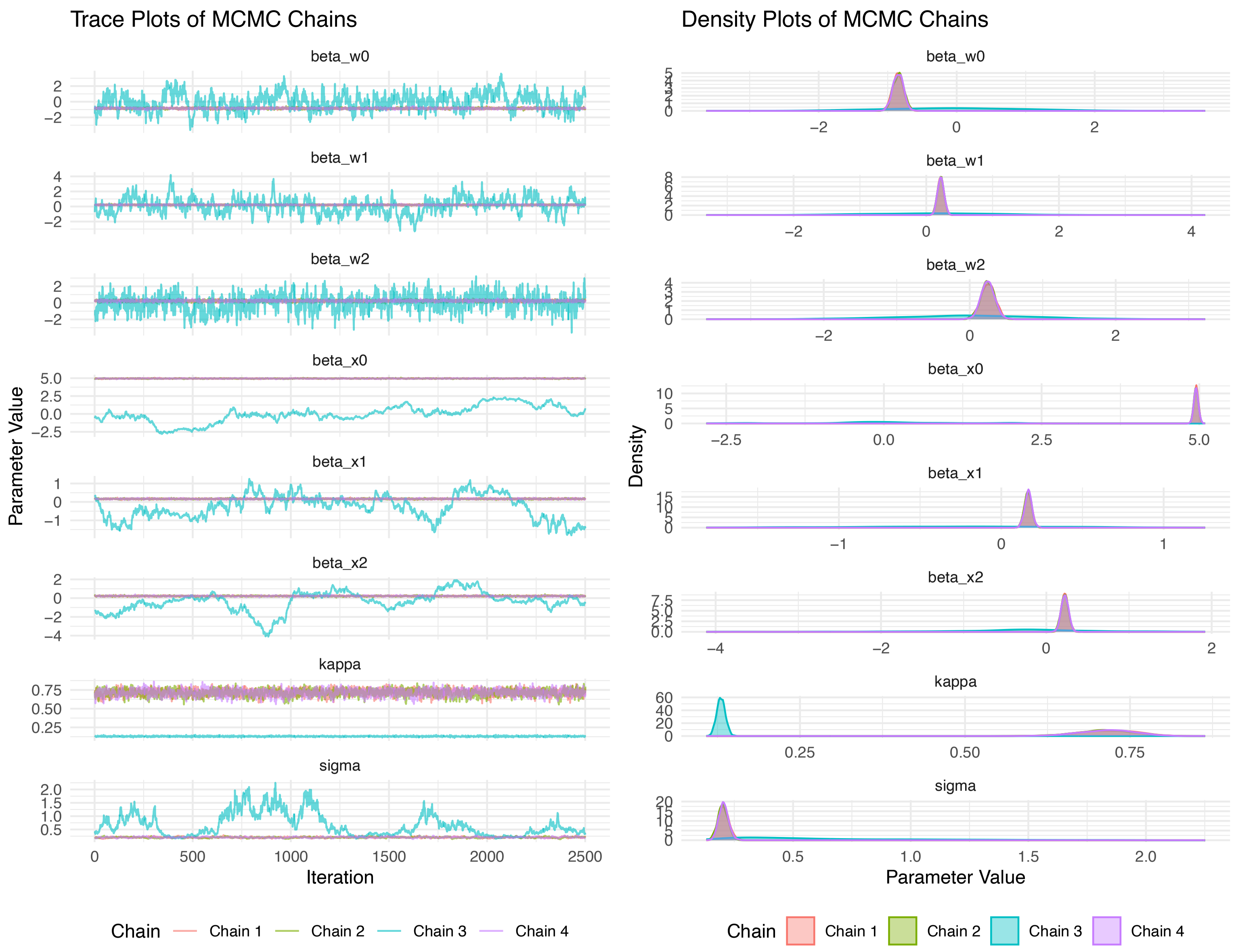

Figure B.4: Example of the MCMC chains resulting from a non-convergent run on a data set from Simulation 1 ($n_{sim} = 1000$, $\Pr(r_i = 1) = 1$, prev.=.26, $\kappa = .80$, uninformative prior on κ). The Gibbs sampler finds a local posterior mode in chain 2 centred arround an incorrect, very low κ estimate. Chains are thinned with an interlace rate of 200 between draws (5×10^5 draws in total, of which half are discarded for burn-in).

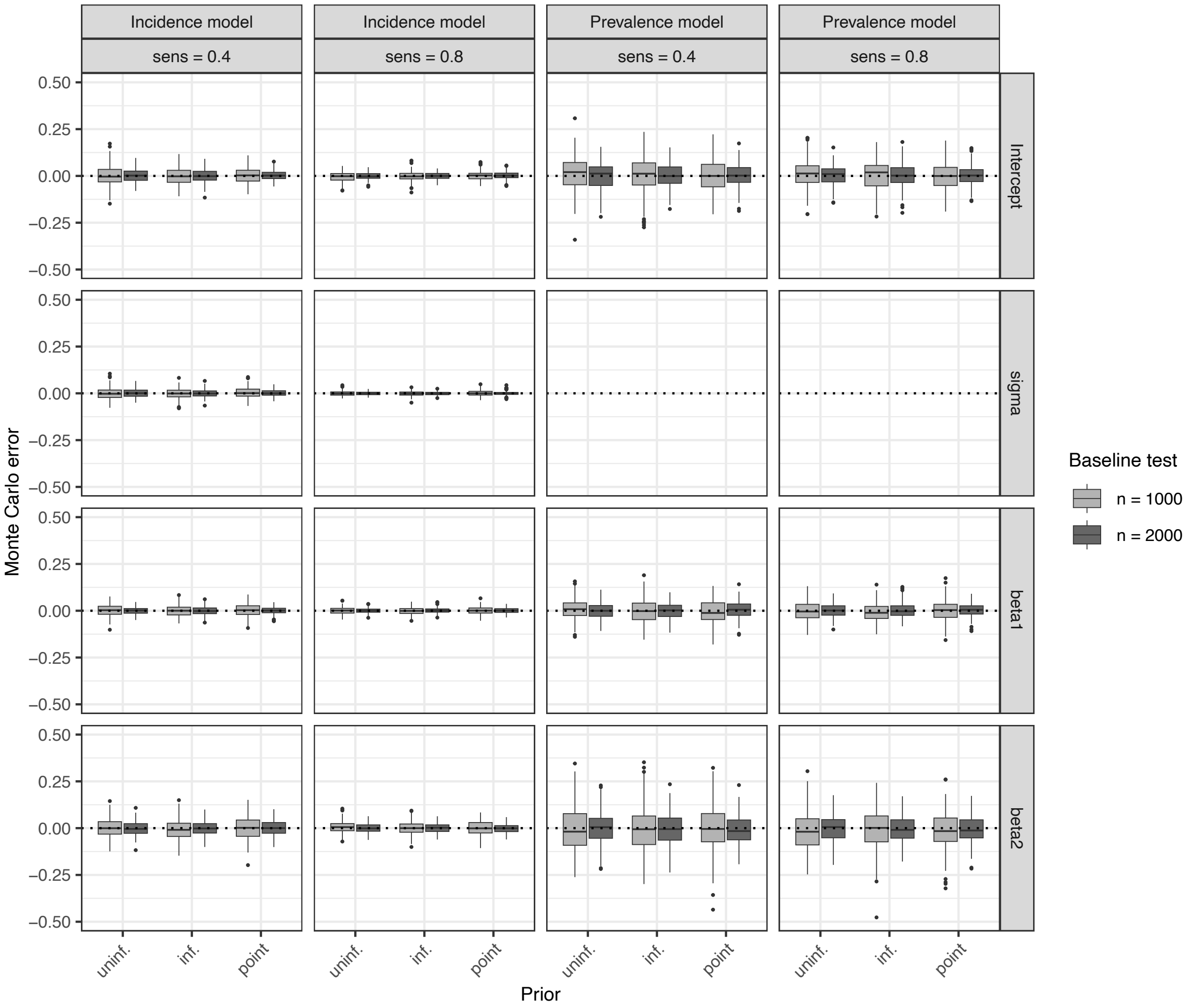

Figure B.5: Monte Carlo error of the model parameters, as indicated on the right, for both the incidence model (4) and the prevalence model (5) in the simulation condition: $\mathbf{Pr}(\boldsymbol{g_i} = \mathbf{1}) = \mathbf{0.13},\ \mathbf{Pr}(\boldsymbol{r_i} = \mathbf{1}) = \mathbf{1}$. Note that there is no σ parameter in the prevalence model and hence the corresponding panels are left blank. Row labels beta1 and beta2 refer to β_1 and β_2 in the incidence model and θ_1 and θ_2 in the prevalence model. Abbreviations prev and sens denote, respectively, the prevalence probability $\Pr(g_i = 1)$ and the test sensitivity κ. The priors on the test sensitivity κ are either uninformative (uninf.), informative (inf.) or fixed at the true value (point).

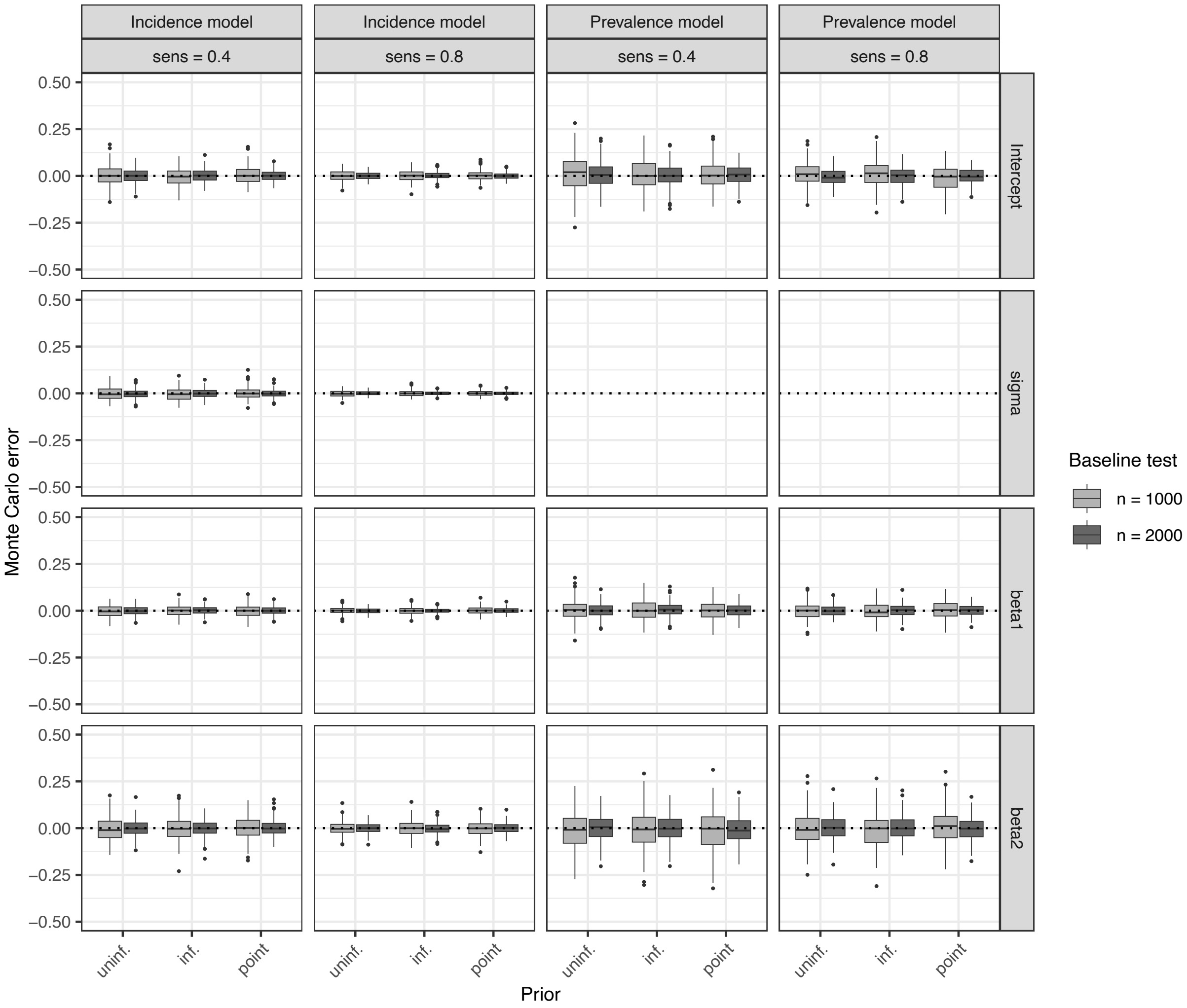

Figure B.6: Monte Carlo error of the model parameters, as indicated on the right, for both the incidence model (4) and the prevalence model (5) in the simulation condition: $\mathbf{Pr(g_i = 1) = 0.26,\ Pr(r_i = 1) = 1}$. Note that there is no σ parameter in the prevalence model and hence the corresponding panels are left blank. Row labels beta1 and beta2 refer to β_1 and β_2 in the incidence model and θ_1 and θ_2 in the prevalence model. Abbreviations prev and sens denote, respectively, the prevalence probability $\Pr(g_i = 1)$ and the test sensitivity κ. The priors on the test sensitivity κ are either uninformative (uninf.), informative (inf.) or fixed at the true value (point).

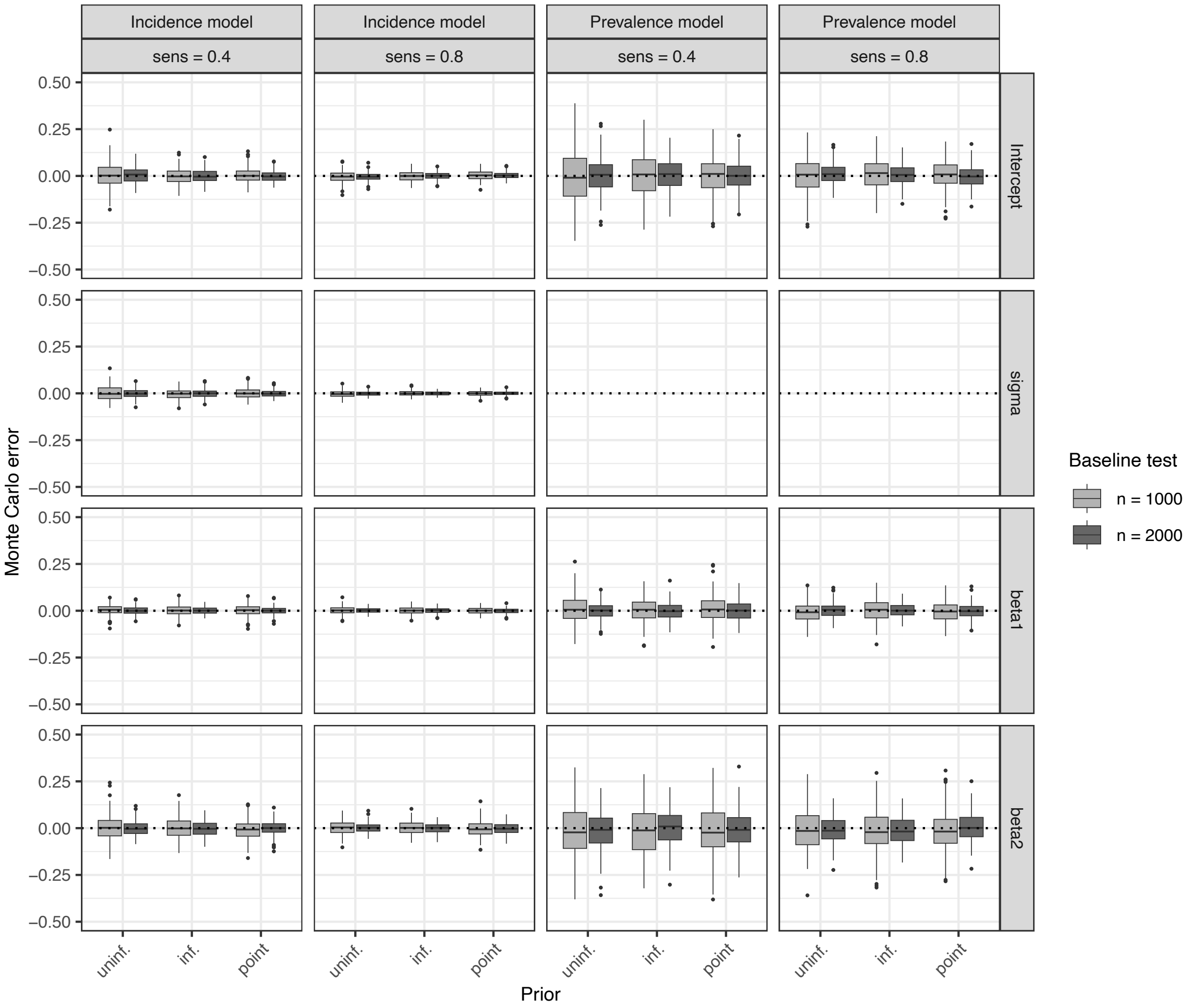

Figure B.7: Monte Carlo error of the model parameters, as indicated on the right, for both the incidence model (4) and the prevalence model (5) in the simulation condition: $\mathbf{Pr(g_i = 1) = 0.13,\ Pr(r_i = 1) = 0}$. Note that there is no σ parameter in the prevalence model and hence the corresponding panels are left blank. Row labels beta1 and beta2 refer to β_1 and β_2 in the incidence model and θ_1 and θ_2 in the prevalence model. Abbreviations prev and sens denote, respectively, the prevalence probability $\Pr(g_i = 1)$ and the test sensitivity κ. The priors on the test sensitivity κ are either uninformative (uninf.), informative (inf.) or fixed at the true value (point).

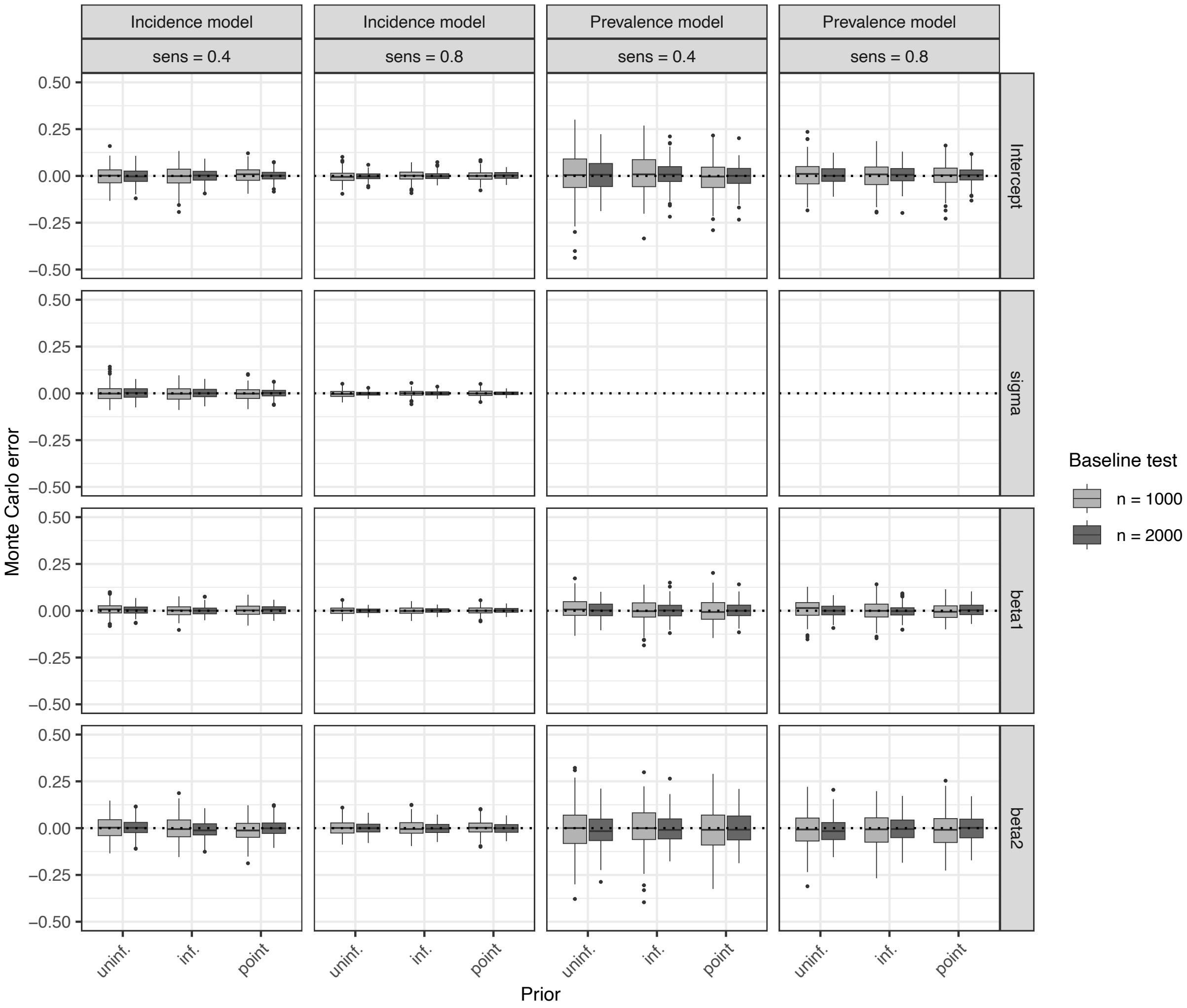

Figure B.8: Monte Carlo error of the model parameters, as indicated on the right, for both the incidence model (4) and the prevalence model (5) in the simulation condition: $\mathbf{Pr(g_i = 1) = 0.26,\ Pr(r_i = 1) = 0}$. Note that there is no σ parameter in the prevalence model and hence the corresponding panels are left blank. Row labels beta1 and beta2 refer to β_1 and β_2 in the incidence model and θ_1 and θ_2 in the prevalence model. Abbreviations prev and sens denote, respectively, the prevalence probability $\Pr(g_i = 1)$ and the test sensitivity κ. The priors on the test sensitivity κ are either uninformative (uninf.), informative (inf.) or fixed at the true value (point).

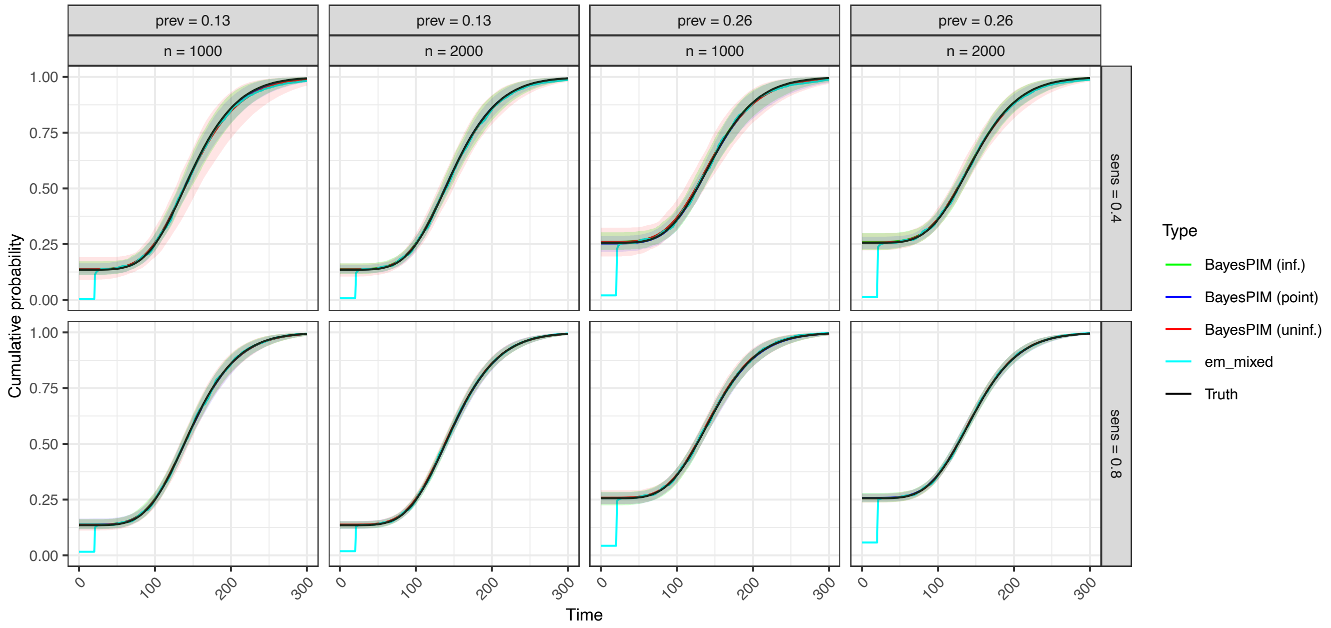

Figure B.9: Posterior median marginal mixture CIFs $F_{t^*}(t \mid \boldsymbol{\theta}, \boldsymbol{\beta}, \sigma)$, point-wise averaged over 200 Monte Carlo simulation runs with 95% quantiles shown as shaded regions. The condition $\mathbf{Pr}(\boldsymbol{r_i} = \mathbf{1}) = \mathbf{0}$ is shown.

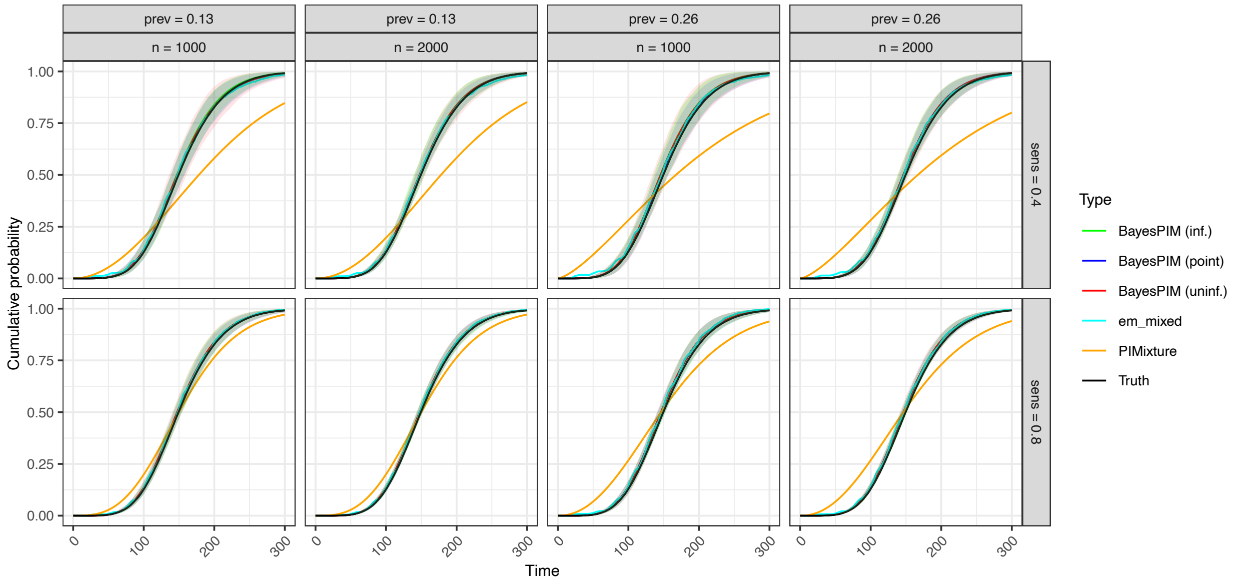

Figure B.10: Posterior median marginal CIFs for non-prevalent cases $F_t(t \mid g = 0, \boldsymbol{\beta}, \sigma)$, point-wise averaged over 200 Monte Carlo simulation runs with 95% quantiles shown as shaded regions. The condition $\mathbf{Pr}(\boldsymbol{r_i} = \mathbf{1}) = \mathbf{1}$ is shown. Lines of all models except `PIMixture` are overlapping.

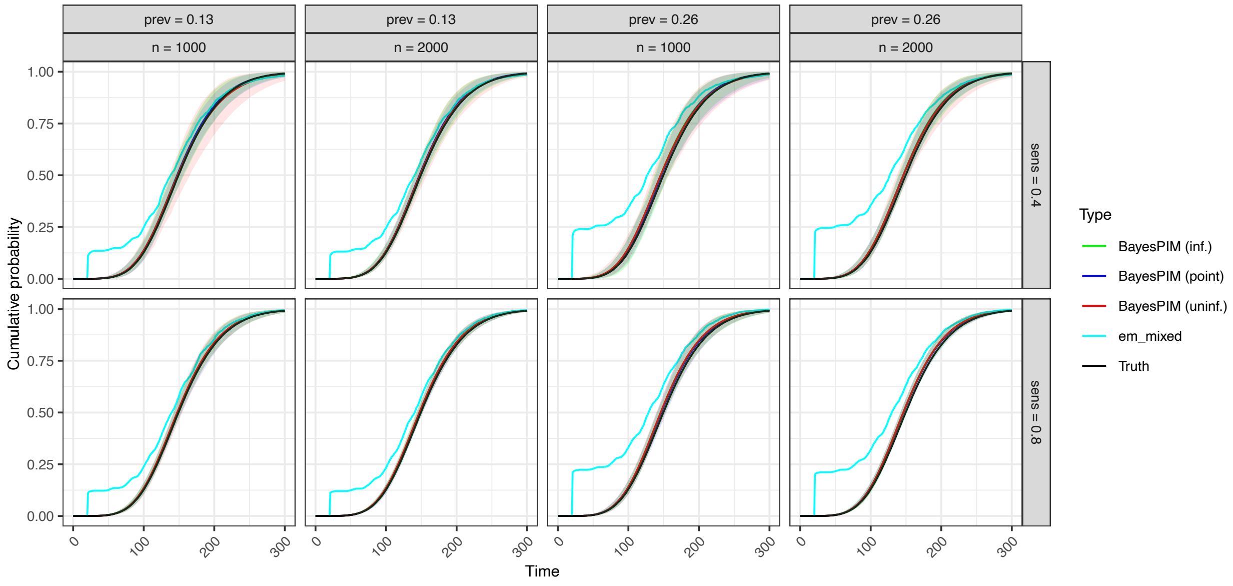

Figure B.11: Posterior median marginal CIFs for non-prevalent cases $F_t(t \mid g = 0, \boldsymbol{\beta}, \sigma)$, point-wise averaged over 200 Monte Carlo simulation runs with 95% quantiles shown as shaded regions. The condition $\mathbf{Pr}(\boldsymbol{r_i} = \mathbf{1}) = \mathbf{0}$ is shown. Lines of all models except `em_mixed` are overlapping.

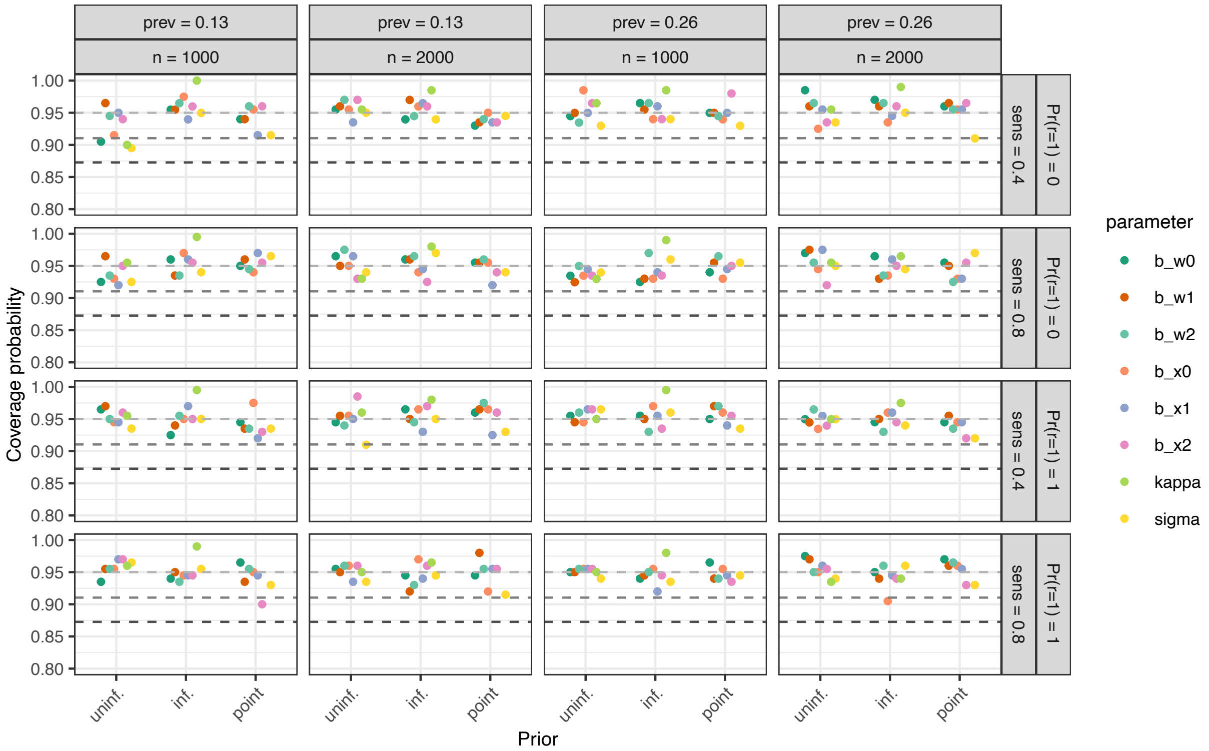

Figure B.12: Frequentist coverage probability of the Bayesian 95% posterior credible intervals for the 48 simulation conditions (estimated from 200 Monte Carlo data sets per condition by the proportion of intervals covering the true parameter value). The gray dotted lines in each panel denote, from top to bottom: (a) the nominal 95% level, (b) the value of a point estimate whose 95% confidence upper bound is equal to 95%, (c) the value of a point estimate whose 95% confidence upper bound is equal to 95% with a Bonferroni adjustment for 48 repeated tests.

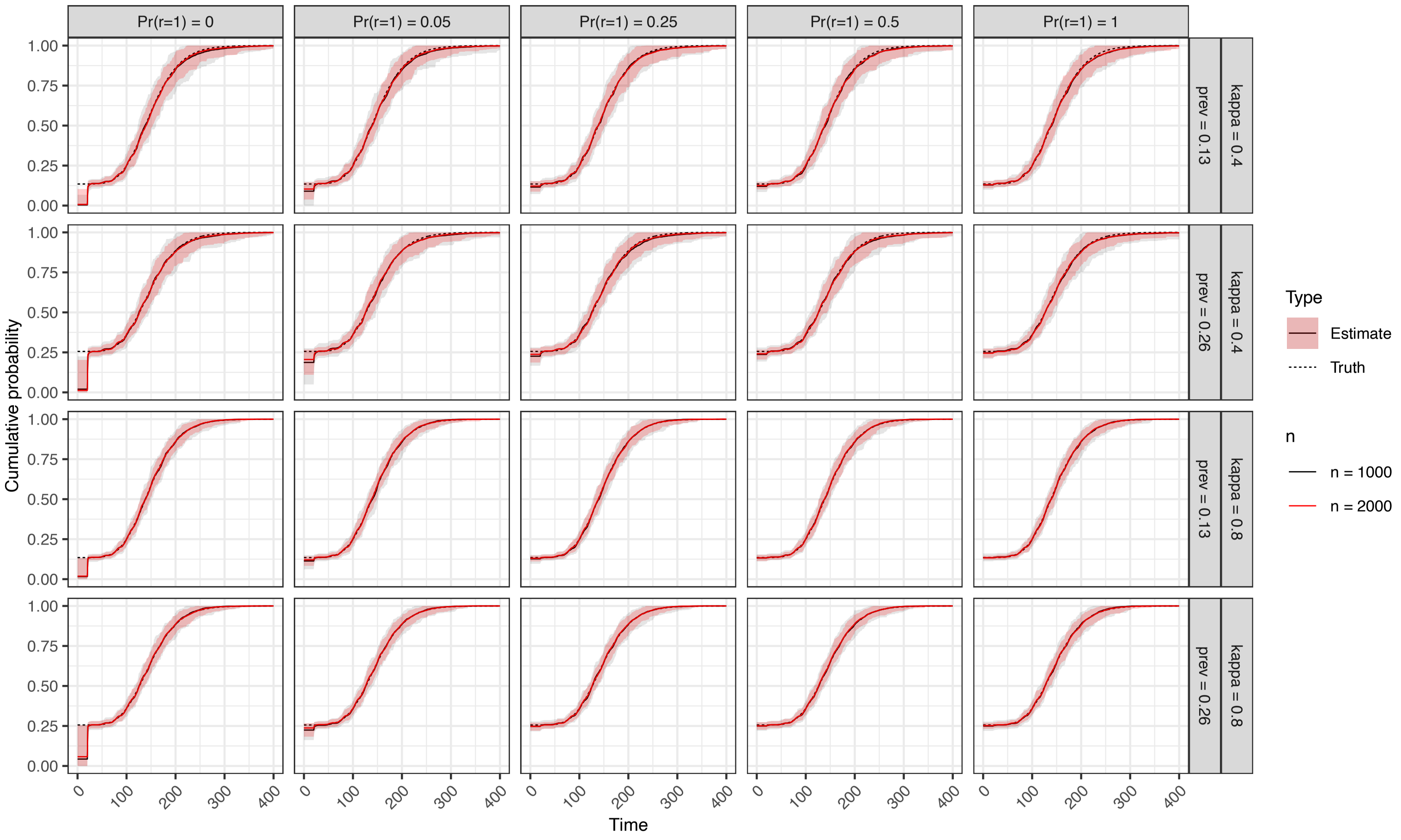

Figure B.13: Posterior median marginal mixture CIFs $F_{t^*}(t \mid \boldsymbol{\theta}, \boldsymbol{\beta}, \sigma)$ pointwise averaged over the estimates from 200 data sets. These are additional results on the `em_mixed` estimator. The same simulation conditions as in Simulation 1 are used, but a wider range of baseline test probabilities $\Pr(r_i = 1) \in \{0, 0.05, 0.25, 0.5, 1\}$ is reported. The results demonstrate that `em_mixed` was only approximately unbiased in the $\Pr(r_i = 1) = 1$ but the bias was negligible when a moderate number of tests was available (e.g. $\Pr(r_i = 1) = 0.5$).

C Additional details on the set-up of Simulation 2

C.1 Data generation by resampling the CRC EHR data

For Simulation 2, we here give details on the data generating process which resampled the CRC EHR to obtain realistic screening times and covariate distributions. Specifically, we explain how we resampled the CRC EHR to obtain new screening times from the observed screening times distribution and determined the event and right censoring times for each newly generated data point k. The re-sampled screening times distribution was compared with the observed screening times distribution on a number of benchmark statistics to assure that the simulated data resembled the CRC EHR (see Figure C.1).

Specifically, we followed these steps:

1. Set the true parameters of $(\boldsymbol{\beta}, \sigma, \boldsymbol{\theta})$ to the posterior median estimates from the application (Table 4, Section 6).

Then for each newly generated individual $k = 1, ..., n_{sim}$ with f_t the Weibull density, we generated the latent incidence time (t_k), prevalence status (g_k) and baseline test status (r_k with Bernoulli probability 0.93 set to that observed in the CRC EHR data). We proceeded as follows:

2. Randomly sample integer i' discretely uniform from $\{1, \ldots, n\}$
3. Set $\mathbf{x}_k = \mathbf{x}_{i'}$ (in words: set the sampled covariates to those of the i'-th individual in the CRC EHR data; covariates for the incidence and prevalence model are the same, hence $\mathbf{x}_k$ without index g or t)
4. Generate: $t_k \mid \mathbf{x}_k, \boldsymbol{\beta}, \sigma \sim f_t(t_k \mid \mathbf{x}_k, \boldsymbol{\beta}, \sigma)$
5. Generate: $g_k \mid \mathbf{x}_k, \boldsymbol{\theta} \sim f_g(g_k \mid \mathbf{x}_k, \boldsymbol{\theta})$
6. Generate: $r_k \sim \text{Bernoulli}(0.93)$

This procedure followed the hierarchical model described in Section 3.2. To obtain new screening times and run screening tests according to the hierarchical model, equations (2)–(3), we used the observed screening times $\mathbf{v}_{i'} = (v_{i'1}, v_{i'2}, \ldots, v_{i'c_{i'}})$ of the sampled unit i' as donor for new screening times. Specifically, we set $v_{k1} = 0$ and stopped screening, i.e. $y_{k1} = 1$ (positive baseline test), if $g_k = 1$ and $r_k = 1$ with probability κ. If $r_k = 0$, y_{k1} is missing, as described in Section 3.2. Else we set $y_{k1} = 0$ and, subsequently, for each $j = 2, \ldots c_{i'}$ we followed the following steps:

1. Set $v_{kj} := v_{i'j}$
2. Generate y_{kj} as defined by (3)

The procedure was stopped if $y_{kj} = 1$ such that $\mathbf{v}_k = (v_{k1}, \ldots, v_{kj})$. If the process was not stopped until $v_{k,c_{i'}-1}$, its continuation depended on $y_{i'c_{i'}}$ indicating whether individual i' was not right censored at $v_{i'c_{i'}-1}$. If $y_{i'c_{i'}} = 0$, i' was right censored, we set $v_{kc_{i'}} := v_{i'c_{i'}} = \infty$, to indicate that also $\mathbf{v}_k$ was right censored. However, when $y_{i'c_{i'}} = 1$, $\mathbf{v}_{i'}$ was not right censored because an event was observed and screening stopped at $v_{i'c_{i'}} < \infty$ due to (3). As a consequence, no further screening times beyond $v_{i'c_{i'}}$ were available as donors but for unit k screening should continue if $y_{k,c_{i'}-1} = 0$. To generate new times after $v_{i'c_{i'}}$ (if $y_{i'c_{i'}} = 1$) we, therefore, approximated the unobserved distribution of screening times that would have occurred if, counter to the fact, after the first $\mathbf{v}_{i'}$ times the series had not been stopped due to an observed event for individual i'. To do so, we resampled time difference scores $d_{ij} = v_{ij} - v_{ij-1}$ from the empirical distribution of all observed difference scores in the group that was not right censored (all individuals with

$y_{ic_i} = 1$). Samples $\tilde{d}$ from this distribution were obtained by calculating d_{ij} for all i and j in the data for which $y_{ic_i} = 1$ and then randomly selecting, with replacement, one $\tilde{d}$ from the pool of all d_{ij}. Call the set of all difference scores D. Then, the procedure for generating screening times was continued as follows (if $y_{i'c_{i'}} = 1$):

- Set $v_{kc_{i'}} := v_{i'c_{i'}}$ (the final known screening time at which i' had a positive test can still be used as donor for k)
- Generate $y_{kc_{i'}}$ as defined by (3)
- Set $v_{k,c_{i'}+1} := v_{kc_{i'}} + \tilde{d}$, where $\tilde{d}$ is drawn uniformly from set D
- Generate $y_{k,c_{i'}+1}$ as defined by (3)
- Set $v_{k,c_{i'}+2} := v_{k,c_{i'}+1} + \tilde{d}$, where $\tilde{d}$ is newly drawn uniformly from set D
- Generate $y_{k,c_{i'}+2}$ as defined by (3)
- ...

This process is continued until stopping due to $y_{kj} = 1$ or right censoring which happens as defined by the hierarchical model (Section 3.2) if $v_{kc_{i'}+j} > s_k$, where s_k is the latent time of right censoring. We explain below how we obtained right censoring time s_k from its approximated empirical distribution.

The imputation procedure used to generate screening times $v_{kc_{i'}+j}$, $j \geq 1$, was based on assumptions that we, here, make explicit. We assumed (a) that the observed time differences $d_{ij} = v_{ij} - v_{ij-1}$ between the observed screening visits v_{ij} were exchangeable with those between the unobserved screening times (i.e. the screening process would have continued as it was observed into the future), (b) that the difference scores d_{ij} were exchangeable between units i and (c) the ordering implied by index j did not matter for future screening moments. Assumption (b) was needed to avoid very similar distances between successive screening moments (e.g., if $\mathbf{v}$ was short such as $\mathbf{v} = (0, 3)$ using only the observed difference $d_i = 3$ to impute subsequent screening times would not have appropriately represented the empirically observed variation in timely distance between screening moments on other units).

We now return to the question of how to sample the time of right censoring s_k from the empirical right censoring distribution. Our goal is to use $s_{i'}$ as a donor but we note that $s_{i'}$ is unobserved in all cases and should not be confused with $v_{i'c_{i'}}$. Time $v_{i'c_{i'}}$ is the finite time point at which the event occurred if $y_{i'c_{i'}} = 1$ and $v_{i'c_{i'}} = \infty$ in case of right censoring ($y_{i'c_{i'}} = 0$). Then $v_{i',c_{i'}-1}$ with $v_{i',c_{i'}-1} < s_{i'}$ denotes the last follow-up moment (without event), but not the time of right censoring. Hence, the last screening moment is a lower bound for $s_{i'}$. Now we note that right censoring occurs if the time of right censoring $s_{i'}$ is sooner than the time of the next screening moment. Therefore, if $y_{i'c_{i'}} = 0$, right censoring occurs when $v_{i',c_{i'}-1} < s_{i'} \leq v_{i',c_{i'}-1} + \tilde{v}_{i'}$, where $\tilde{v}_{i'}$ denotes the unknown time until the next screening moment. Hence, an upper bound for $s_{i'}$ is found, namely $v_{i',c_{i'}-1} + \tilde{v}_{i'}$. Now, returning to the full CRC EHR sample $i = 1, \ldots, n$ with $y_{i,c_i} = 0$, if $\tilde{v}_i$ was known for all i in the CRC EHR data, which it is not, the distribution of s_i could be estimated by treating the time as interval-censored between the last observed times v_{i,c_i-1} and the missing $v_{i,c_i-1} + \tilde{v}_i$. Our strategy for estimating the distribution of s_i therefore was defined as follows. For all i for which $y_{ic_i} = 0$, set

1. $l_i := v_{i,c_i-1}$
2. $r_i := l_i + \tilde{v}_i$, where $\tilde{v}_i$ is sampled uniformly from set D

Subsequently, we estimated $\hat{F}_s(s)$, the empirical CDF of s_i, through interval-censored nonparametric maximum likelihood using the Turnbull estimator (Turnbull, 1976), where the intervals were given by $(l_i, r_i]$. This procedure was repeated $b = 1, \ldots, 1000$ times on bootstrapped samples from the CRC EHR data to reflect the uncertainty in the Turnbull estimator as well as the repeated imputations $\tilde{v}_i$ (Figure C.1). For each bootstrapped data set we thusly obtained one $\hat{F}_s^{(b)}(s)$. To draw one right censoring time s_k from this approximated distribution, we first randomly selected one $\hat{F}_s^{(b)}(s)$ and subsequently used the inverse sampling method to obtain s_k under the constraint that $s_k > v_{kc_{i'}}$. This is achieved by drawing from the truncated distribution $\hat{F}_s^{(b)}$ subject to truncation rule $s_k > v_{kc_{i'}}$. Technically, we reject draws violating this rule and repeat the draw.

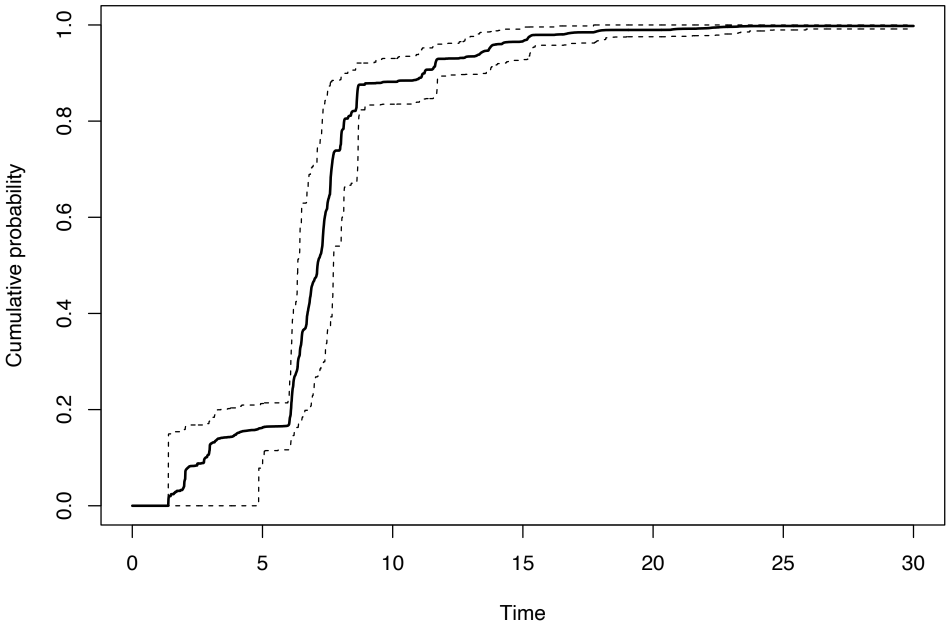

Figure C.1: Bootstrapped approximated empirical CDF of s_i in the CRC EHR, the time of right censoring in the data. Solid line represents point-wise averages across bootstrapped samples with the interval representing point-wise 95% confidence intervals.

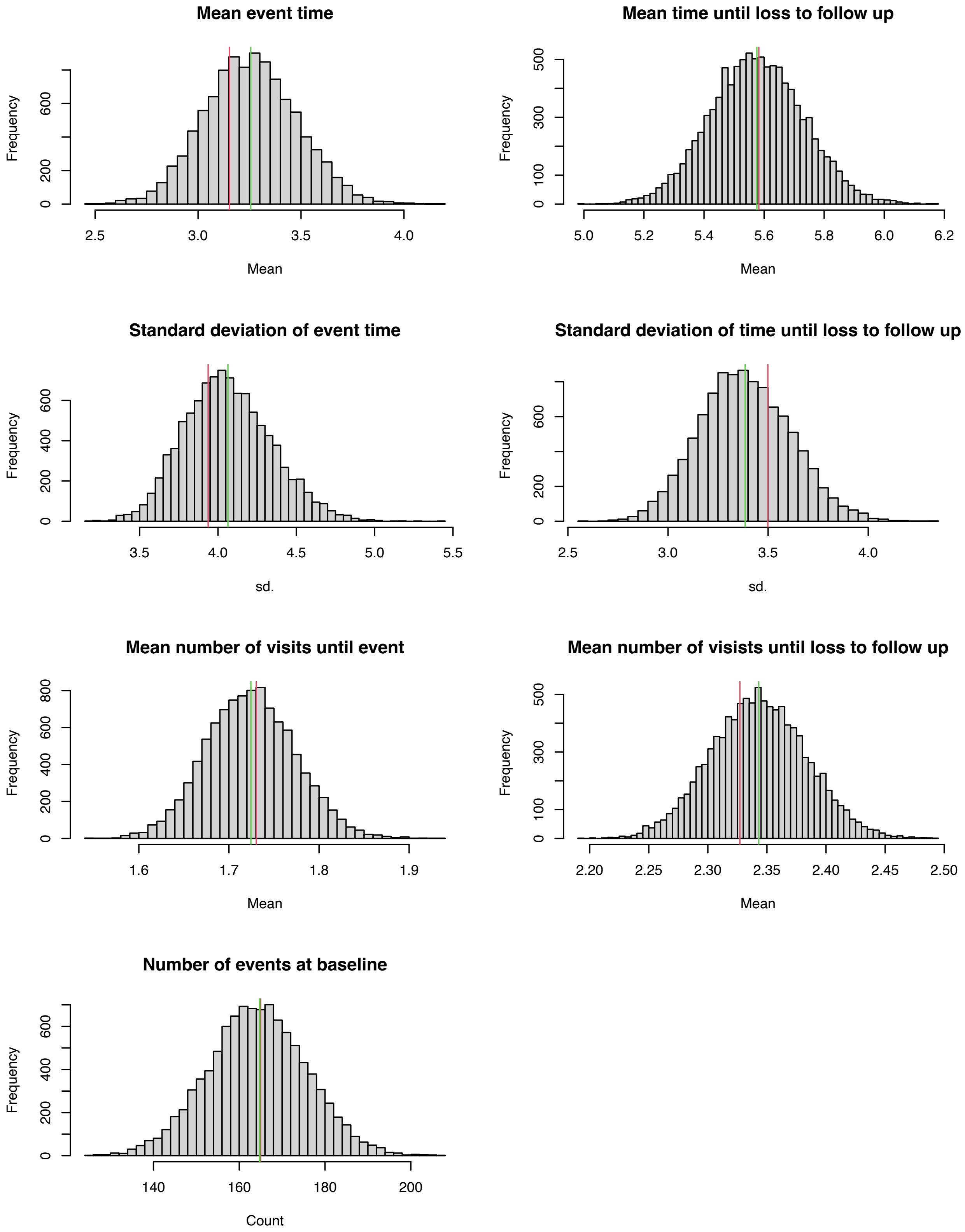

Figure C.2: Sampling distributions (Simulation 2) of statistics calculated on 10,000 samples of simulated censoring times $\{\mathbf{v}_k\}_{k=1}^{n}$ where $n_{sim} = 810$ (sample size of the CRC EHR data). Green shows the mean of the sampling distribution and red shows the observed value calculated on the CRC EHR data. These comparisons demonstrate that the simulated screening times distribution was similar to the observed screening times distribution in the CRC EHR.

C.2 Generation of screening times under extended right censoring

In Simulation 2, we considered an extended right censoring distribution as an addition to the empirical approximation $\hat{F}_s(s)$ to the right censoring distribution in the CRC EHR data described in Section C.1. The extended right censoring distribution was obtained through adding an offset to s_i, i.e.

$$s_i^{ext} := s_i + \Delta, \tag{S-65}$$

where $\Delta = 10$ years which shifted $\hat{F}_s(s_i)$ to the right. This procedure generally led to longer potential follow up and more screening moments for every k.

The process of generating screening times was similar to that described in Section C.1 with two differences. First, instead of sampling right censoring times from $\hat{F}_s(s)$ under constraint $s_k > v_{kc_{i'}}$, we now sample from the shifted (extended) distribution $\hat{F}_s^{ext}(s_i)$ under the constraint $s_k^{ext} > v_{kc_{i'}}$. This distribution was obtained with the same procedure as described in Section C.1 with the difference that the $(l_i, r_i]$ interval bounding s_i was shifted $(l_i + \Delta, r_i + \Delta]$ before applying the Turnbull (1976) estimator. Second, individuals i experiencing an event ($y_{ic_i} = 1$) acted as donors for $\mathbf{v}_i$ with times and (extended) right censoring time augmented as described in Section C.1. However, right censored individuals i ($y_{ic_i} = 0$) lacked visit times after their time of right censoring until the extended right censoring time $\Delta = 10$ years later. Therefore, also their visit times were augmented until extended right censoring in the same way as described in Section C.1. Note that this was not necessary under standard right censoring when their last moment of follow up could naturally be used as the last moment before right censoring.

D Additional results from Simulation 2 (CRC EHR)

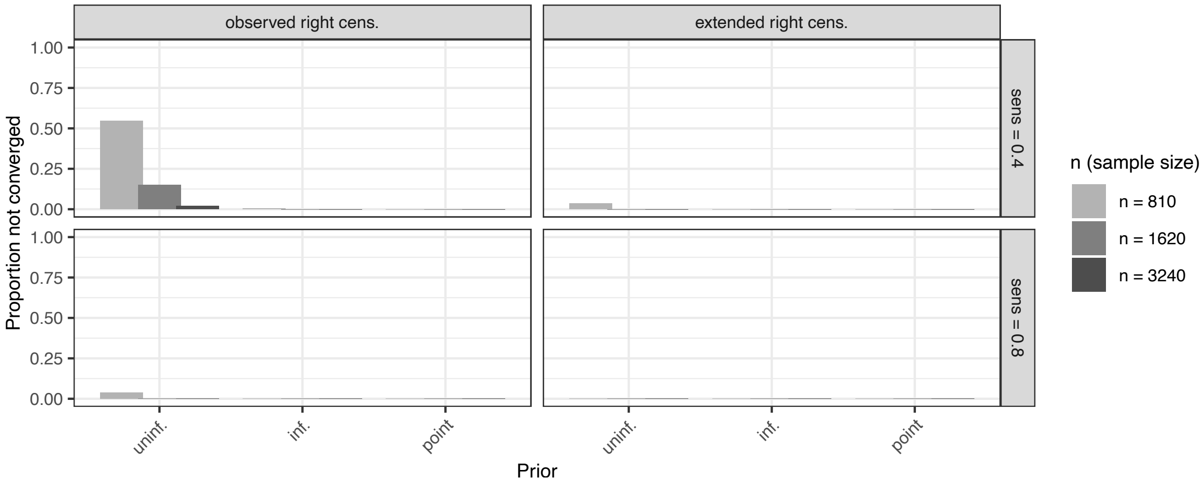

Figure D.1: Proportion of runs that did not converge per simulation condition. Convergence was evaluated every 2×10^4 draws. Abbreviations prev and sens denote, respectively, the prevalence probability $\Pr(g_i = 1)$ and the test sensitivity κ. The priors on the test sensitivity κ are either uninformative (uninf.), informative (inf.) or fixed at the true value (point).

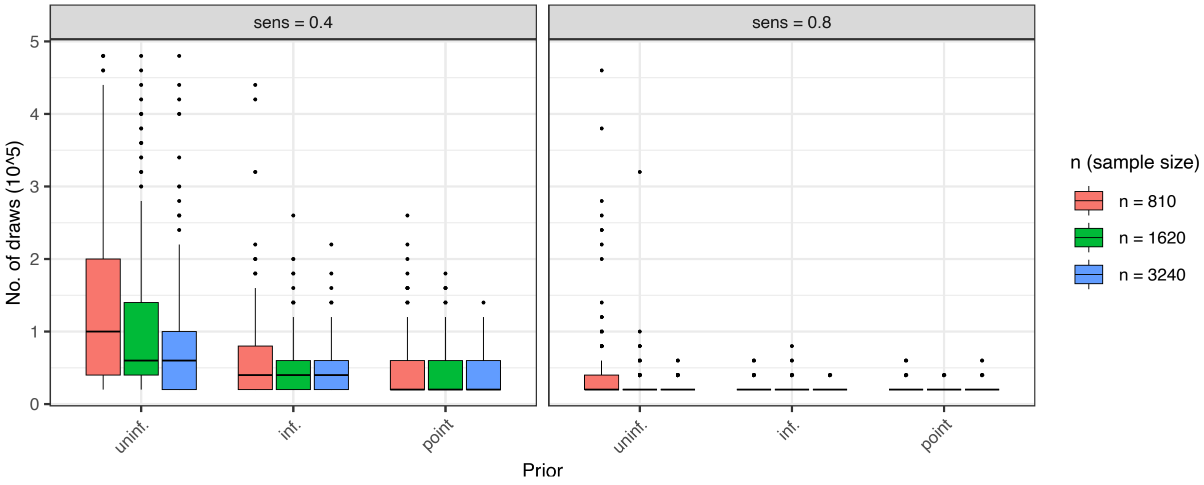

Figure D.2: Number of posterior draws until convergence including burn-in (scaled by 10^5) by simulation conditions. Convergence was evaluated every 2×10^4 draws. Abbreviation sens denotes the test sensitivity κ. The priors on the test sensitivity κ are either uninformative (uninf.), informative (inf.) or fixed at the true value (point).

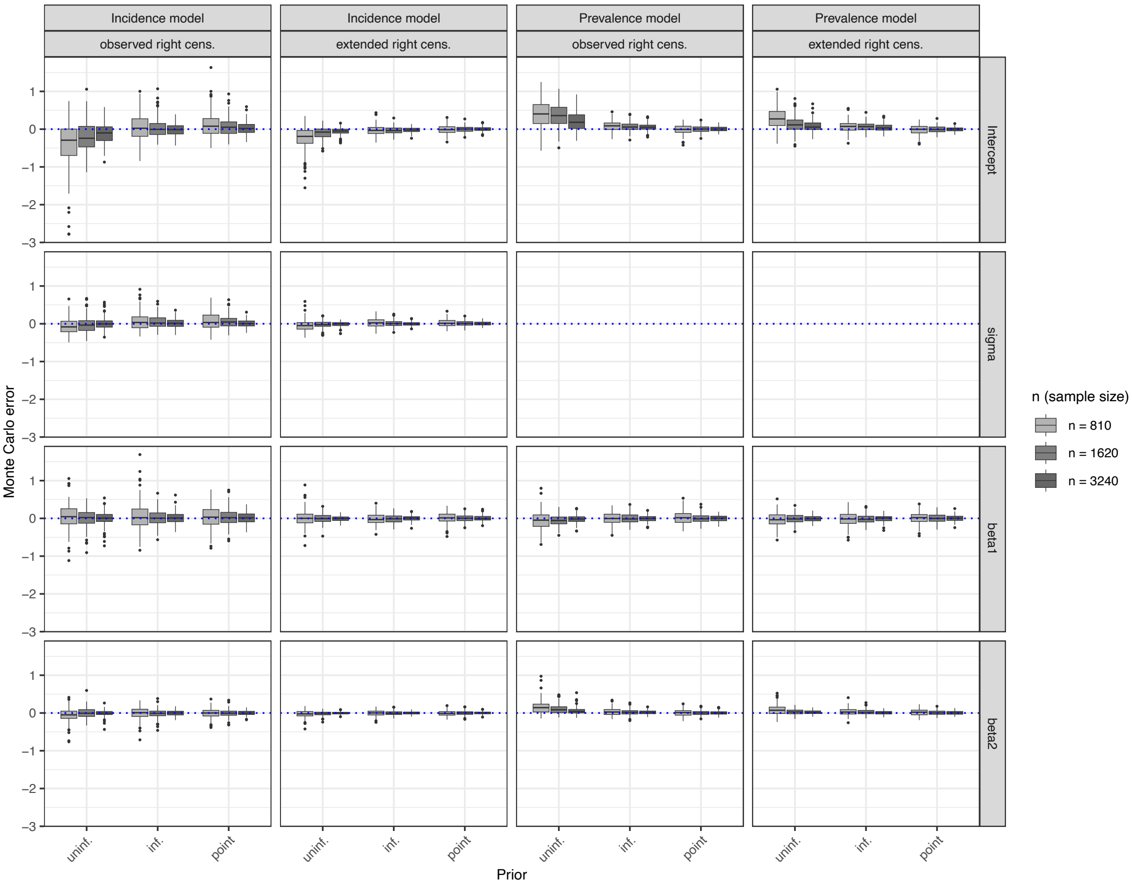

Figure D.3: Monte Carlo error of the model parameters (Simulation 2), as indicated on the right, for both the incidence model (4) and the prevalence model (5) in the simulation condition: $\boldsymbol{\kappa = 0.4}$. Note that there is no σ parameter in the prevalence model and hence the corresponding panels are left blank. Row labels beta1 and beta2 refer to β_1 and β_2 in the incidence model and θ_1 and θ_2 in the prevalence model. Abbreviation sens denotes the test sensitivity κ. The priors on the test sensitivity κ are either uninformative (uninf.), informative (inf.) or fixed at the true value (point).

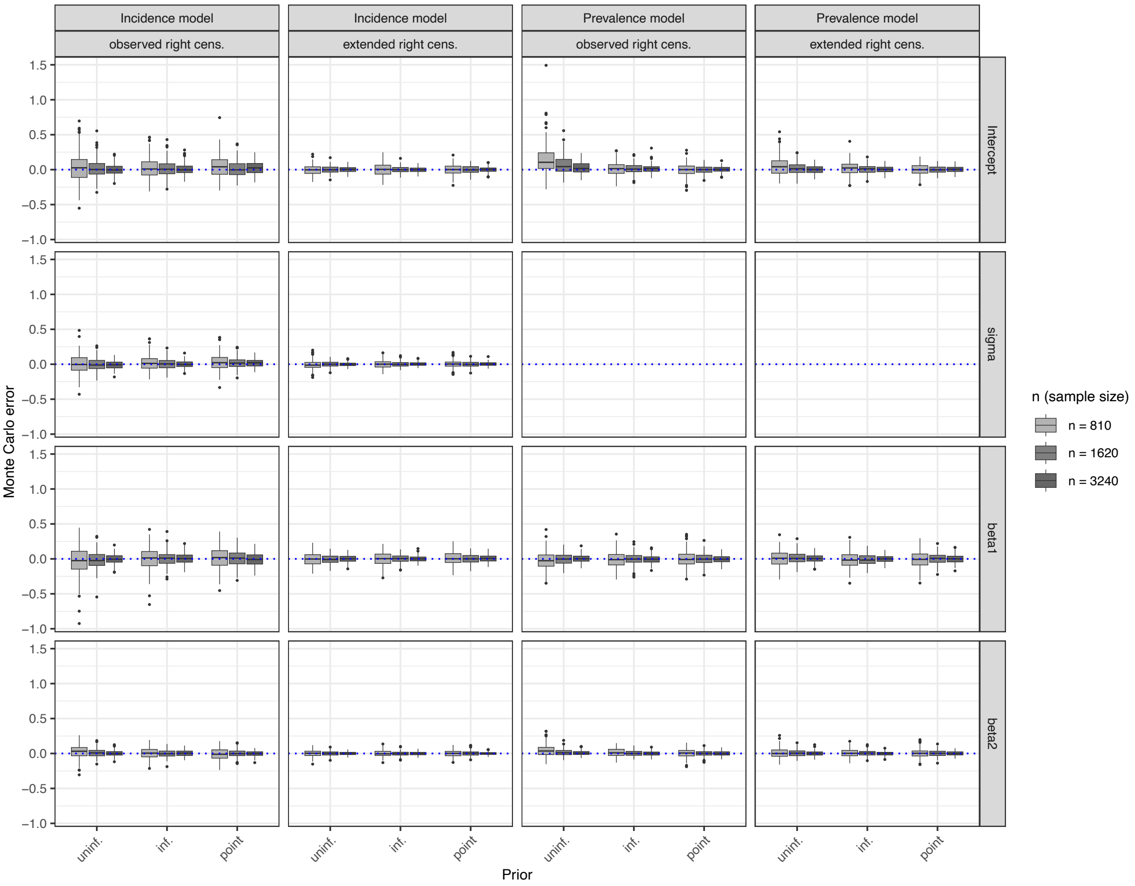

Figure D.4: Monte Carlo error of the model parameters (Simulation 2), as indicated on the right, for both the incidence model (4) and the prevalence model (5) in the simulation condition: $\boldsymbol{\kappa} = \mathbf{0.8}$. Note that there is no σ parameter in the prevalence model and hence the corresponding panels are left blank. Row labels beta1 and beta2 refer to β_1 and β_2 in the incidence model and θ_1 and θ_2 in the prevalence model. Abbreviation sens denotes the test sensitivity κ. The priors on the test sensitivity κ are either uninformative (uninf.), informative (inf.) or fixed at the true value (point).

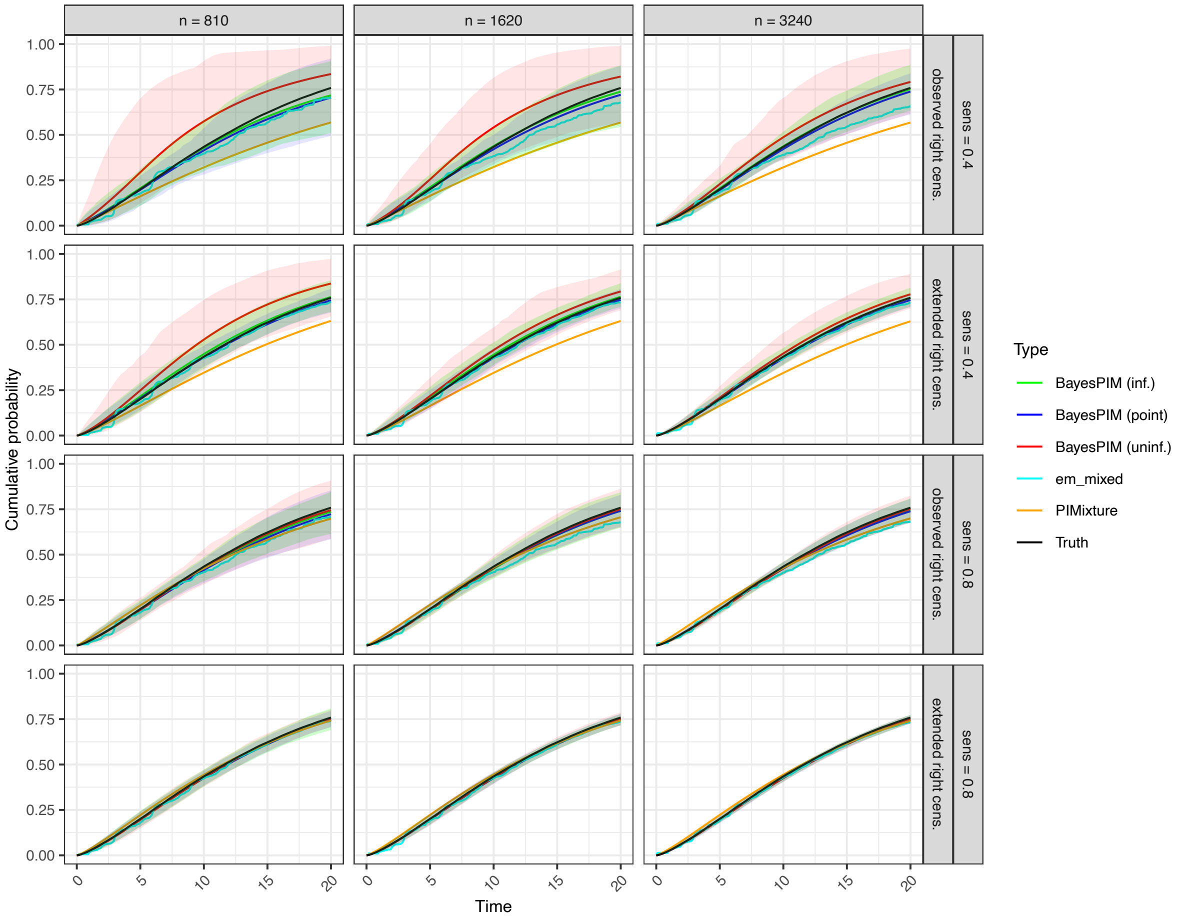

Figure D.5: Marginal CIFs for the non-prevalent population, $F_t(t \mid g = 0, \boldsymbol{\beta}, \sigma)$, point-wise averaged over 200 Monte Carlo simulation runs with 95% quantiles shown as shaded regions. For `BayesPIM` the median of the posterior predictive CIF is used. For `Pimixture` and `em_mixed` the corresponding maximum likelihood estimates are used, rescaled to the non-prevalent population.

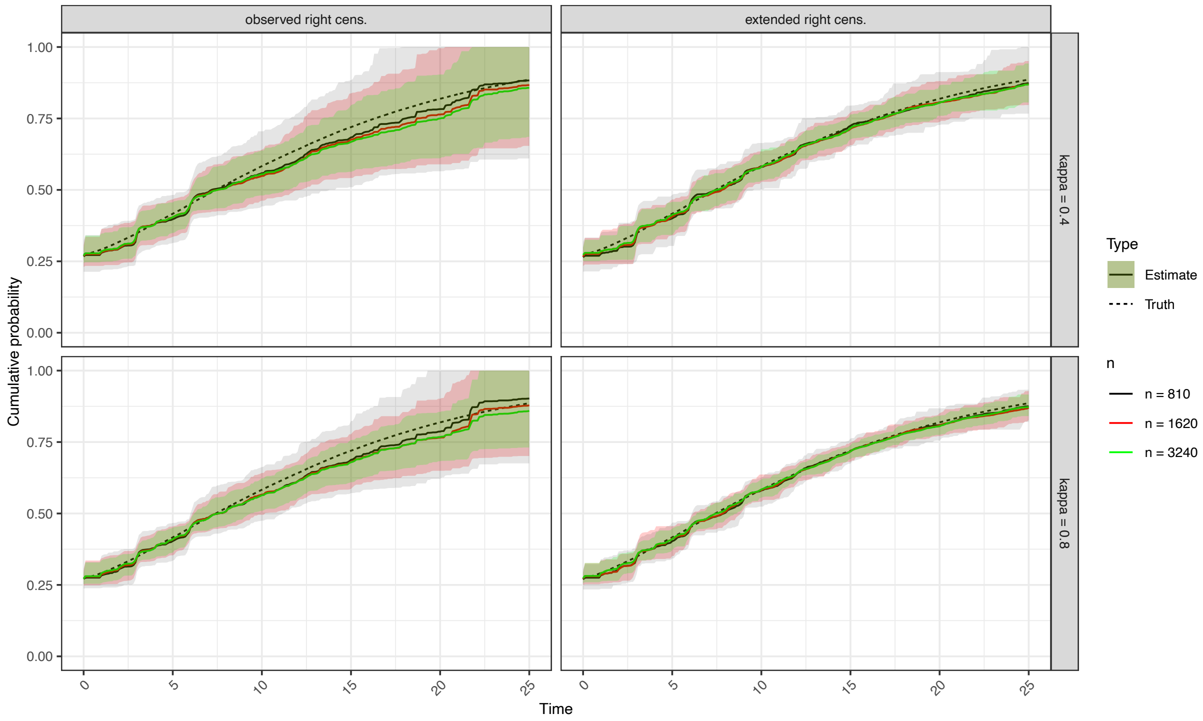

Figure D.6: Marginal mixture CIFs, $F_{t^*}(t \mid \boldsymbol{\beta}, \sigma, \boldsymbol{\theta})$, estimated by `em_mixed`, point-wise averaged over 200 Monte Carlo simulation runs with 95% quantiles shown as shaded regions.

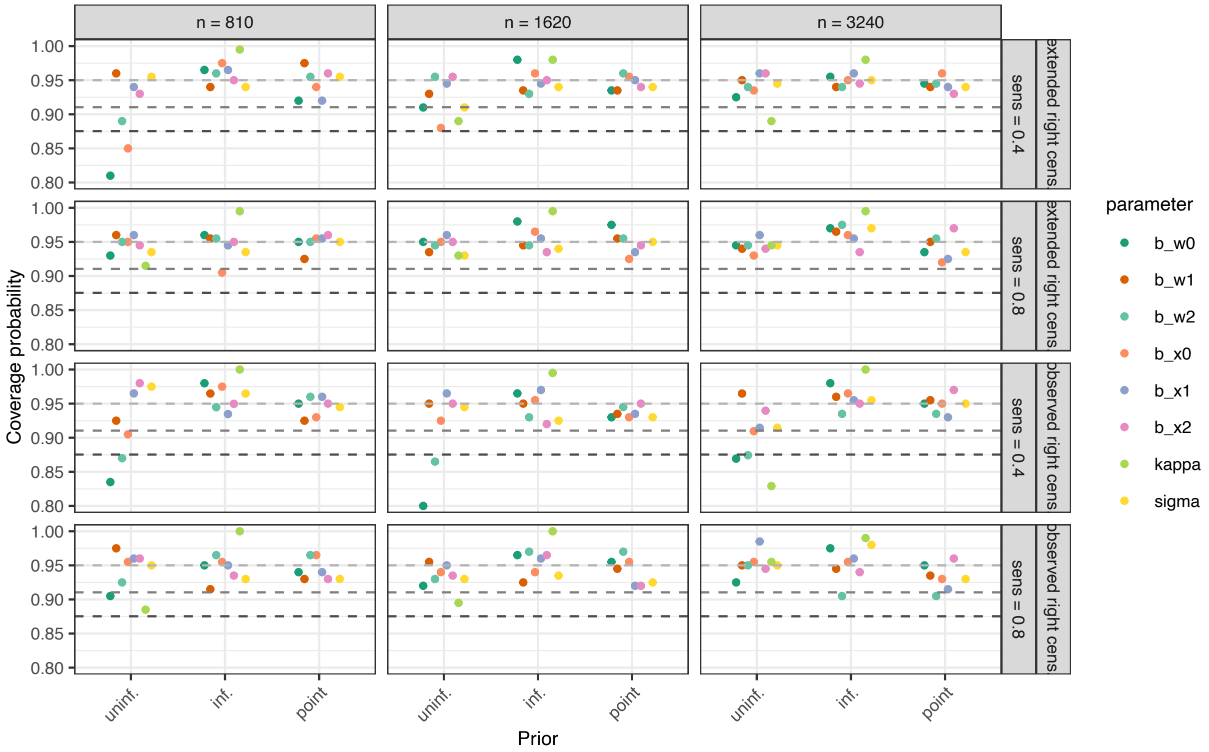

Figure D.7: Frequentist coverage probability of the Bayesian 95% posterior credible intervals for the 36 simulation conditions in Simulation 2 (estimated from 200 Monte Carlo data sets per condition by the proportion of intervals covering the true parameter value). The gray dotted lines in each panel denote, from top to bottom: (a) the nominal 95% level, (b) the value of a point estimate whose 95% confidence upper bound is equal to 95%, (c) the value of a point estimate whose 95% confidence upper bound is equal to 95% with a Bonferroni adjustment for 36 repeated tests.

E Additional results from the CRC application

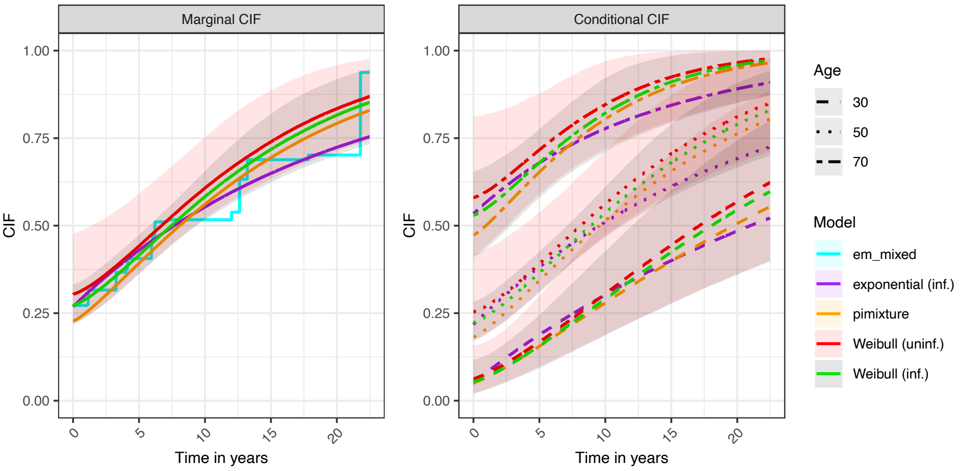

Figure E.1: Marginal and conditional mixture cumulative incidence functions (CIF), $F_{t^*}(t \mid \boldsymbol{\beta}, \sigma, \boldsymbol{\theta})$ and $F_{t^*}(t \mid \tilde{\mathbf{x}}, \boldsymbol{\beta}, \sigma, \boldsymbol{\theta})$, for Weibull `BayesPIM` with uninformative (uninf.) prior and informative (inf.) priors. For comparison, CIF from the Weibull `PIMixture` model, the exponential `BayesPIM` model (inf.), and `em_mixed` are given. The lines represent posterior median estimates and the shaded regions indicates the 95% credible interval of the Weibull (inf. in gray) and Weibull (uninf. in red) models.

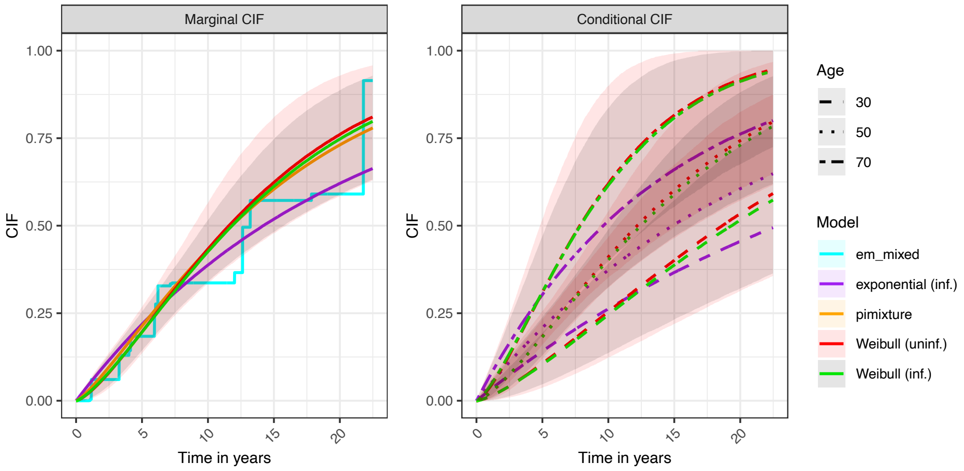

Figure E.2: Marginal and conditional cumulative incidence functions (CIF) for the non-prevalent population, $F_t(t \mid g = 0, \boldsymbol{\beta}, \sigma)$ and $F_t(t \mid g = 0, \tilde{\mathbf{x}}, \boldsymbol{\beta}, \sigma, \boldsymbol{\theta})$, for Weibull `BayesPIM` with uninformative (uninf.) prior and informative (inf.) priors. For comparison, CIF from the Weibull `PIMixture` model, the exponential `BayesPIM` model (inf.), and `em_mixed` are given. The lines represent posterior median estimates and the shaded regions indicates the 95% credible interval of the Weibull (inf. in gray) and Weibull (uninf. in red) models.
