# Supplementary material for: A Bayesian Prevalence‐Incidence Mixture Model for Screening Outcomes With Misclassification
Source: Stat Med. 2026 Apr 7;45(8-9):e70433. doi: 10.1002/sim.70433 (PMC13054642; doi:10.1002/sim.70433)
Supplement: Supplementary file 2 — Rcodes: A zip‐archive with R code, in particular: A tar.gz file with the BayesPIM package (see readme∖_packages). A tar.gz file with the EMmixed package (see readme∖_packages). A zip‐archive with R code and additional documentation (see readme∖_simulations) for running and analyzing the simulation studies. [file SIM-45-0-s002.zip › R_supplement/readme_packages.html]

Readme R packages


# Readme R packages

#### Thomas Klausch

#### 2025-03-06

## Package `BayesPIM`

The package **`BayesPIM`** implements the
Gibbs sampler and several accompanying functions for applying the
methods described in Klausch et al. (2024). A packaged installation
version is available as part of the Supplementary Material in the file
`BayesPIM_1.0.0.tar.gz`. To install from file, use:

```
install.packages("BayesPIM_1.0.0.tar.gz", repos = NULL, type = "source")
```

Alternatively, installation from GitHub is possible:

```
install.packages("devtools")
devtools::install_github("thomasklausch2/BayesPIM", build_vignettes = FALSE)
```

The package contains an accessible vignette (run:
`vignette("BayesPIM_intro")`) providing detailed information
on the primary functions and examples demonstrating package usage,
including estimating prevalence-incidence models, assessing model fit,
and plotting cumulative incidence functions.

The package’s main functions are:

- `bayes.2S_seq`: Fitting Bayesian Prevalence-Incidence
  Mixture Model (sequential processing)
- `bayes.2S`: Fitting Bayesian Prevalence-Incidence Mixture
  Model
- `gen.dat`: Simulate Screening Data for a
  Prevalence-Incidence Mixture Model
- `get.IC_2S`: Compute Information Criteria for a Bayesian
  Prevalence-Incidence Mixture Model
- `get.ppd.2S`: Posterior Predictive Cumulative Incidence
  Function
- `search.prop.sd_seq`: Automated Heuristic Search of
  Proposal Standard Deviation for `bayes.2S` (sequential
  processing)
- `search.prop.sd`: Automated Heuristic Search of Proposal
  Standard Deviation for `bayes.2S`
- `trim.mcmc`: Subset MCMC draws (burn-in and
  thinning)

All functions have dedicated help files (e.g.,
`?bayes.2S`).

## Package `EMmixed`

The package **`EMmixed`** implements the
non-parametric estimator `em_mixed` by Witte et al. (2017),
as well as the extension by Klausch et al. (2024) to handle baseline
prevalence (`np_estimator`). A packaged installation version
is available as part of the Supplementary Material in file
`EMmixed_1.0.0.tar.gz`. To install from file, use:

```
install.packages("EMmixed_1.0.0.tar.gz", repos = NULL, type = "source")
```

Alternatively, installation from GitHub is possible:

```
install.packages("devtools")
devtools::install_github("thomasklausch2/EMmixed")
```

## References

- T. Klausch, B. I. Lissenberg-Witte, and V. M. Coupe (2024). “A
  Bayesian prevalence-incidence mixture model for screening outcomes with
  misclassification.” *arXiv:2412.16065*.
- B. I. Witte, J. Berkhof, and M. A. Jonker (2017). “An EM
  algorithm for nonparametric estimation of the cumulative incidence
  function from repeated imperfect test results,” *Statistics in
  Medicine*, vol. 36, no. 21, pp. 3412–3421. doi: 10.1002/sim.7373.
