# Supplementary material for: A Bayesian Prevalence‐Incidence Mixture Model for Screening Outcomes With Misclassification
Source: Stat Med. 2026 Apr 7;45(8-9):e70433. doi: 10.1002/sim.70433 (PMC13054642; doi:10.1002/sim.70433)
Supplement: Supplementary file 2 — Rcodes: A zip‐archive with R code, in particular: A tar.gz file with the BayesPIM package (see readme∖_packages). A tar.gz file with the EMmixed package (see readme∖_packages). A zip‐archive with R code and additional documentation (see readme∖_simulations) for running and analyzing the simulation studies. [file SIM-45-0-s002.zip › R_supplement/readme_simulations.html]

Readme: BayesPIM Simulations


# Readme: `BayesPIM` Simulations

#### Thomas Klausch

#### 2025-03-06

This file explains the structure of the `BayesPIM` and
`EMmixed` simulations, implemented in `R`, which
are reported in Klausch et al. (2024) as simulation 1 and simulation 2.
We address both simulation set-ups in turn. To install
`BayesPIM` use

```
install.packages("devtools")
devtools::install_github("thomasklausch2/BayesPIM", build_vignettes = FALSE)
```

To install `EMmixed` use

```
install.packages("devtools")
devtools::install_github("thomasklausch2/EMmixed", build_vignettes = FALSE)
```

To install `PIMixture` see https://dceg.cancer.gov/tools/analysis/pimixture.

### Reproducibility of the simulations

As described in detail below, to replicate the simulations, run the
following controller files found in the sub-directories
`sim/sim1`, `sim/sim2`, and
`sim/sim_EMmixed` (with `n_mc` set to the desired
number of simulated data sets per condition and `no_clusters`
set to the desired number of clusters):

- `sim/sim1/sim1_controller.r`: runs `BayesPIM`
  and `PIMixture` on simulation 1
- `sim/sim2/sim2_controller_run.r`: runs
  `BayesPIM` and `PIMixture` on simulation 2 (run
  once for `offset0` and once for `offset10`, see
  details)
- `sim/sim2/sim1_npmle_controller_run.r`: runs
  `EMmixed` on simulation 1
- `sim/sim2/sim2_npmle_controller_run.r`: runs
  `EMmixed` on simulation 2

Note: to run simulation 2 (`sim2_controller_run.r`) the
censoring distribution files `sim2_censdist_offset0.rdata`
and `sim2_censdist_offset10.rdata` need to be located in
director `sim/sim2`. These files are available upon request
from the authors and require signing a data sharing agreement. We do,
however, provide the simulation 2 output files for download, see below
section ‘Online data availability’.

The following output files are generated:

- `sim1_output.rdata`: `BayesPIM` output from
  simulation 1
- `sim2_output_offset0.rdata`: `BayesPIM` output
  from simulation 2 for `offset0`
- `sim2_output_offset10.rdata`: `BayesPIM`
  output from simulation 2 for `offset10`
- `sim1_npmle_output.rdata`: `EMmixed` output
  for simulation 1
- `sim2_npmle_output_offset0.rdata`: `EMmixed`
  output for simulation 2 for `offset0`
- `sim2_npmle_output_offset10.rdata`: `EMmixed`
  output for simulation 2 for `offset10`

These files are analyzed using the supplied analysis scripts (run in
this order):

1. `sim/sim1/sim1_pop.cdf.r`: generates population CIFs for
   simulation 1 for use in results plots
2. `sim/sim2/sim2_pop.cdf.r`: generates population CIFs for
   simulation 2 for use in results plots
3. `sim_npmle_analysis.r`: analyses and summarizes results
   for the `EMmixed` part of the simulations and prepares for
   further analyses in the subsequent scripts
4. `sim1_analysis.r`: analyses and summarizes results for
   the `BayesPIM` and `PIMixture` part of simulation
   1
5. `sim2_analysis.r`: analyses and summarizes results for
   the `BayesPIM` and `PIMixture` part of simulation
   2

A set of pdf output files is produced with plots as shown in the main
manuscript.

### Online data availability

Running the simulations took us about 2-3 weeks on 2 state of the art
64 core virtual machines. We therefore also make the output files
available online for download:

https://surfdrive.surf.nl/files/index.php/s/uDFuoRdhdS9yYRv

After download, unpack the zip foler and copy the files in the
respective simulation folders and run the analyses scripts in the order
indicated above.

### Details on simulation 1

This simulation generates synthetic data using the
`BayesPIM` function `gen.dat` under different
simulation settings. The files for the part of the `BayesPIM`
simulation are located in `sim/sim1/`. The files for the
`EMmixed` simulation are in `sim/sim_EMmixed`. We
address both set-ups respectively in the following. For
`BayesPIM` the main controller file that runs the whole
simulation is located in `sim/sim1/sim1_controller.r`.
Running this file will generate data, run estimation, and save the
output. In the preamble the simulation settings are chosen

```
# Define simulation parameters
n_sim = c(1000, 2000)      # Sample sizes
kappa_sim = c(0.4, 0.8)    # Test sensitivities
kappa_infprior_sim = c(0, 1, 2) # Three types of priors: uninformative, informative, point
prob_r_sim = c(1, 0)            # Probability of baseline test sensitivity
theta_sim = c(0.11, 0.22)       # Theta passed to `theta` in gen.dat
```

These settings replicate simulation 1 in the main paper. Note that
`kappa_sim` gives the considered test sensitivities, while
`kappa_infprior_sim` indicates if the prior on the test
sensitivity kappa should be chosen uninformative (uniform(0,1)),
informative (Beta centered at the true senstivitiy with standard
deviation 0.1), or as a correctly specified point prior (set to true
value). The simulation can be run for a total of `n_mc`
iterations (in the paper 200), and distributed in parallel over
`no_clusters`. We used 64 cores on a virtual machine which
took about 2 weeks to complete.

```
# Run Monte Carlo simulation
n_mc <- 2  # Number of iterations / data sets (increase!)
no_clusters <- 4  # Number of cores (increase!)
```

Internally, the controller runs the workhorses
`sim1_run.r` which does the actual data generation and
running of `BayesPIM` as well as
`sim_run_pimixture.r` which runs estimation by
`PImixture`. After completion the code in
`sim1_analysis.r` was used to summarize and visualize
findings. `sim1_pop.cdf.r` calculates the population
cumulative incidence function using a large sample which is used as part
of the analyses in `sim1_analysis.r`.

In addition, we run `EMmixed` on each data set which is
achieved by running
`sim/sim_EMmixed/sim1_npmle_controller_run.R`. Internally,
the workhorse `sim1_run_npmle.r` runs the
`EMmixed` function `np_estimator` that does
non-parametric estimation of the prevalence-incidence mixture CIF.

### Details on simulation 2

The files for simulation 2 are located in `sim/sim2` for
the `BayesPIM` part and in `sim/EMmixed` for the
EMmixed part. This simulation generates data that are similar in
screening times distributions and covariate distributions to those
observed in the real colorectal cancer screening electronic health
records analysed in Klausch et al. (2024). For this, a list of screening
times `V_m` and a matrix of covariates `Z` have
been prepared in files `sim2_censdist_offset0.rdata` and
`sim2_censdist_offset10.rdata` which have to be located in
directory `sim/sim2`. Note that these files are not available
in the supplementary material for reasons of data porotection (as they
contain the real-world covariates and screening times). These files are,
however, available upon request and signing a data sharing agreement.
Note that the simulation output files are available for download, as
described in section ’Online data availability` above.

`V_m` contains the original screening times supplemented
for additional plausible screening times until right censoring (for
individuals that encountered events), using the procedure described in
detail in the Supplemental Material to Klausch et al. (2024). This
simulation then employs a dedicated function
`sim2_fun_gensimdat.r` to get a sample of size
`n_sim` from `V_m`, simulate prevalence status and
incidence time using parameters fixed at those from the Weibull (inf.)
model in the main paper (contained in vector `est`), and
subsequently determine at which screening time an event is found, given
a set value for the test senstivity `kappa`. The resulting
vector of screening times until an event or right censoring is returned
as `Vobs` which is, subsequently, analyzed by
`BayesPIM`, `PImixture`, and `EMmixed`.
The simulation 2 in Klausch et al (2024) in addition considers an
‘extended right censoring time’ setting, where the time of right
censoring has been artificially delayed for 10 years. The resulting
visiting times are available in
`sim/sim2/sim2_censdist_offset10.rdata`.

Simulation 2 is run by the controller function
`sim2_controller_run.r`. The function first loads packages
and the data generating function. Subsequently, one of the following
lines has to be parsed in (and the other out), depending on which
censoring time setting is simulated.

```
# Load simulation parameters
load('sim/sim2/sim2_censdist_offset0.rdata') # parse in for standard setting
load('sim/sim2/sim2_censdist_offset10.rdata') # parse in for extended censoring setting
```

Subsequently, the input parameters control the set-up of the
simulation. The following values are default and those used by Klausch
et al. (2024).

```
# Define different input parameters
n_sim = c(n_data, n_data*2, n_data*4) # Sample sizes
kappa_sim = c(0.4, 0.8)               # Test sensitivities
kappa_infprior_sim = c(0, 1, 2)       # Three types of priors: uninformative, informative, point
prob_r = prob_r_data                  # Probability of baseline test sensitivity
```

`n_sim` sets the data set size conditions that are
considered, where `n_data` is a scalar loaded through
`sim2_censdist_offset0.rdata` or
`sim2_censdist_offset10.rdata` and is set at the size of the
original data (n=810). `kappa_sim` gives the considered test
sensitivities, while `kappa_infprior_sim` indicates if the
prior on the test sensitivity kappa should be chosen uninformative
(uniform(0,1)), informative (Beta centered at the true senstivitiy with
standard deviation 0.1), or as a correctly specified point prior (set to
true value). In this simulation, we hold the proportion of baseline
tests available at the observed value of 0.93, also loaded through
`sim2_censdist_offset0.rdata` or
`sim2_censdist_offset10.rdata`. As for simulation 1, the
following two lines specify the number of data sets per condition as
well as the number of cores used in parallel computing.

```
# Run Monte Carlo simulation
n_mc <- 200  # Number of iterations
no_clusters <- 64  # Number of cores
```

Internally, the controller runs the workhorses
`sim2_run.r` which does the actual data generation and
running of `BayesPIM` as well as
`sim_run_pimixture.r` which runs estimation by
`PImixture`. After completion the code in
`sim2_analysis.r` was used to summarize and visualize
findings. `sim2_pop.cdf.r` calculates the population
cumulative incidence function using a large sample which is used as part
of the analyses in `sim2_analysis.r`.

In addition, we run `EMmixed` on each data set which is
achieved by running
`sim/sim_EMmixed/sim2_npmle_controller_run.R`. Internally,
the workhorse `sim2_run_npmle.r` runs the
`EMmixed` function `np_estimator` that does
non-parametric estimation of the prevalence-incidence mixture CIF.
