# Supplementary material for: A Bayesian Prevalence‐Incidence Mixture Model for Screening Outcomes With Misclassification
Source: Stat Med. 2026 Apr 7;45(8-9):e70433. doi: 10.1002/sim.70433 (PMC13054642; doi:10.1002/sim.70433)
Supplement: Supplementary file 2 — Rcodes: A zip‐archive with R code, in particular: A tar.gz file with the BayesPIM package (see readme∖_packages). A tar.gz file with the EMmixed package (see readme∖_packages). A zip‐archive with R code and additional documentation (see readme∖_simulations) for running and analyzing the simulation studies. [file SIM-45-0-s002.zip › R_supplement/packages/BayesPIM_1.0.0.tar.gz!/BayesPIM/inst/doc/BayesPIM_intro.html]

Introduction to BayesPIM


# Introduction to BayesPIM

## Introduction

BayesPIM is a versatile modeling environment for screening and
surveillance data, as described in detail in the main reference Klausch
et al. (2024). It is a so-called prevalence-incidence mixture (PIM)
model with a Bayesian Gibbs sampler as the estimation backend. It
requires a specific data structure, as described in detail in the main
references. Specifically, for each individual, a numeric vector of
screening times (starting at 0) is available. In the case of right
censoring, this vector ends in its last position with Inf.

BayesPIM includes a data-generating function that simulates data
according to the model’s assumptions. This is useful for illustration or
testing.

## Data generation

We start by loading BayesPIM:

```
library(BayesPIM)
#> Loading required package: coda
```

and generate data

```
# Generate data according to the Klausch et al. (2024) PIM
set.seed(2025)
dat = gen.dat(
  kappa = 0.7, n = 1e3, theta = 0.2,
  p = 1, p.discrete = 1,
  beta.X = c(0.2, 0.2), beta.W = c(0.2, 0.2),
  v.min = 20, v.max = 30, mean.rc = 80,
  sigma.X = 0.2, mu.X = 5, dist.X = "weibull",
  prob.r = 1
)
```

For all details, see ?gen.dat. Here, we simulate screening data under
an AFT survival (incidence) model:

\[\log(x\_i) = \mathbf{z}\_{xi}'
\mathbf{\beta}\_x + \sigma \epsilon\_i\]

and a Probit prevalence model where prevalence is given when \(g\_i=1\) and absent when \(g\_i=0\), and we use the latent variable
representation \(Pr(g\_i=1 | \mathbf{z}\_{wi}) =
Pr(w\_i > 0 | \mathbf{z}\_{wi})\) to fit the model, where

\[w\_i = \mathbf{z}\_{wi}'
\mathbf{\beta}\_w + \psi\_i\]

with \(\psi\_i\) a standard normal
random variable. `gen.dat` sets the regression coefficients
of the incidence and prevalence model to `beta.X` and
`beta.W` respectively, with `mu.X` and
`sigma.X` the intercept and scale of the AFT model and the
intercept of the prevalence model given by `qnorm(theta)`.
So, `theta` can be interpreted as the conditional probability
of prevalence if all covariates are zero.

Subsequently, a screening series is super-imposed using successive
draws from uniformly distributed random variables, with
`v.min` the minimum and `v.max` the maximum time
between screening moments. The right censoring time is drawn from an
exponential distribution with mean time to right censoring
`mean.rc`. At each screening time, a test is executed if the
event of the time is larger than the true simulated \(x\_i\). Detection is successful with
probability `kappa`, the test sensitivity.
`prob.r` controls the probability that a baseline test is
executed, which we leave at one denoting a baseline test for all
individuals. This data generating mechanism is identical to the one
described in Simulation 1 in Klausch et al. (2024).

```
head(dat$Vobs)
#> [[1]]
#> [1] 0
#> 
#> [[2]]
#> [1]   0.00000  22.53705  51.89452  78.35944 104.52213 131.74624
#> 
#> [[3]]
#> [1]  0.00000 25.37066
#> 
#> [[4]]
#> [1] 0
#> 
#> [[5]]
#> [1]   0.00000  27.37410  53.60168  80.54386 108.43628       Inf
#> 
#> [[6]]
#> [1]  0.00000 22.31108 48.06398 77.10746      Inf
```

We see `dat$Vobs[[1]]` and `dat$Vobs[[3]]` are
prevalent cases positive at baseline. `dat$Vobs[[2]]` has an
event detected at `131.7` and `dat$Vobs[[5]]` and
`dat$Vobs[[6]` are right censored cases (no event). In
addition, covariates `dat$Z` are returned as well as the true
times `dat$X.true` and prevalence status `dat$C`,
which are both not observed in practice (latent), `dat$r`
indicates whether a baseline test was done. Data of this kind is
processed by `bayes.2S`.

## Running the estimation (Gibbs sampler)

The `bayes.2S` function first searches starting values
using a vanilla run that drops all known (confirmed) prevalent cases and
assumes `kappa=1` as well as no prevalence
(`prev=F` setting in `BayesPIM`). This setting is
akin to an interval censored survival regression, which has high
robustness and finds starting values in the neighbourhood of the final
estimates of a run of `BayesPIM` where
`kappa<1` and `prev=T`. Subsequently, the main
Gibbs sampler is started and run for `ndraws`.

The code below shows a straight forward fit to the generated data. In
practice, we advise to normalize any continuous variables, as large
scale in `Z.X` can lead to slow convergence of the Gibbs
sampler, which is not needed here because the generated covariates are
standard normally distributed. The test sensitivity `kappa`
is set to a known value when `update.kappa=F` - otherwise it
is estimated, and then a `kappa.prior` is needed; see below.
The function runs `chains=4` MCMC chains in parallel and
takes about 1 to 3 minutes, depending on machine. If parallel processing
is not desired `parallel = F` is available or
`bayes.2S_seq` can be used which uses a for loop over the
chains.

```
# An initial model fit with fixed test sensitivity kappa (approx. 1-3 minutes, depending on machine)
mod = bayes.2S( Vobs = dat$Vobs,
                Z.X = dat$Z,
                Z.W = dat$Z,
                r= dat$r,
                kappa = 0.7,
                update.kappa = FALSE,
                ndraws= 1e4,
                chains = 4,
                prop.sd.X = 0.008,
                parallel = TRUE,
                dist.X = 'weibull'
)

# Searching starting values by one naive run 
# Starting Gibbs sampler with 3 chains and 5000 iterations.
# Now doing main run. 
# Starting Gibbs sampler with 4 chains and 10000 iterations.
```

Subsequently, we inspect results

```
# Inspect results
mod$runtime # runtime of Gibbs sampler
plot( trim.mcmc( mod$par.X.all, thining = 10) ) # MCMC chains including burn-in
plot( trim.mcmc( mod$par.X.bi, thining = 10) ) # MCMC chains excluding burn-in
summary( mod$par.X.bi)
apply(mod$ac.X, 2, mean) # Acceptance rates per chain
gelman.diag(mod$par.X.bi) # Gelman convergence diagnostics
```

The `coda` package is required for doing these analyses
(attached when loading `BayesPIM`). Function
`trim.mcmc` is a convenient extension to thin and trim an
`mcmc.list` for faster processing.

## Automated search for the proposal s.d.

A proposal standard deviation has to be specified for the Metropolis
step that samples incidence model parameters from the full conditional
distribution. There is no general guidance on this parameter other than
that it is often found in the range of 0.001 to 0.1. Ideally the
acceptance rate of the Metropolis sampler is 23% (which is a general
result for Metropolis-Hastings samplers). Above, we set the
`prop.sd.X` to a good value, but in practice it has to be
searched by trial and error. Therefore, the function
`search.prop.sd` has been desgined which can be used to
automatically search for a good proposal s.d..

If the `prop.sd.X` should be searched automatically, the
function `search.prop.sd` can be used. This function requries
an initial fit by `bayes.2S` as input, typically a few
thousand draws, and then heuristically searches a useful
`search.prop.sd` which yields a Metropolis sampler that has
an acceptance probability in the `acc.bounds.X`, by default
set to `c(0.2, 0.25)`. What the automated search essentially
does is, starting with the initial fit, the `prop.sd.X` is
adapted using a heuristic rule (see `?search.prop.sd`) such
that the acceptance rate likely increases if it is too low or decreases
if it is too high. After an acceptance rate in the interval
`acc.bounds.X` has been obtained, the algorithm double
`ndraws` to reassure on the stability of the result. It does
so a total of `succ.min` times (default: `3`).

```
# An initial model fit with a moderate number of ndraws (here 1e3)
mod.ini = bayes.2S( Vobs = dat$Vobs,
                Z.X = dat$Z,
                Z.W = dat$Z,
                r= dat$r,
                kappa = 0.7,
                update.kappa = F,
                ndraws= 1e3,
                chains = 4,
                prop.sd.X = 0.005,
                parallel = T,
                dist.X = 'weibull'
)

# Searching starting values by one naive run 
# Starting Gibbs sampler with 3 chains and 5000 iterations.
# Now doing main run. 
# Starting Gibbs sampler with 4 chains and 1000 iterations.

# Running the automated search
search.sd <- search.prop.sd(m = mod.ini)

# Iteration 1 
# Acceptance rate was: 0.564 
# prop.sd.X is set to 0.011 
# Iteration 2 
# Acceptance rate was: 0.417 
# prop.sd.X is set to 0.019 
# Iteration 3 
# Acceptance rate was: 0.113 
# prop.sd.X is set to 0.011 
# Iteration 4 
# Acceptance rate was: 0.216 
# Success. Doubling number of MCMC draws: 2000 
# Iteration 5 
# Acceptance rate was: 0.223 
# Success. Doubling number of MCMC draws: 4000 
# Iteration 6 
# Acceptance rate was: 0.222 
# Finished calibrating proposal variance.
```

```
print(search.sd$prop.sd.X)
#> [1] 0.01058928
```

## Model updating

As can be seen from the R-hat statistics obtained using
`gelman.diag`, the sampler has not fully converged yet. To
achieve convergence the model can be updated for another
`ndraws` or for a specified number of draws
`ndraws.update`.

```
# Model updating
mod_update = bayes.2S( prev.run = mod ) # ndraws additional MCMC draws

# Updating previous MCMC run. 
# Starting Gibbs sampler with 4 chains and 10000 iterations.

mod_update = bayes.2S( prev.run = mod, ndraws.update = 1e3 ) # ndraws.update additional MCMC draws

# Updating previous MCMC run. 
# Starting Gibbs sampler with 4 chains and 1000 iterations.
```

In addition the argument `update.till.converge = T` allows
`bayes.2S` to run until convergence directly, which involves
repeated internal calles to `bayes.2S` to obtain updates if
convergence has not been attained yet. Convergence is attained when the
Gelman-Rubin convergence diagnositic \(\hat{R}
< 1.1\) for all parameters and the minimum effective sample
size `min_effs` is reached. If
`update.till.converge = T`, the sampler continues updating
until convergence is attained or `maxit` is reached.

## Cumulative Incidence Functions

Cumulative incidence functions (CIFs) indicate the cumulative
probability to progress until a specified point in time.
`BayesPIM` offers two different type of CIFs which differ in
how they handle prevalence. First, mixture CIFs depict prevalence as a
point-probability mass at zero. Second, non-prevalence (healthy
population) CIFs depict the CIF for the stratum of the population that
is healthy (disease free) at baseline. Hence, mixture CIFs start at a
probability higher than zero at time zero, whereas non-prevalence CIFs
start at zero (like ‘usual’ CIFs do).

For both mixture CIFs and non-prevalence CIFs we can estimate a
marginal variant which integrates over all model covariates or a
conditional variant which integrates over no or only a subset of
covariates. A marginal CIF can be interpreted as the CIF for a randomly
selected individual from the population, while a conditional CIF gives
the CIF for a randomly selected individual from a subset defined by a
combination of covariates (e.g. male 60 year olds).

To obtain CIF estimates, the function `get.ppd.2S` is
employed. We first obtain the two types of marginal CIFs:

```
# Get posterior predictive marginal CIF, we provide percentiles and obtain quantiles
cif_nonprev <- get.ppd.2S(mod, pst.samples = 1e3, type = 'x', 
                          ppd.type = "quantiles", perc = seq(0, 1, 0.01))
cif_mix     <- get.ppd.2S(mod, pst.samples = 1e3, type = 'xstar', 
                          ppd.type = "quantiles", perc = seq(0, 1, 0.01))
```

The argument `type` controls the type CIF depicted. Here
`xstar` denotes the mixture of prevlant and incident cases
and `x` the CIF of the healthy sub-group. In princple, CIFs
can be obtained in two ways, specified through `ppd.type`:
either specify a set of percentiles `perc` for which
quantiles are returned, or specify a set of quantiles `quant`
for which percentiles are returned. Above, we show the inference via
percentiles which requires to specify
`ppd.type = 'quantiles'` to indicate that quantiles should be
returned. An arbitrary narrow grid of percentiles can be supplied but we
found that `seq(0, 1, 0.01)` is sufficiently narrow.

The argument `pst.samples` gives the precision of
inference. Note that the computation of CIF’s requires selecting a
random sample of posterior draws of the parameters (default:
`1000`). For each unique draw one CIF is obtained and
subsequently the median (returned as list entry
`cif_nonprev$med.cdf`) and 95% credible intervals
(`cif_nonprev$med.cdf.ci`) are determined point-wise at
`perc` (or `quant`). Hence, more precise inference
is obtained through higher values of `pst.samples` which,
however, increases computational cost.

Subsequently, we can plot the results as follows.

```
# Comparison plot non-prevalent stratum CIF vs. mixture CIF (marginal)
oldpar <- par(no.readonly = TRUE)
par(mfrow = c(1,2))

plot(cif_nonprev$med.cdf, cif_nonprev$perc, ty = 'l', ylim=c(0,1), 
     xlim=c(0,300), xlab = 'Time', ylab ='Cum. prob.', main = 'Non-prevalence CIF')
lines(cif_nonprev$med.cdf.ci[1,], cif_nonprev$perc, lty=2, col=2)
lines(cif_nonprev$med.cdf.ci[2,], cif_nonprev$perc, lty=2, col=2)
plot(cif_mix$med.cdf, cif_nonprev$perc, ty = 'l', ylim=c(0,1), 
     xlim=c(0,300), xlab = 'Time', ylab ='Cum. prob.', main = 'Mixture CIF')
lines(cif_mix$med.cdf.ci[1,], cif_nonprev$perc, lty=2, col=2)
lines(cif_mix$med.cdf.ci[2,], cif_nonprev$perc, lty=2, col=2)
```

The same result is obtaiend via `ppd.type = "quantiles"`,
but now `quant` has to be passed in a useful range of values.
If it is unknown which value range is useful, the inference approach via
`ppd.type = "percentiles"` is more straightforward, as it
only requires specifying a grid of percentiles between 0 and 1 and any
associated quantiles are returned.

```
# Alternatively, we can provide quantiles and obtain percentiles
cif2_nonprev <- get.ppd.2S(mod, pst.samples = 1e3, type = 'x', 
                           ppd.type = "percentiles", quant = 1:300)
cif2_mix     <- get.ppd.2S(mod, pst.samples = 1e3, type = 'xstar', 
                           ppd.type = "percentiles", quant = 1:300)
```

However, the inference approach via
`ppd.type = "percentiles"` is still useful if probabilities
of transition for specific quantiles are of interest. For example, if
the mixture CIF should be evaluated at zero and 100 time units
(e.g. days), the following approach is useful to obtain prevalence
estimates and incidence estimates at 100 time units.

```
get.ppd.2S(mod, pst.samples = 1e3, type = 'xstar', ppd.type = "percentiles", quant = c(0,100))
```

```
print(quants)
#> $med.cdf
#> [1] 0.241 0.360
#> 
#> $med.cdf.ci
#>           [,1]  [,2]
#> 2.5%  0.202000 0.311
#> 97.5% 0.278025 0.409
#> 
#> $quant
#> [1]   0 100
```

While the above discussed CIFs are marginal, i.e. they integrate over
all underlying covariates, it is also possible to condition CIFs on all
or a subset of covariates passed in `Z.X` and
`Z.W`. For this, the `get.ppd.2S` arguments
`fix_Z.X` and `fix_Z.W` are employed. If the
columns (covariates) in `Z.X` and `Z.W` are
identical, only `fix_Z.X` has to be employed as demonstrated
by the following example.

```
# Conditional CIFs, example conditional mixture CIF integrating over one covariate
cif_mix_m1 <- get.ppd.2S(mod, fix_Z.X = c(-1,NA), pst.samples = 1e3, type = 'xstar', 
                         ppd.type = "quantiles", perc = seq(0, 1, 0.01))
cif_mix_0  <- get.ppd.2S(mod, fix_Z.X = c(0,NA), pst.samples = 1e3, type = 'xstar', 
                         ppd.type = "quantiles", perc = seq(0, 1, 0.01))
cif_mix_p1 <- get.ppd.2S(mod, fix_Z.X = c(1,NA), pst.samples = 1e3, type = 'xstar', 
                         ppd.type = "quantiles", perc = seq(0, 1, 0.01))
```

Here we get three conditional CIFs fixing the first covariate in the
incidence and prevalence model at the levels -1, 0, and 1, respectively,
while inegrating over the second covariate which is indicated by setting
this entry to `NA`. Subsequently a comparative plot is
obtained via

```
# Plot of CIFs for three levels of the first covariate
par(mfrow = c(1,1))
plot(cif_mix_m1$med.cdf, cif_mix_m1$perc, ty = 'l', ylim=c(0,1), 
     xlim=c(0,300), xlab = 'Time', ylab ='Cum. prob.', main = 'Conditional mixture CIF')
lines(cif_mix_0$med.cdf, cif_mix_m1$perc, col=2)
lines(cif_mix_p1$med.cdf, cif_mix_m1$perc, col=3)

par(oldpar)
```

If the columns in `Z.X` and `Z.W` differ, care
has to be taken in fixing the covariates to the right values. Suppose,
for example `Z.X` contains the variables age, gender, and
education, while `Z.W` contains age and education, then the
vector `fix_Z.X` conditioning the incidence model used for
the CIF would be, for example `c(60,0,1)`, denoting 60 years,
male, and high education, so that the prevalence model would need to be
conditioned through fixing `fix_Z.X = c(60, 1)`. Here, gender
would be omitted because it was not part of the prevalence model.
However, we believe that more commonly the incidence and prevalence
models contain the same covariates, so that the approach of conditioning
via `fix_Z.X` only, described above, likely is more commonly
used.

## Information Criteria

Bayesian information criteria are a straightforward approach for
model selection. The function `get.IC_2S.r` returns the
Widely Applicable Information Criterion (WAIC-1/-2) and the Divergence
Information Criterion (DIC), as defined in Gelman et al. (2014). The
information criteria are useful for selecting the best distribution for
the incidence times. `bayes.2S` offers the Weibull,
log-logistic, and normal distributions. In addition, we can obtain an
exponential transition time through selecting a Weibull model and
constraining the AFT scale parameter \(\sigma\) to one. In `bayes.2S`
this is achieved through setting `dist='weibull`,
`sig.prior.X = 1`, and `fix.sigma.X=T`. To
illustrate, we will compare the Weibull model fitted above with an
exponential model. Note that the resulting exponential model is
equivalent to a Markov model.

```
# An exponential model
mod_exp = bayes.2S( Vobs = dat$Vobs,
                Z.X = dat$Z,
                Z.W = dat$Z,
                r= dat$r,
                kappa = 0.7,
                update.kappa = F,
                ndraws= 1e4,
                chains = 4,
                prop.sd.X = 0.008,
                parallel = T,
                fix.sigma.X = T,
                sig.prior.X = 1,
                dist.X = 'weibull'
)

# Searching starting values by one naive run 
# Starting Gibbs sampler with 3 chains and 5000 iterations.
# Now doing main run. 
# Starting Gibbs sampler with 4 chains and 10000 iterations.

# Get information criteria
get.IC_2S(mod, samples = 1e3)

# > IC_weib
#         WAIC1    WAIC2      DIC
# [1,] 2083.599 2083.694 2083.359

get.IC_2S(mod_exp, samples = 1e3)

# > IC_exp
#         WAIC1    WAIC2      DIC
# [1,] 2392.869 2392.945 2393.931
```

As can be seen, the exponential model has higher information criteria
values, suggesting that the Weibull model has better fit. This is
plausible, since the data were generated from a Weibull
distribution.

## References

Gelman, A., Hwang, J., & Vehtari, A. (2014). Understanding
predictive information criteria for Bayesian models. Stat Comput, 24(6),
997–1016. https://doi.org/10.1007/s11222-013-9416-2

T. Klausch, B. I. Lissenberg-Witte, and V. M. Coupe (2024). “A
Bayesian prevalence-incidence mixture model for screening outcomes with
misclassification.” arXiv:2412.16065.
